# Rg3-lipo biomimetic delivery of paclitaxel enhances targeting of tumors and myeloid-derived suppressor cells

**DOI:** 10.1172/JCI178617

**Published:** 2024-11-15

**Authors:** Yuru Shen, Bin Zhong, Wanwei Zheng, Dan Wang, Lin Chen, Huan Song, Xuanxuan Pan, Shaocong Mo, Bryan Jin, Haoshu Cui, Huaxing Zhan, Feifei Luo, Jie Liu

**Affiliations:** 1 Department of Digestive Diseases and National Clinical Research Center for Aging and Medicine, Huashan Hospital, Fudan University, Shanghai, China.; 2 Biotherapy Research Center, Fudan University, Shanghai, China.; 3 Xiamen Ginposome Pharmaceutical Co. Ltd., Xiamen, China.

**Keywords:** Immunology, Therapeutics, Drug therapy, Immunotherapy

## Abstract

Liposomal drug delivery systems have revolutionized traditional cytotoxic drugs. However, the relative instability and toxicity of the existing liposomal drug delivery systems compromised their efficacy. Herein, we present Rg3-lipo, an innovative drug delivery system using a glycosyl moiety–enriched ginsenoside (Rg3). This system is distinguished by its glycosyl moieties exposed on the liposomal surface. These moieties imitate human cell membranes to stabilize and evade phagocytic clearance. The Rg3-lipo system loaded with paclitaxel (PTX-Rg3-lipo) demonstrated favorable bioavailability and safety in Sprague-Dawley rats, beagle dogs, and cynomolgus monkeys. With its glycosyl moieties recognizing tumor cells via the glucose transporter Glut1, PTX-Rg3-lipo inhibited gastric, breast, and esophageal cancers in human cancer cell lines, tumor-bearing mice, and patient-derived xenograft models. These glycosyl moieties selectively targeted myeloid-derived suppressor cells (MDSCs) through the glucose transporter Glut3 to attenuate their immunosuppressive effect. The mechanism study revealed that Rg3-lipo suppressed glycolysis and downregulated the transcription factors c-Maf and Mafb overcoming the MDSC-mediated immunosuppressive microenvironment and enhancing PTX-Rg3-lipo’s antitumor effect. Taken together, we supply substantial evidence for its advantageous bioavailability and safety in multiple animal models, including nonhuman primates, and Rg3-lipo’s dual targeting of cancer cells and MDSCs. Further investigation regarding Rg3-lipo’s druggability will be conducted in clinical trials.

## Abstract 2

Rg3-lipo biomimetic delivery system displays safe and efficacy by dual targets.

## Introduction

Cytotoxic drugs, employed as conventional approaches in cancer therapy, have shown certain efficacy; however, susceptibility to multidrug resistance, lack of targeting specificity, and severe toxic effects have limited their clinical use and therapeutic outcomes (1). Liposomal drug delivery has emerged as a promising approach to address these challenges (2, 3).

Liposomal drug delivery systems can enhance drug stability, diminish drug toxicity, and achieve targeted drug delivery, thereby enhancing therapeutic efficacy (4). Despite the potential offered by liposomal drug delivery systems, various challenges persist, including relative instability, safety limitations, potential immunotoxicity, and the complexity of the tumor microenvironment, which constrain conventional liposomal application (5–7). Although using cholesterol and polyethylene glycol as liposomal membrane materials can extend the circulation time and stabilize the liposome structure, there are also drawbacks. Liposomes prepared by traditional cholesterol have large particle sizes and poor stability, making it challenging to meet the stability requirements of drug formulations and severely limiting their applications. Although the use of polyethylene glycol can prolong the circulation of the liposome, this will lead to the drug not being released in time, and the drug accumulation in the tissue is insufficient to achieve the anticipated therapeutic effect (8–10). These strategies are associated with several drawbacks that compromise druggability (9, 11). Consequently, it is imperative to develop liposome membrane materials to optimize the functionality of liposomes and improve their efficacy in clinical applications.

Previous studies have mostly concentrated on evaluating the safety of allergic reactions during the augmentation of paclitaxel (PTX). While liposomal formulations have successfully reduced the incidence of adverse reactions, they have not substantially enhanced treatment efficacy in clinical settings (12, 13). This is because recent advances have only partially reduced the general adverse effects of PTX, while the safety and regulation of the immune system have been largely overlooked. The tumor microenvironment, governed by dynamic interactions between tumors and immune cells, plays a crucial role in modulating immune responses (14). The late-stage tumor immune microenvironment is characterized by a scarcity of functional immune cells and an accumulation of inhibitory immune cells, particularly myeloid-derived suppressor cells (MDSCs) (15, 16), a heterogeneous subset of myeloid cells (17). Within the tumor microenvironment, the normal maturation of common myeloid progenitors is hindered, leading to the accumulation of MDSCs, thereby suppressing the activation of human and murine T cells (18). MDSCs promote tumor angiogenesis, invasion, and metastasis while generating immunosuppressive cytokines, compromising T cell function (19). Owing to the accumulation of MDSCs and inadequate T cell activation, most patients with cancer do not benefit from anticancer therapies (20). Existing liposome drug delivery systems either lack immunomodulatory capabilities or fail to specifically target MDSCs, rendering them incapable of reversing immune suppression and regulating the tumor immune milieu.

Ginsenoside (Rg3), one of the principal bioactive constituents of ginseng, possesses distinctive biological properties, enabling T and B cell activation, promoting cytokine secretion, and augmenting overall immune function in the body (21). Rg3 exhibits a favorable pharmacokinetics and safety profile with minimal toxic side effects; however, its clinical application has been curtailed by various limitations such as poor solubility, instability, short half-life, and low bioavailability. To address these limitations, we innovatively replaced cholesterol in liposomes with glycosyl moiety–enriched ginsenosides as the membrane material for liposomes, creating a drug delivery system, termed Rg3-lipo, based on the structural similarity between ginsenosides and cholesterol. Unlike conventional cholesterol-based liposomes, ginsenosides’ hydrophobic core can be embedded within the liposome membrane, exposing hydrophilic glycosyl moieties on the liposome surface, thereby allowing biomimetic modifications. In addition, Rg3, with higher stability and more glycosyl moieties than Rg5 and Rh2, is preferred for liposome modification to extend circulation half-life, target tumor cells, and limit adverse effects (22–26).

To accelerate the clinical translation of Rg3-lipo, we carefully evaluated the pharmacokinetics safety profile of Rg3-lipo in diverse animal models (rats, beagle dogs, and cynomolgus monkeys), and also investigated antitumor and immunomodulatory effects of Rg3-lipo both in vitro and in vivo. The glycosylated ginsenoside biomimetic liposomal drug delivery system exhibited comprehensive safety in the central nervous, respiratory, and cardiovascular systems without any toxicity on hematology, coagulation function, serum biochemistry, and gross anatomy, specifically the immune system, and a notable improvement in enhancing antitumor effects and overcoming the immunosuppressive tumor microenvironment. Therefore, it holds great promise as an effective platform for delivering cytotoxic drugs and has considerable potential for clinical translation in cancer therapy.

## Results

### Pharmacokinetics and safety profile of PTX-Rg3-lipo treatment in animal models.

**Figure 1:**
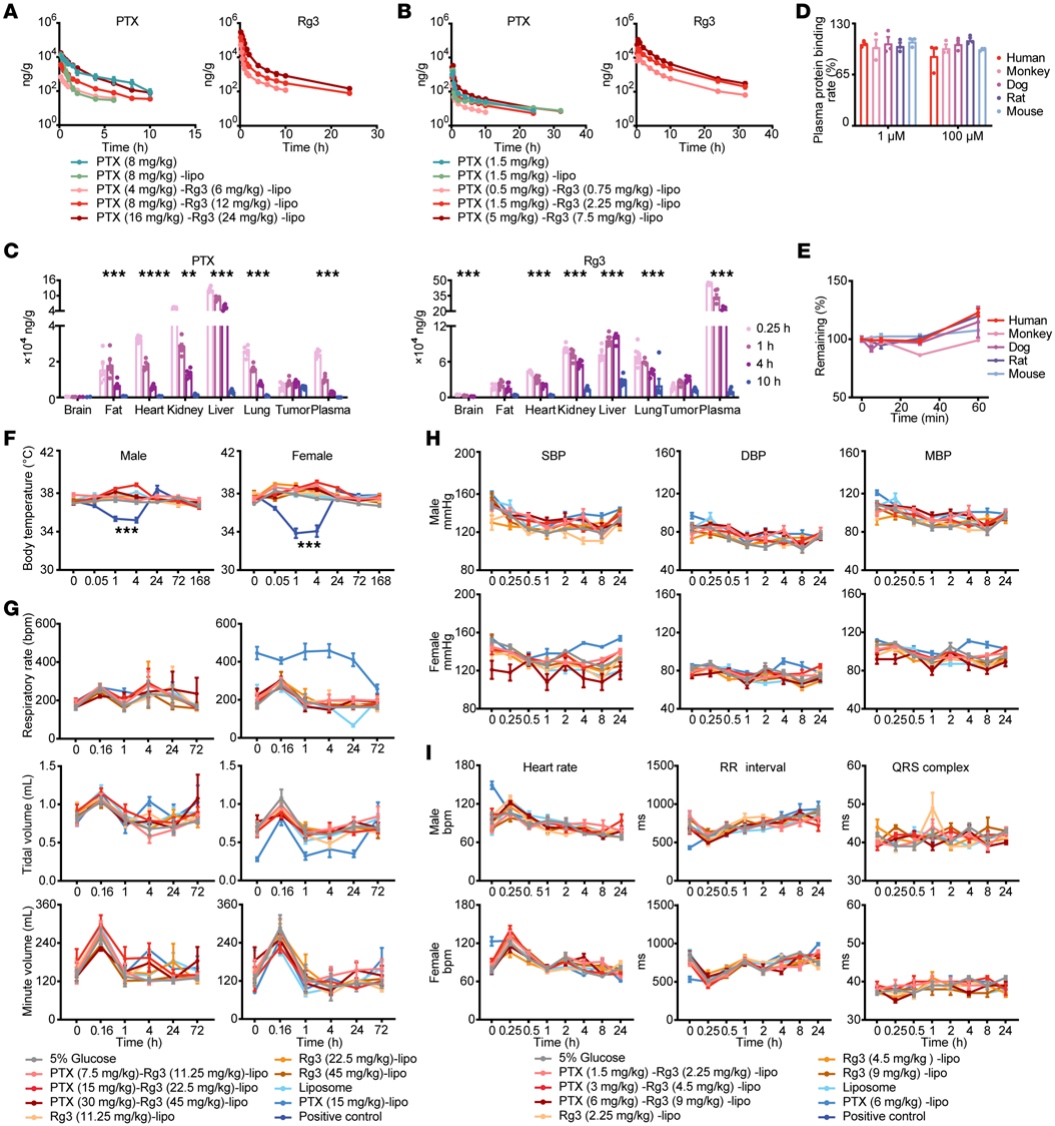
Pharmacodynamics and safety profile of PTX-Rg3-lipo. (**A** and **B**) Single i.v. pharmacokinetic experiment in SD rats (*n* = 5) and beagle dogs (*n* = 6). (**C**) Tissue PTX and Rg3 concentrations following a single i.v. injection of PTX-Rg3-lipo (30 mg/kg PTX and 45 mg/kg Rg3) in human gastric cancer SNU-16 subcutaneous transplantation mouse model (*n* = 6). (**D**) Detection of Rg3 metabolites in human, cynomolgus monkey, beagle dog, SD rat, and mouse exogenous plasma incubation systems. (**E**) Percentage of Rg3 remaining at the metabolic reaction time point relative to the initial moment in human, cynomolgus monkey, beagle dog, SD rat, and mouse liver microsomal culture systems. (**F**) Effects of PTX-Rg3-lipo on conscious unrestrained SD rats’ central nervous systems using modified Irwin tests (*n* = 5). (**G**) Effects of PTX-Rg3-lipo on SD rats’ respiratory systems were measured by whole-body volumetric tracing system (*n* = 5). (**H** and **I**) Effects of PTX-Rg3-lipo on beagle dogs’ cardiovascular system using blood pressure and electrocardiogram tests with telemetry implants (*n* = 4). SBP, systolic blood pressure; DBP, diastolic blood pressure; MBP, mean blood pressure. Data are shown as mean ± SEM. One-way ANOVA (**C** and **D**) and 2-way ANOVA (**A**, **B**, and **E**–**I**) with post hoc Bonferroni’s test were used for statistical analysis. ***P < 0.001.

To determine the pharmacokinetics of PTX-Rg3-lipo, Sprague-Dawley (SD) rats, beagle dogs, and cynomolgus monkeys (*Macaca fascicularis*) were used to assess the absorption, distribution, metabolism, and elimination of PTX-Rg3-lipo. The primary pharmacokinetic parameters were determined in SD rats and beagle dogs (Figure 1, A and B). After administering the corresponding doses of intravenous (i.v.) PTX-Rg3-lipo (calculated as PTX), we observed no sex-based differences in the main pharmacokinetic parameters of Rg3 and PTX between male and female animals. The plasma exposure of Rg3, measured by the maximum observed plasma concentration (C_max_), area under the concentration-time curve from time 0 to the last measurable concentration (AUC_last_), and area under the concentration-time curve extrapolated to infinity based on observed data (AUC_INF_obs_), increased proportionally with increasing dosages within a certain dose range of PTX-Rg3-lipo (calculated as Rg3; 6–24 mg/kg in SD rats, 0.75–7.5 mg/kg in beagle dogs). In SD rats, the increase in plasma PTX exposure was greater than the increase in dose within the 4–16 mg/kg range of PTX-Rg3-lipo (calculated as PTX). Meanwhile, in beagle dogs, the plasma exposure of PTX increased proportionally with increasing dosage within the 0.5–5 mg/kg range of PTX-Rg3-lipo. A difference in plasma exposure to PTX in beagle dogs was observed after i.v. administration of a 1.5 mg/kg dose of PTX-Rg3-lipo and PTX-lipo. Next, we evaluated the in vivo distribution of PTX and Rg3, the principal constituents of PTX-Rg3-lipo. Main organs, tumors, and plasma were collected at different time points after a single injection of PTX-Rg3-lipo. Notably, both PTX and Rg3 (PTX-Rg3-lipo or Rg3-lipo) exhibited extensive distribution across organs, tumor sites, and plasma in tumor-bearing mice (Figure 1C and Supplemental Figure 1; supplemental material available online with this article; https://doi.org/10.1172/JCI178617DS1). Subsequently, we investigated the stability of Rg3 in the plasma of humans, cynomolgus monkeys, beagle dogs, SD rats, and mice ex vivo using liquid chromatography–tandem mass spectrometry (LC-MS/MS) and showed that Rg3 exhibited remarkable stability in the plasma of these species, with no discernible metabolites detected after incubation for 120 minutes (Figure 1D). To study the metabolic stability of Rg3 in liver microsomes from humans, monkeys, dogs, rats, and mice, the metabolic reactions of Rg3 (1 μM) with liver microsomes were incubated at 37°C, and then the reactions were terminated at 0, 5, 10, 30, and 60 minutes, respectively. The experimental results indicated that the half-life of Rg3 in monkey liver microsomes was determined to be 1,386 minutes, while in human, dog, rat, and mouse liver microsomes, it exceeded 6,930 minutes (Figure 1E). Additionally, the intrinsic clearance of Rg3 in monkey liver microsomes was measured to be 1.00 μL/min/mg, whereas in human, dog, rat, and mouse liver microsomes, it was found to be less than 0.2 μL/min/mg. The results of the metabolic stability assay indicated that Rg3 exhibited minimal metabolism in liver microsomes across various species, including humans, monkeys, dogs, rats, and mice. This suggests that Rg3 is not susceptible to degradation by hepatic drug-metabolizing enzymes, specifically hepatic cytochrome P450. Furthermore, no significant hepatotoxicity was observed, indicating that Rg3 possesses desirable stability, high bioavailability, and a favorable safety profile. We explored the excretory routes of PTX and Rg3 in SD rats after a single i.v. injection. Using LC-MS/MS methodologies, we quantified the concentrations of PTX and Rg3 in the bile, feces, and urine and calculated the excretion rates and quantities over time intervals (Supplemental Figure 2). These results suggested that PTX is primarily excreted via the fecal route, whereas Rg3 is predominantly excreted through bile and feces. Overall, the comprehensive pharmacokinetic results indicated that PTX-Rg3-lipo possessed intravenous bioavailability and tissue distribution similar to those of PTX and PTX-lipo.

To determine the safety of PTX-Rg3-lipo, we evaluated its toxicity in the central nervous, respiratory, and cardiovascular systems in SD rat and beagle dog models. First, we sought to understand the effect of PTX-Rg3-lipo on the central nervous system of SD rats (Supplemental Tables 1–15). Irwin observation test results revealed that the positive control group, following the injection of 12 mg/kg chlorpromazine hydrochloride, exhibited substantial central nervous system suppression and altered neurological behavior within 3–4 hours, with a significant drop in body temperature (Figure 1F). These changes aligned with the expected pharmacological impact of chlorpromazine hydrochloride, verifying the ability of our experimental system to accurately and reliably assess the influence of experimental substances on the central nervous system (27). Within the remaining treatment cohorts, no clinically meaningful changes in neurobehavioral profiles or body temperature were detected, suggesting the absence of meaningful toxicological responses within the central nervous system. These results demonstrated that a single i.v. administration of PTX-Rg3-lipo, Rg3-lipo, or PTX-lipo did not trigger observable central nervous system abnormalities in SD rats. The no-observed-adverse-effect level (NOAEL) of PTX-Rg3-lipo and Rg3-lipo on the central nervous system of SD rats was 30 mg/kg (PTX concentration) and 45 mg/kg (Rg3 concentration). We used a conscious, unrestrained SD rat model to determine the effect of PTX-Rg3-lipo on the respiratory system. Single i.v. doses of PTX-Rg3-lipo were administered to rats, and respiratory parameters (i.e., respiratory rate, tidal volume, and minute ventilation) were monitored and scrutinized at various time points before (0 hours) and after administration via whole-body plethysmography (Figure 1G). No postadministration anomalies in the respiratory rate, tidal volume, or minute ventilation linked to treatment were detected across all animal cohorts. These observations suggest the absence of marked respiratory damage in SD rats following PTX-Rg3-lipo administration. The NOAEL of PTX-Rg3-lipo and Rg3-lipo on the respiratory system of SD rats was 30 mg/kg (PTX concentration) and 45 mg/kg (Rg3 concentration) in this study. 

Also, we evaluated the possible effects of PTX-Rg3-lipo on the cardiovascular system by administering a single i.v. injection of PTX-Rg3-lipo to conscious, non-restrained beagle dogs and collected and analyzed cardiovascular data, including mean arterial pressure, diastolic blood pressure, systolic blood pressure, heart rate, and electrocardiography parameters (Figure 1, H and I, and Supplemental Figure 3A). No alterations in cardiovascular metrics associated with the administration of PTX-Rg3-lipo were observed. These results show that PTX-Rg3-lipo has no substantial negative effects on the cardiovascular system of beagle dogs. The NOAEL of PTX-Rg3-lipo and Rg3-lipo in the cardiovascular system of beagle dogs was 6 mg/kg (PTX concentration). To further evaluate the acute toxicity responses of PTX-Rg3-lipo, a single dose was administered to beagle dogs, and their hematology, coagulation function, serum biochemistry, immune function, and gross anatomical parameters were evaluated before administration and after 21 days. The results showed no apparent PTX-Rg3-lipo–related abnormalities in beagle dogs, including blood cell profiles, coagulation function, hepatic and renal toxicity, nutritional status, and gross anatomical observations (Supplemental Figure 3, B–S). It is worth noting that PTX-Rg3-lipo did not cause any significant impairment of the immune system in beagle dogs (Supplemental Figure 3T). Therefore, under the conditions of this experiment, the maximum tolerated dose of i.v. injection of PTX-Rg3-lipo in beagle dogs was determined to be 8 mg/kg. To assess the potential toxicity of PTX-Rg3-lipo on both the host’s immune system and normal intestinal epithelial cells, we conducted apoptosis and proliferation experiments. The results indicated that PTX-Rg3-lipo had no detrimental effect since it did not promote proliferation or induce apoptosis in peripheral blood immune cells and normal intestinal epithelial cells (Supplemental Figure 4). Collectively, the absence of adverse events after administration of PTX-Rg3-lipo suggests a promising pharmacokinetics and safety profile, specifically with regard to the lack of immunotoxicity, providing a solid foundation for further investigation of its therapeutic potential.

### PTX-Rg3-lipo inhibits tumor growth in both human tumor cell lines and patient-derived xenograft models.

**Figure 2:**
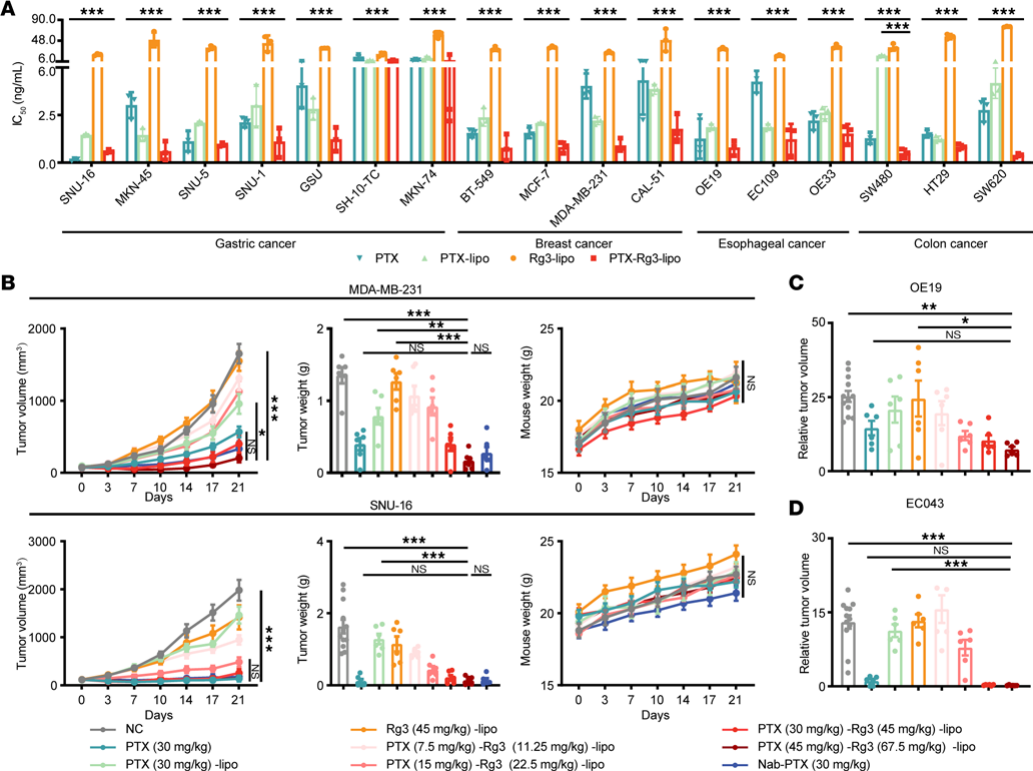
Antitumor effects of PTX-Rg3-lipo in xenograft murine models. (**A**) IC_50_ values of PTX-Rg3-lipo for the inhibition of cell proliferation in various cell lines. (**B**) Tumor growth curves, tumor weights, and mouse weights in nude mice with subcutaneously transplanted human gastric and breast cancer tumors (negative control group, *n* = 12; other groups, *n* = 6). (**C**) Tumor weights following PTX-Rg3-lipo treatment in subcutaneously transplanted human esophageal cancer tumors (*n* = 6). (**D**) PDX model subcutaneously transplanted tumors in nude mice (*n* = 6). Data are shown as mean ± SEM. One-way ANOVA (**A** and tumor weights in **B**–**D**) and 2-way ANOVA (tumor volumes and mouse weights in **B**) with post hoc Bonferroni’s test were used for statistical analysis. *P < 0.05; **P < 0.01; ***P < 0.001.

To explore the efficacy of PTX-Rg3-lipo in eliciting antitumor activity against humanized tumors, a total of 17 human tumor cell lines derived from diverse cancer tissue origins, namely gastric, breast, esophageal, and colon cancers, were selected. Each cell line was subjected to separate interventions to examine its response and potential therapeutic implications (Figure 2A). The results reveal a significantly stronger tumor cell–inhibitory effect of PTX-Rg3-lipo than of an equivalent concentration of PTX and PTX-lipo, which underscores the enhanced potency of PTX-Rg3-lipo as a potential therapeutic agent for suppressing tumor cell growth.

We further evaluated the in vivo efficacy of PTX-Rg3-lipo against human gastric cancer (SNU-16), breast cancer (MDA-MB-231), and esophageal cancer (OE19) and showed that PTX-Rg3-lipo inhibited tumor growth in a dose-dependent manner in these models (Figure 2, B and C). The observed efficacies were similar to the effects produced by administration of the same concentration of the positive control, PTX, but better than those of PTX-lipo.

The patient-derived xenograft (PDX) model was also used to evaluate the in vivo efficacy of PTX-Rg3-lipo. In the PDX model EC043, administration of PTX-Rg3-lipo significantly inhibited tumor growth, resulting in a treatment/control (T/C) ratio of 93% at the end of the experimental treatment at a dose of 30 mg/kg, equivalent to the effect of the same dose of PTX (Figure 2D). Moreover, in all in vivo models, all doses of PTX-Rg3-lipo were well tolerated by the mice, with no decline in average body weight during the experimental treatment. Taken together, the in vitro and in vivo experiments revealed that PTX-Rg3-lipo exerted antitumor effects in xenograft murine models.

### PTX-Rg3-lipo inhibits tumor growth with outstanding efficacy in the immunocompetent murine model.

PTX-Rg3-lipo exhibited potential therapeutic efficacy in xenograft murine models and nontoxicity to the immune system in various large and small animal models, prompting an investigation to determine whether it could further augment and induce immune responses. Given the critical role of the patient’s immune system in tumor progression and prognosis, xenograft models derived from patient cells and tissues that lack essential components of the immune system could not accurately represent the immune response to tumors (28). Therefore, mouse models are critical for assessing efficacy and immune response. To determine whether PTX-Rg3-lipo has the same inhibitory effect on mouse tumors as it does on human tumor cells, MC38 cells were treated with various concentrations of PTX, Nab-PTX, or PTX-Rg3-lipo (equivalent to the indicated PTX concentration). PTX-Rg3-lipo exhibited enhanced cytotoxicity in MC38 cells (Supplemental Figure 5A). Colony formation by MC38 cells was significantly suppressed by PTX-Rg3-lipo treatment (Supplemental Figure 5B). After PTX-Rg3-lipo treatment, the proportion of migrating MC38 cells (assessed using wound healing assays) was significantly reduced compared with that in the PTX and Nab-PTX groups (Supplemental Figure 5C). PTX-Rg3-lipo effectively arrested the progression of MC38 cells to the G_0_ and G_1_ phases of the cell cycle, leading to a reduction in cell proliferation (Supplemental Figure 5D). We then assessed the effect of PTX-Rg3-lipo on the induction of cell death in MC38 cells using flow cytometry. The apoptotic rates of MC38 cells treated with PTX, Nab-PTX, and PTX-Rg3-lipo were 16.44%, 72.35%, and 93.47%, respectively (Supplemental Figure 5E). These results indicate that PTX-Rg3-lipo exhibits inhibitory effects on the proliferation, growth, and migration of tumor cells and induces apoptosis in these cells. To examine the potential selective cytotoxicity of PTX-Rg3-lipo toward tumor cells, MC38 cells and spleen cells from normal mice were subjected to a 48-hour treatment with PTX-Rg3-lipo. PTX-Rg3-lipo promoted apoptosis in MC38 cells but did not affect the proportion of apoptotic cells in the spleens of normal mice (Supplemental Figure 5F). Collectively, these findings suggest that PTX-Rg3-lipo inhibits murine tumor cell proliferation and migration while selectively inducing tumor cell apoptosis.

Furthermore, we established an MC38 tumor model in immunodeficient (NOD *scid* gamma [NSG]) mice (Supplemental Figure 6). DMSO, PTX, PTX-lipo, Rg3-lipo, PTX combined with Rg3-lipo, Nab-PTX, and PTX-Rg3-lipo (equivalent to PTX 10 mg/kg body weight and Rg3 15 mg/kg body weight) were administered i.v. to tumor-bearing mice. The results, similar to those in the human-derived tumor model, demonstrated that PTX-Rg3-lipo had a strong antitumor effect in the immunodeficient mouse model.

**Figure 3:**
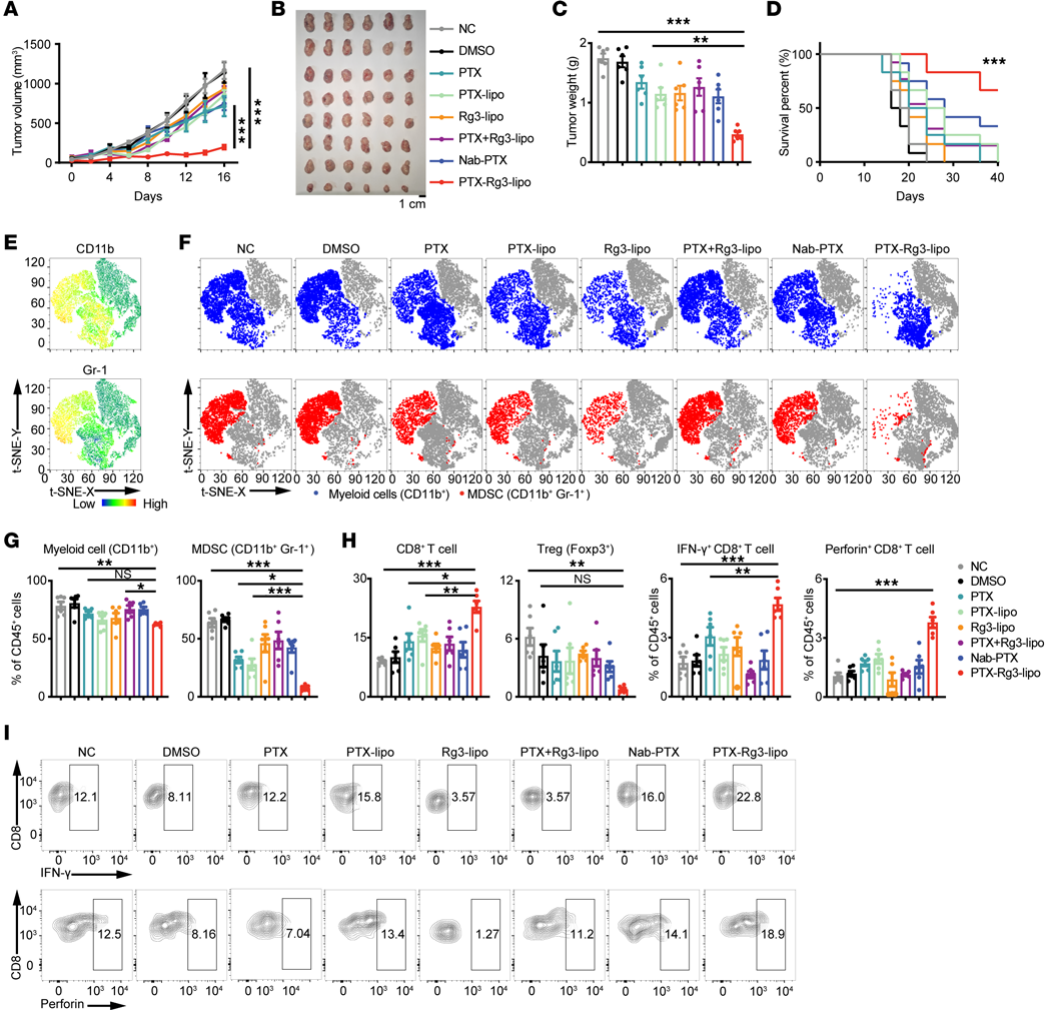
PTX-Rg3-lipo suppresses tumor growth and prolongs survival in the immunocompetent (C57BL/6J) murine model. (**A** and **B**) Tumor growth curves and image of MC38 tumors (*n* = 6). (**C**) Quantification of tumor weights in each group. (**D**) Overall survival rate of the mice in each group (*n* = 12). (**E**) The t-distributed stochastic neighbor embedding (t-SNE) plots display the overall immune landscape and distribution of marker expression in MC38 tumors. (**F** and **G**) Frequencies of myeloid cells (CD11b^+^) and MDSCs (CD11b^+^Gr-1^+^) are shown and quantified as t-SNE plots. (**H**) Frequency of CD8^+^ T cells and regulatory T cells, as well as the production of cytokines such as IFN-γ and Perforin by CD8^+^ T cells, in the tumors from each group. (**I**) Production of cytokines such as IFN-γ and perforin by CD8^+^ T cells in the tumors from each group. Data are shown as mean ± SEM. One-way ANOVA (**C** and **G**–**I**) and 2-way ANOVA (**A**) with post hoc Bonferroni’s test, as well as the log-rank (Mantel-Cox) test (**D**), were used for statistical analysis. *P < 0.05; **P < 0.01; ***P < 0.001.

To explore the effect of PTX-Rg3-lipo on antitumor immune responses, we established an MC38 tumor model in immunocompetent (C57BL/6J) mice and conducted the same intervention. As depicted in Figure 3, A and B, PTX-Rg3-lipo greatly inhibited tumor growth compared with the alternative treatments. The efficacy of Rg3-lipo alone was slightly better than the combination of Rg3-lipo and PTX, possibly because of the induction of toxic side effects by PTX, especially on the immune system. The tumor weights in the PTX-Rg3-lipo group were consistently and significantly lower than those in the PTX and Nab-PTX groups (Figure 3C). The administration of PTX-Rg3-lipo significantly increased the median survival time of mice, extending from 24 to over 40 days (Figure 3D). In the immunocompetent in vivo models, using Rg3-lipo as a drug carrier led to a substantial enhancement in tumor inhibition, from 12.80% (PTX group) to 79.54% (PTX-Rg3-lipo group). Our observations indicated that the enhancement in tumor growth inhibition was more pronounced in immunocompetent mice compared with the immunodeficient mice (Figure 3A and Supplemental Figure 6A). This suggests that the Rg3-lipo system not only enhances the toxic effects of PTX on tumor cells directly, but also exerts an antitumor immune response, leading to an overall improvement in antitumor efficacy.

To comprehensively examine the anticancer properties of PTX-Rg3-lipo in various types of cancer, we also developed a murine model of pancreatic cancer. Our findings suggest that PTX-Rg3-lipo is better than Nab-PTX in terms of its ability to suppress tumor growth (Supplemental Figure 7, A–C) and prolong survival (Supplemental Figure 7D). Furthermore, PTX-Rg3-lipo did not impact the body weight of mice (Supplemental Figure 7E). Collectively, compared with immunodeficient models, PTX-Rg3-lipo demonstrated a stronger therapeutic effect on tumors in immunocompetent in vivo models, underscoring the vital role of immune modulation mechanisms in augmenting the antitumor efficacy of the Rg3-lipo delivery system.

To evaluate the safety of Rg3-lipo, we first examined the in vivo distribution characteristics of Rg3-lipo. Liposomes were fluorescently labeled with DiR and administered i.v. to tumor-bearing mice for 8 hours. The Rg3-lipo fluorescence intensity in tumor tissues was higher than in the control group, verifying that Rg3-lipo effectively promotes drug accumulation at the tumor site (Supplemental Figure 8, A–C). Additionally, we used coumarin-6–labeled liposomes to evaluate drug uptake in single-cell lysates from tissues harvested 8 hours after injection. Rg3-lipo was distributed in various organs, but the tumor uptake of Rg3-lipo was much higher than that of conventional cholesterol liposome (C-lipo), whereas the uptake in the liver, spleen, and colon was similar (Supplemental Figure 8D). These results indicate that Rg3-lipo can effectively enhance tumor targeting. Since PTX-Rg3-lipo is distributed in different tissues, we continuously monitored the body weight of the mice during treatment, and the main organs were collected and stained with hematoxylin and eosin when the tumors reached the treatment endpoint. No significant reduction in body weight (Supplemental Figure 9A) or pathological change in the major organs (hearts, livers, spleens, lungs, and kidneys) was observed (Supplemental Figure 9B). Furthermore, acute and long-term complete blood count, liver, and renal function tests were conducted in the MC38 tumor–bearing mice model. The results demonstrated that neither Rg3-lipo nor PTX-Rg3-lipo affected blood cell ratios, liver functions, or kidney functions, further substantiating the safety of Rg3-lipo as a drug carrier (Supplemental Figure 10).

Additionally, we examined the cellular distribution of Rg3-lipo within the tumor. Rg3-lipo significantly increased liposome uptake by tumor cells and MDSCs in tumor tissue compared with C-lipo, whereas liposome uptake by macrophages, dendritic cells (DCs), T cells, and B cells was unaffected (Supplemental Figure 11). Therefore, Rg3-lipo facilitates drug uptake by cancer cells and MDSCs, prompting investigation of its potential role in immunomodulation. We analyzed the immune profiles of tumor tissues and peripheral blood mononuclear cells using flow cytometry. The results were visualized using a t-distributed stochastic neighbor embedding analysis. First, a significant decrease in the proportion of myeloid cells was observed compared with other immune cells in the PTX-Rg3-lipo treatment group among immune cells (Figure 3, E–G). Consistent with the cellular distribution findings, after treatment with PTX-Rg3-lipo, the number of MDSCs (CD11b^+^Gr-1^+^) within MC38 tumors decreased to approximately one-tenth of that in the control group. Meanwhile, PTX-Rg3-lipo treatment slightly increased the ratio of macrophages and DCs (Supplemental Figure 12). Together, these findings indicate that PTX-Rg3-lipo effectively targets MDSCs to reverse the suppressive tumor microenvironment within MC38 tumors in mice.

Given the crucial role of T cells in eliminating tumors, we investigated the proportion and function of T cells after PTX-Rg3-lipo treatment. As shown in Figure 3H, administration of PTX-Rg3-lipo resulted in a 3-fold enhancement in the infiltration of cytotoxic CD8^+^ T cells, whereas no recruitment of regulatory T cells was observed. Also, PTX-Rg3-lipo significantly increased the production of cytokines, specifically IFN-γ and perforin, by cytotoxic T cells, suggesting that treatment with PTX-Rg3-lipo induces a more robust T cell response in mice (Figure 3I).

To determine the effects of PTX-Rg3-lipo on the systemic immune system, the peripheral blood mononuclear cells were collected and assessed. The proportion of MDSCs in the peripheral circulation consistently and significantly decreased following treatment with PTX-Rg3-lipo (Supplemental Figure 13, A and B). An upregulation of the production of IFN-γ and perforin by CD8^+^ T cells was also observed (Supplemental Figure 13C).

These results depicted an interactive immune milieu in the frequency of MDSCs and T cell expansion following PTX-Rg3-lipo treatment. Therefore, PTX-Rg3-lipo not only directly inhibits tumor growth but also has immunomodulatory effects, particularly on MDSCs and T cells.

### The antitumor effect of PTX-Rg3-lipo partially relies on the blockade of MDSC immunosuppression.

**Figure 4:**
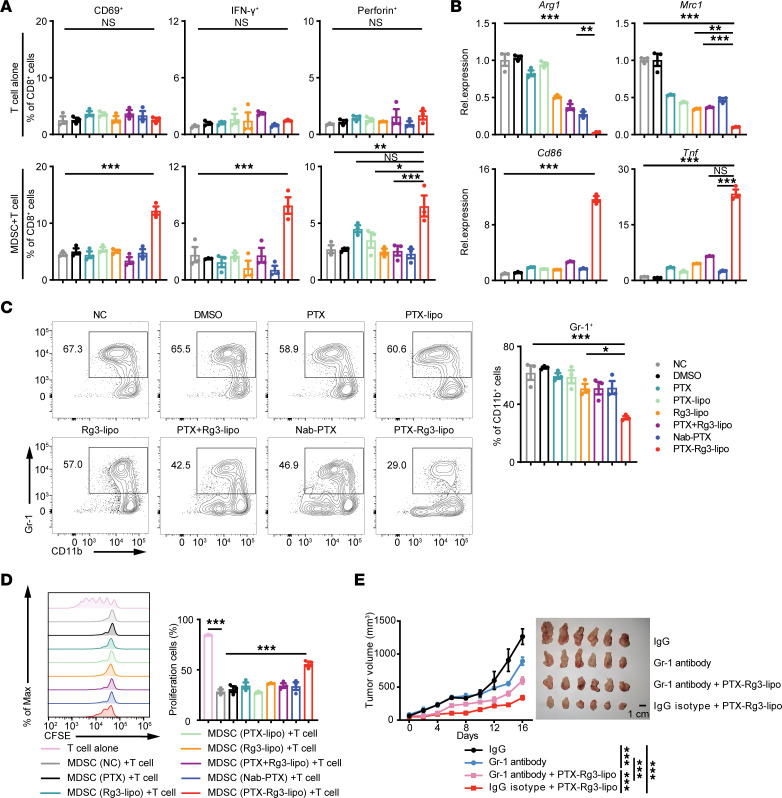
The immunosuppressive function of MDSCs is diminished by PTX-Rg3-lipo. (**A**) T cells isolated from the spleen of C57BL/6J mice using magnetic beads were treated with DMSO, PTX, PTX-lipo, Rg3-lipo, PTX combined with Rg3-lipo, Nab-PTX, and PTX-Rg3-lipo (100 mg/L PTX and/or 150 mg/L Rg3-lipo) for 24 hours. MDSCs were then treated with 20% tumor-conditioned medium of MC38 cells for 24 hours, followed by DMSO, PTX, PTX-lipo, Rg3-lipo, PTX combined with Rg3-lipo, Nab-PTX, and PTX-Rg3-lipo for 24 hours, and added to T cells. Percentages of CD69^+^, IFN-γ^+^, and perforin^+^ were assessed using flow cytometry. (**B**) mRNA levels of *Arg1*, *Mrc1*, *Cd86*, and *Tnf* were quantified using quantitative PCR. (**C**) Percentages of MDSCs are shown as representative flow cytometry plots. (**D**) MDSCs were treated with DMSO, PTX, PTX-lipo, Rg3-lipo, PTX combined with Rg3-lipo, Nab-PTX, or PTX-Rg3-lipo (100 mg/L PTX and/or 150 mg/L Rg3) for 24 hours, and added to CFSE-labeled T cells. T cell proliferation is shown using representative flow cytometry plots and quantification. (**E**) Tumor growth curves and images for each group (*n* = 6). One-way ANOVA (**A**–**D**) and 2-way ANOVA (**E**) with post hoc Bonferroni’s test were used for statistical analysis. *P < 0.05; **P < 0.01; ***P < 0.001.

To further investigate whether PTX-Rg3-lipo directly promotes T cell function, we initially isolated T cells from the spleen of C57BL/6J mice and treated them with PTX-Rg3-lipo. The results indicated that PTX-Rg3-lipo treatment did not directly facilitate T cell activation or enhance cytokine secretion, suggesting an indirect activation of T cells through its effect on MDSCs. Subsequently, we established bone marrow–derived MDSCs for in vitro experiments. Nevertheless, treatment of MDSCs with PTX-Rg3-lipo, followed by coculture with T cells, increased T cell activation and cytokine secretion, indicating that PTX-Rg3-lipo facilitates T cell function by affecting MDSCs (Figure 4A).

Therefore, we investigated the mechanism by which PTX-Rg3-lipo regulates MDSCs. The results demonstrated that it did not cause cell death in MDSCs under standard therapeutic conditions and induced MDSC apoptosis only at exceedingly high doses (Supplemental Figure 14). Notably, there was a switch in functional marker expression of MDSCs: PTX-Rg3-lipo uniquely increased the expression of biomarkers associated with the inflammatory response (*Cd86* and *Tnf*), while diminishing the expression of biomarkers associated with immunosuppression (*Arg1* and *Mrc1*) (Figure 4B). PTX-Rg3-lipo treatment significantly decreased the ratio of MDSCs compared with the other treatment groups, suggesting that PTX-Rg3-lipo treatment effectively inhibited tumor progression by suppressing the accumulation of MDSCs (Figure 4C). These findings indicate that the suppressive function of MDSCs is diminished after PTX-Rg3-lipo treatment.

MDSCs facilitate tumor growth and metastasis by secreting cytokines that exert immunosuppressive effects on CD4^+^ and CD8^+^ T cells (16). Subsequently, to evaluate the impact of PTX-Rg3-lipo–treated MDSCs on T cell proliferation in vitro, we isolated T cells from the spleens of C57BL/6J mice and labeled them with a fluorescent probe (CFSE), followed by coculturing with MDSCs at a 5:1 ratio. T cells alone (stimulated with IL-2 and CD3/CD28 antibodies) were used as positive controls. The MDSCs obtained from the group treated with PTX-Rg3-lipo exhibited a comparatively lower degree of suppression of T cell proliferation than the MDSCs from the remaining groups (Figure 4D). Taken together, these in vitro results suggest that PTX-Rg3-lipo can inhibit MDSC immunosuppression, thereby enhancing the antitumor efficacy of T cells.

To exclude the effect of PTX-Rg3-lipo on other myeloid cells, we treated MC38 cells, MDSCs, macrophages, DCs, and T cells in vitro with C-lipo or Rg3-lipo fluorescently labeled with coumarin-6. Compared with C-lipo, Rg3-lipo increased uptake by tumor cells and MDSCs but had little influence on uptake by macrophages, DCs, and T cells (Supplemental Figure 15, A–C). Additionally, PTX-Rg3-lipo had minimal effect on DC percentage, maturation, antigen-presenting function, and the macrophage M1/M2 ratio (Supplemental Figure 15, D–G). The results indicate that PTX-Rg3-lipo has no notable effect on macrophages and DCs.

To verify whether PTX-Rg3-lipo–mediated modulation of MDSCs contributed to its antitumor effect, we established an MDSC depletion model using a Gr-1 antibody. MDSC knockdown efficiency is shown in Supplemental Figure 16. Depletion of MDSCs inhibited tumor growth. Nevertheless, suppression of MDSCs compromised the immunomodulatory effect of PTX-Rg3-lipo, thereby diminishing its capacity to inhibit tumor growth (Figure 4E). Thus, in vivo experiments demonstrated that MDSC depletion substantially ablated the tumor inhibition mediated by PTX-Rg3-lipo, indicating that the antitumor effect of PTX-Rg3-lipo at least partially relies on the blockade of MDSC immunosuppression.

### PTX-Rg3-lipo–mediated blockade of MDSC immunosuppression is dependent on Glut3 via inhibiting glycolysis.

To further explore the specific mechanism of tumor suppression by PTX-Rg3-lipo, we analyzed the expression of glucose transporters in various cell subpopulations in colorectal cancer (CRC) (29). Studies have revealed that tumor targeting of PTX-Rg3-lipo is primarily achieved in tumor cells through a GLUT1-mediated mechanism (24, 25). To confirm this finding, MC38 cells were treated with fluorescently labeled C-lipo and Rg3-lipo. Microscopy and flow cytometry analysis revealed that Rg3-lipo significantly enhanced tumor cell uptake compared with C-lipo, and this effect was suppressed by Glut1 knockdown (Supplemental Figure 17, A–D). Similar results were observed in human-derived SW620 cells using both microscopy and flow cytometry (Supplemental Figure 17E). These findings confirmed that Rg3-lipo increased Glut1-mediated tumor cell uptake.

**Figure 5:**
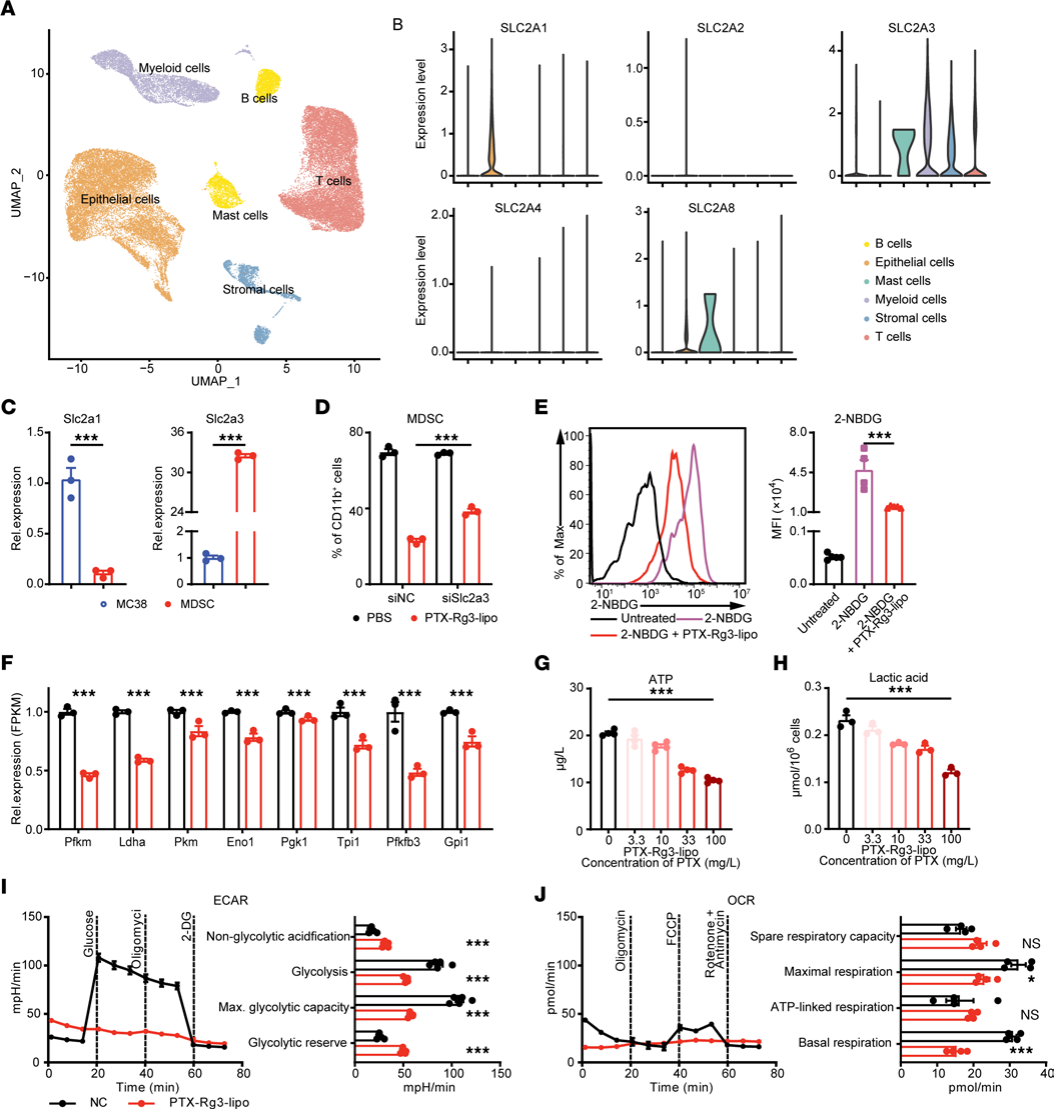
PTX-Rg3-lipo inhibits MDSC glycolysis via Glut3. (**A** and **B**) Uniform manifold approximation and projection (UMAP) clustering and glucose transporter isoform expression levels determined using scRNA-Seq data from patients with CRC. (**C**) mRNA levels of the Glut1-encoding gene *Slc2a1* and the Glut3-encoding gene *Slc2a3* in MC38 cell lines and bone marrow MDSCs. (**D**) MDSCs were pretreated with PBS or PTX-Rg3-lipo (100 mg/L PTX) and electroporated with control or Slc2a3-siRNA. The ratio of MDSCs was determined using flow cytometry. (**E**) Changes in glucose uptake following PTX-Rg3-lipo treatment using 2-NBDG. (**F**) Relative expression levels (fragments per kilobase of transcript per million mapped reads; FPKM) of glycolytic genes following PTX-Rg3-lipo treatment. (**G** and **H**) Quantification of ATP and lactic acid production. (**I** and **J**) Seahorse analysis of MDSCs after PTX-Rg3-lipo treatment. Data are shown as mean ± SEM. One-way ANOVA with post hoc Bonferroni’s test (**D**, **E**, **G**, and **H**) and unpaired 2-tailed *t*-test (**C**, **F**, **I**, and **J**) were used for statistical analysis. *P < 0.05; ***P < 0.001.

Subsequently, to explore the mechanism by which MDSCs utilize PTX-Rg3-lipo, publicly available single-cell RNA sequencing (scRNA-Seq) data from patients with CRC were used to determine the expression levels of the glucose transporter isoforms GLUT1, GLUT2, GLUT3, and GLUT4 in various compartments of the tumor microenvironment. The Glut1-encoding gene *Slc2a1* was predominantly expressed in tumor cells, whereas the Glut3-encoding gene *Slc2a3* was expressed weakly in tumor cells but strongly in immune cells, particularly myeloid cells. This suggests that GLUT3 is more essential than GLUT1 for the reduction in MDSCs (Figure 5, A and B). Additionally, analysis of scRNA-Seq data from colon cancer and its paracancerous tissue revealed a notable upregulation of Glut3 expression in tumor-local myeloid cells compared with paracancerous myeloid cells (Supplemental Figure 18A). Subsequently, the analysis of Glut3 expression levels across distinct myeloid cell subpopulations revealed that Glut3 expression was higher in MDSCs than in DCs, while the expression in macrophages and monocytes was extremely low and negligible (Supplemental Figure 18B). Quantification of *Slc2a1* and *Slc2a3* expression was performed in MC38 cells and MDSCs using quantitative PCR, which showed a consistent pattern of higher expression of *Slc2a1* in MC38 and *Slc2a3* in MDSCs (Figure 5C). Therefore, we postulate that Glut3 is responsible for the absorption of PTX-Rg3-lipo by MDSCs.

To verify this hypothesis, expression of the Glut3-encoding gene *Slc2a3* in MDSCs was knocked down using small interfering RNA (siRNA). The knockdown efficacy of *Slc2a3* was determined using quantitative PCR (Supplemental Figure 19A). The ratio of MDSCs decreased by PTX-Rg3-lipo treatment was partially restored by *Slc2a3* knockdown (Figure 5D). Following the knockdown of Glut3, neither PBS nor PTX-Rg3-lipo resulted in significant cell death in MDSCs (Supplemental Figure 19B). Confocal microscope observation showed that the uptake of Rg3-lipo was significantly increased in MDSCs compared with C-lipo, and the knockdown of Glut3 resulted in a significant decrease in Rg3-lipo uptake, confirming the uptake of PTX-Rg3-lipo by MDSCs via Glut3 (Supplemental Figure 19C). Furthermore, the coculture experiment of T cells and MDSCs also demonstrated that depletion of Glut3 in MDSCs maintained the immunosuppressive function of MDSCs while concurrently decreasing the production of IFN-γ and perforin in T cells (Supplemental Figure 19D). These findings suggest that Glut3 mediates the effect of PTX-Rg3-lipo on MDSCs, which is essential for alleviating MDSC-mediated antitumor immunosuppression.

The Warburg effect suggests that cancer cells preferentially utilize glucose as their primary energy source. Recent studies have suggested glycolysis as a vital energy source for MDSCs, thereby affecting their function within the tumor microenvironment (30–33). To elucidate the modulatory effects of PTX-Rg3-lipo on glucose metabolism in MDSCs, we used the 2-NBDG fluorescent probe. A substantial reduction in glucose uptake was observed in the MDSCs after PTX-Rg3-lipo treatment (Figure 5E), along with a decrease in the expression of glycolysis-related genes (Figure 5F). This phenomenon was paralleled by a significant decrease in ATP production within these cells (Figure 5G), accompanied by a reduction in lactate production (Figure 5H). An in-depth metabolic analysis using the Seahorse Analyzer revealed that PTX-Rg3-lipo markedly reduced glycolysis levels in MDSCs (Figure 5I), indicating that PTX-Rg3-lipo mediates the metabolic reprogramming of MDSCs. Despite these changes, we observed no significant alterations in the level of oxidative phosphorylation in MDSCs (Figure 5J). Further analysis was conducted to assess the metabolic effects of PTX and Rg3-lipo on MDSCs (Supplemental Figure 20). The results demonstrated that PTX-Rg3-lipo markedly reduced glucose uptake, ATP and lactate production, and glycolysis levels in MDSCs compared with the control, PTX-lipo, and Rg3-lipo, exhibiting the most pronounced glycolysis inhibition effect. These results indicate that PTX and Rg3 are jointly responsible for the regulation of metabolism in MDSCs. Collectively, these results suggest that the blockage of MDSC immunosuppression by PTX-Rg3-lipo is dependent on Glut3 via glycolysis inhibition.

### Inhibition of glycolysis in MDSCs by PTX-Rg3-lipo leads to the downregulation of the c-Maf/Mafb pathway, resulting in blockade of MDSC immunosuppression.

**Figure 6:**
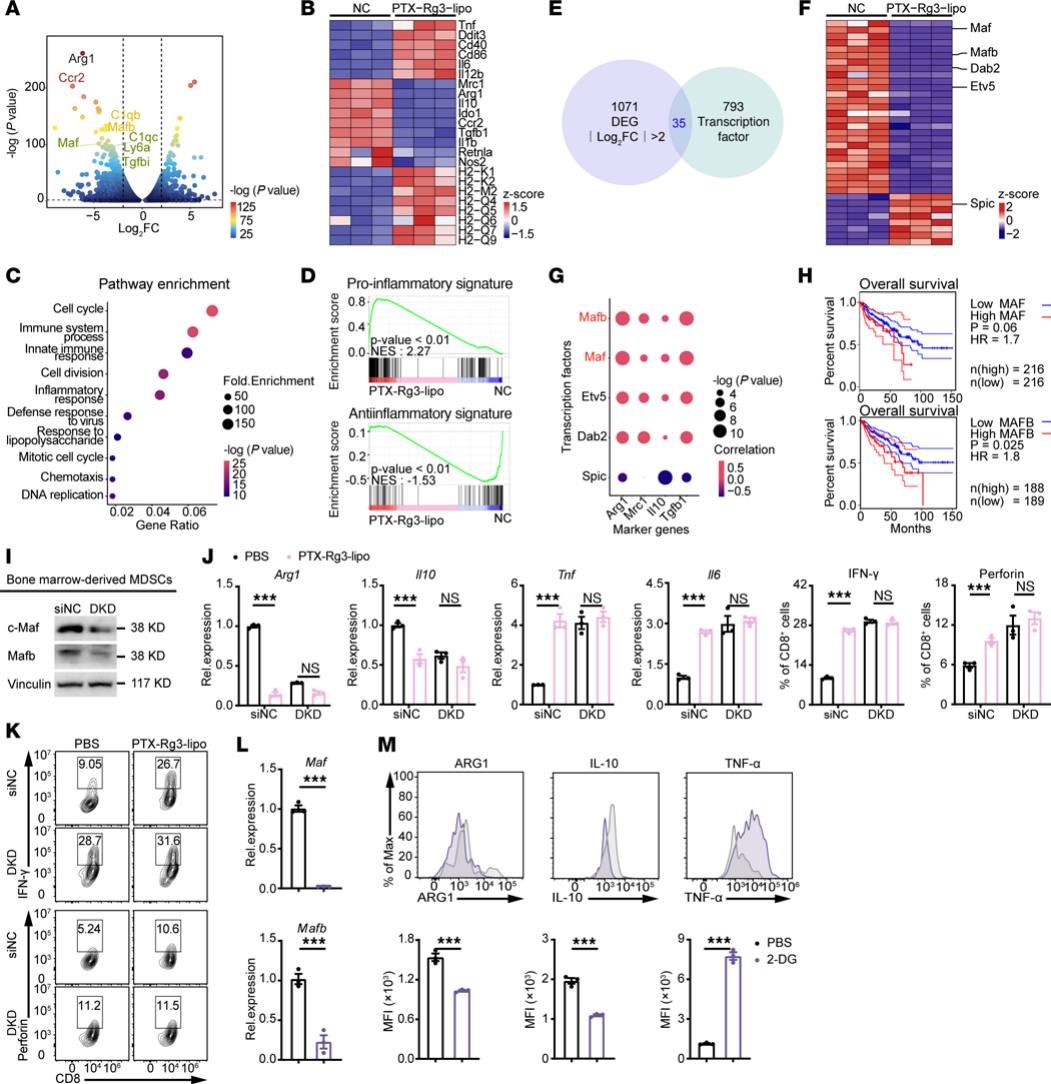
PTX-Rg3-lipo blockades MDSCs’ immunosuppressive function mediated by c-Maf/Mafb. (**A** and **B**) Volcano plot and heatmap of PTX-Rg3-lipo interference of MDSC differential genes. (**C**) KEGG enrichment analysis. (**D**) GSEA of proinflammatory and antiinflammatory signature changes. (**E**) Differentially expressed genes (|log_2_FC| > 2) and mouse transcription factors were used for intersection. (**F**) Heatmap of transcription factors with |log_2_FC| > 2. (**G**) Correlation analysis of the most significantly changed transcription factors and key functional molecules in MDSCs. (**H**) Survival analysis of MAF and MAFB expression in patients with CRC. (**I**) The efficiency of c-Maf and Mafb in MDSC knockdown experiments. DKD, double knockdown. (**J** and **K**) Changes in MDSC biomarkers and T cell function after DKD and PTX-Rg3-lipo treatment. (**L**) Quantification of *c-Maf* and *Mafb* expression following 2-deoxy-d-glucose (2-DG) treatment. (**M**) Quantification of MDSC cytokine secretion following 2-DG treatment. Data are shown as mean ± SEM. The log-rank (Mantel-Cox) test (**H**), 1-way ANOVA with post hoc Bonferroni’s test (**J**), and unpaired 2-tailed *t* test (**L** and **M**) were used for statistical analysis. ***P < 0.001.

To elucidate the molecular mechanism by which PTX-Rg3-lipo–mediated inhibition of glycolysis in MDSCs blocks MDSC immunosuppression, we conducted RNA-Seq on PTX-Rg3-lipo– or PBS-treated MDSCs. The findings verified a notable reduction in the transcription levels of antiinflammatory functional molecules, including *Arg1*, *Il10*, and *Ido1*, whereas proinflammatory molecules such as *Tnf*, *Il6*, and *Il12b*, as well as MHC class II molecules, exhibited a significant increase in expression (Figure 6, A and B) (34). Kyoto Encyclopedia of Genes and Genomes (KEGG) pathway enrichment analysis revealed that the differentially expressed genes following PTX-Rg3-lipo treatment were primarily enriched in pathways associated with immune response activation and cell cycle regulation (Figure 6C). Gene set enrichment analysis (GSEA) revealed substantial changes in the expression patterns of marker genes associated with pro- and antiinflammatory phenotypes (Figure 6D) (35–38). Furthermore, we validated the notable alterations in the expression profiles of marker genes linked to the glycolysis phenotype through GSEA (Supplemental Figure 21). These results suggest that PTX-Rg3-lipo promotes MDSCs toward the tumoricidal phenotype.

To further clarify the targets of PTX-Rg3-lipo in MDSCs, we intersected all differentially expressed genes with a collection of mouse transcription factors and identified 35 transcription factors with notable alterations (Figure 6, E and F) (39). Correlation analysis revealed that *c-Maf* and *Mafb* exhibited the most notable changes, and they displayed a robust correlation with crucial molecules implicated in the immunosuppressive function of MDSCs (Figure 6G). *c-Maf* and *Mafb* are crucial transcription factors that regulate the differentiation, development, self-renewal, and function of myeloid cells (40–42). Survival analysis in patients with CRC from The Cancer Genome Atlas database indicated that elevated transcriptional levels of both MAF and MAFB were linked to poor prognosis in patients with CRC, verifying our findings (Figure 6H).

We used siRNA to induce the knockdown of both c-Maf and Mafb transcription factors (DKD) in MDSCs and subsequently verified the effectiveness of the knockdown (Figure 6I). Examination of MDSC function following knockout demonstrated that DKD significantly reduced the expression of antiinflammatory molecules (such as *Arg1* and *Il10*) in MDSCs, accompanied by an increase in the expression of proinflammatory molecules (such as *Tnf* and *Il6*) (Figure 6J). Notably, PTX-Rg3-lipo administration after knockout did not further reduce or enhance the expression of these effector molecules. The coculture experiment showed that the DKD restored the cytokine secretion function of T cells; however, the enhancement effect of PTX-Rg3-lipo was no longer discernible (Figure 6K). These results indicate that PTX-Rg3-lipo blocks the immunosuppressive function of MDSCs via modulation of c-Maf and Mafb.

To elucidate the precise pathway by which PTX-Rg3-lipo regulates MDSC glycolysis and immunosuppressive function via c-Maf/Mafb, we treated MDSCs with 2-deoxy-d-glucose and showed that the inhibition of the glycolysis in MDSCs downregulated *c-Maf* and *Mafb*, subsequently decreasing the protein expression of ARG1 and IL-10 while increasing that of TNF-α (Figure 6, L and M). We conclude that the inhibition of glycolysis in MDSCs by PTX-Rg3-lipo via Glut3 downregulates the c-Maf/Mafb pathway, thereby blocking the immunosuppressive function of MDSCs and consequently promoting an enhanced antitumor immune response.

## Discussion

Liposomal drug delivery systems can address the lack of targeted specificity, substantial toxic side effects, and therapeutic resistance in traditional cytotoxic drugs; however, they face several obstacles, including relative instability, safety limitations, potential immunotoxicity, and the complexity of the tumor microenvironment, which impede their clinical application.

Therefore, owing to the favorable stability and abundance of glycosyl moieties in Rg3, we developed a drug delivery system, Rg3-lipo, by replacing cholesterol with Rg3. In our study, Rg3-lipo demonstrated exceptional stability and bioavailability and proved to be nontoxic in various physiological systems, including hematology, respiratory, cardiovascular, hepatic, and renal functions, with particular emphasis on its safety in the immune system. Additionally, the Rg3-lipo drug delivery system demonstrated a notable enhancement in antitumor efficacy in not only human-derived tumor models but also immunocompetent mice, prompting further investigation of its immunomodulatory mechanisms. The glycosyl moieties of Rg3-lipo selectively targeted Glut3 on MDSCs, effectively inhibiting glycolysis and downregulating the expression of the transcription factors c-Maf and Mafb. This pivotal mechanism weakened the MDSC-mediated functional suppression of effector T cells, thereby augmenting the immune response against tumors. In summary, our research used various large and small animal models, including nonhuman primates, that closely resembled clinical conditions, validating the exceptional safety profile, potent therapeutic effectiveness, and immunomodulatory effects and mechanisms of Rg3-lipo, thereby facilitating its translation into clinical use.

Notably, the incorporation of glycosyl moieties into the liposome structure confers several advantages to Rg3-lipo, such as increased drug loading capacity and improvement of targeted delivery (26). The distinctive configuration of this structure renders it less vulnerable to immune elimination, thereby extending its duration in circulation and enhancing its capacity for the precise administration of therapeutic agents (22). Rg3’s hydrophobic core is inserted into the bilayer membrane of the liposome, while its hydrophilic glycosyl moieties are exposed on the surface, resulting in the acquisition of biomimetic characteristics by Rg3-lipo, which enables the active targeting of specific tumor cells and demonstrates outstanding targeted performance (25, 43). This targeting strategy minimizes nonspecific distribution, mitigates off-target effects, and efficiently delivers drugs to tumor sites (25). Although previous research has substantiated certain benefits of Rg3, there are similarities to conventional liposomes in certain aspects. Both Rg3-lipo and conventional liposomes enhance drug delivery efficiency and reduce toxicity, providing a valuable platform for drug delivery systems (22). Rg3-lipo is a promising candidate for a targeted drug delivery system owing to its glycosyl-based structure, which confers several benefits. Optimization of Rg3-lipo will contribute to its realization as a fully functional, advanced drug delivery system.

The immune system is deeply involved in tumor progression and prognosis. However, PTX not only kills tumor cells directly but also impairs the immune system and increases the risk of infection, thereby limiting its efficacy. Although PTX-lipo reduces adverse effects, its complex structure causes a slow drug release, preventing the timely achievement of the required local drug concentration for effective tumor suppression (7, 44). More importantly, previous approaches have ignored PTX’s immune system safety and immunomodulatory effects, leading to insufficient efficacy in clinical trials and in vitro studies (45–48). Similar results were observed in our study. Consequently, the current investigation centered on assessing the safety of PTX-Rg3-lipo for the immune system and the potential modulatory effects of PTX-Rg3-lipo on the tumor immunosuppressive microenvironment. The tumor immune microenvironment plays a critical role in cancer treatment, particularly in regulating MDSCs (49, 50). Substantial accumulation of MDSCs within the tumor microenvironment inhibits the activation and function of T cells, thus impeding the immune system from targeting cancer cells (51). Therefore, overcoming MDSC-induced immunosuppression is crucial to enhance the effectiveness of cancer therapies (52). Our study has validated the crucial role of immune modulation mechanisms in enhancing the antitumor effectiveness of the Rg3-lipo delivery system, indicating that Rg3-lipo exhibits a unique potential in regulating the immunosuppressive microenvironment. Rg3-lipo suppressed the accumulation of MDSCs and their immunosuppressive functions, thereby promoting antitumor immune responses. Meanwhile, we investigated the potential immunotoxic effects of PTX-Rg3-lipo and found that PTX-Rg3-lipo demonstrated an excellent safety profile in this regard. These discoveries provide insights for future research and will help to facilitate the clinical use of Rg3-lipo in tumor immunotherapy.

Cancer cells consume two-thirds of glucose while myeloid cells consume one-third in the tumor microenvironment (53–55). Glut1 was previously considered the primary transporter of the Rg3-lipo drug delivery system in cancer cells (24, 25). Consistent with prior results, we found that tumor cells mostly expressed the Glut1-encoding gene *Slc2a1*, whereas myeloid cells predominantly expressed *Slc2a3*. Consequently, we hypothesized that Glut3 may be the primary transporter for the Rg3-lipo drug delivery system in myeloid cells. Our findings confirm that Glut3 is required for the blockade of MDSCs induced by PTX-Rg3-lipo. The glycosyl moiety–enriched Rg3-lipo drug delivery system selectively inhibited tumor cell proliferation and accumulation of MDSCs by targeting Glut1 and Glut3, respectively, promoting an enhanced antitumor immune response. Considering Rg3-lipo’s efficacy as a chemotherapeutic drug delivery system, along with its safety profile, antitumor effects, and versatile targeting capabilities, an in-depth understanding of the targeting mechanism and immunomodulatory effects remains essential to facilitate its application in various cancer subtypes and the development of chemotherapeutic drugs to improve the effect of tumor immunotherapy.

Despite thorough preclinical investigations, substantial clinical validation is imperative to bridge the gap from Rg3-lipo’s preclinical potential to clinical application. In addition, the different release kinetics of PTX and Rg3, influenced by their chemical structures, hydrophilic-lipophilic properties, and interactions with the liposomal membrane, may affect their stability and release rates in a clinical setting. Furthermore, the insufficient exploration of potential long-term and unforeseen adverse effects emphasizes the need for ongoing monitoring to ensure its sustained viability. These constraints underscore the necessity for additional clinical scrutiny and vigilance before Rg3-lipo’s integration into cancer therapy is contemplated.

This study thoroughly examined the safety, effectiveness, and immunomodulatory effects and mechanisms of Rg3-lipo using preclinical translational models that encompassed nonhuman primates. Our findings not only facilitate the translation of Rg3-lipo for clinical applications but also promise to improve cancer treatment options and provide valuable insights for other drug delivery systems.

## Methods

Further information can be found in Supplemental Methods.

### Sex as a biological variable.

Our study examined male and female animals, and similar findings are reported for both sexes.

### Reagents.

The liposomal formulation of ginsenoside Rg3 is composed of egg yolk phospholipids and Rg3(S) at a precise weight ratio of 5:2. To begin the process, the lipids are dissolved in a mixed solvent of chloroform and ethanol in a 1:1 ratio. This solution is then subjected to rotary evaporation at 50°C, which facilitates the formation of a thin lipid film on the surface of the evaporation flask. After the evaporation process, the lipid film is carefully hydrated under a nitrogen atmosphere to prevent oxidation. This is achieved by the addition of 1 mL of a 5% glucose solution to the film, and the mixture is maintained at 50°C for 30 minutes to ensure complete hydration. The resulting hydrated lipid film transforms into a liposomal suspension. To achieve a uniform and stable liposomal suspension, the mixture undergoes sonication using a probe sonicator (VCX750, Sonics & Materials Inc.). The sonication process is meticulously controlled, involving 30 cycles of 5 seconds on and 5 seconds off at an energy output of 300 W. This procedure helps to reduce the size of the liposomes and achieve a homogeneous suspension.

The liposomal suspension was further diluted to an appropriate concentration, and its particle size, polydispersity index, and ζ potential were determined using the dynamic light scattering technique. To observe the morphology of the ginsenoside-loaded liposomes, the liposomal suspension was stained with 2% uranyl acetate for 30 minutes, and a droplet was placed on the front side of a copper grid. After standing in the dark for more than 2 hours, excess water was removed using filter paper, and the morphology was examined using transmission electron microscopy. Normal cholesterol liposomes were used as controls and subjected to the same treatment. The size distribution of Rg3-lipo and PTX-Rg3-lipo was examined with dynamic light scattering (Supplemental Figure 22, A–C).

According to our granted patent, we tested the tumor inhibitory effects of PTX-Rg3-lipo, PTX-lipo, and a blank control group with different ratios of PTX and Rg3 prescriptions (specifically, 1:0.5, 1:1, 1:1.5, and 1:4) to achieve optimal antitumor effects by establishing a nude mouse xenograft tumor model of human gastric carcinoma (56). In each formulation, the PTX content was maintained at 15 mg/kg. The experimental results demonstrated that the 4 distinct ratios of PTX-Rg3-lipo exhibited a notable inhibition of the growth of transplanted tumors in nude mice with human gastric carcinoma, with their antitumor effects being better than those of PTX-lipo alone. The tumor inhibition rates reached 12%, 40%, 95%, and 37%, respectively, at day 28. Among these, the highest tumor inhibition rate was observed when the ratio of PTX to Rg3 was 1:1.5, which demonstrated the strongest antitumor effect. Based on these findings, the optimal ratio of PTX to Rg3 was identified as 1:1.5, which was used to prepare liposomes to achieve the most efficacious therapeutic outcome.

Examination of the impact factor test revealed that the particle size of PTX-Rg3-lipo remained essentially unchanged when it was placed in a high-temperature (30°C) and high-humidity (25°C, relative humidity 90%) environment for 30 days and under strong light irradiation (20°C, 5,000 lx, 83 μW/cm^2^) for 11 days (Supplemental Figure 22D). Particle size also remained essentially unchanged in the accelerated stability test (25°C ± 2°C, 60% ± 5% for 6 months) and the long-term stability test (5°C ± 3°C for 24 months) (Supplemental Figure 22, E and F). In the compounding stability test, PTX-Rg3-lipo solution was prepared with 5% dextrose solution according to PTX concentrations of 1.0 mg/mL and 2.4 mg/mL and stored at room temperature protected from light. The results showed that the particle size was unchanged at PTX concentrations of 1.0 mg/mL and 2.4 mg/mL after the product was formulated with 5% dextrose solution and placed for 8 hours, indicating that the product was stable with 5% dextrose solution for 8 hours (Supplemental Figure 22G). Drug release kinetics was evaluated using a 1N sodium salicylate (pH 7.4) PBS solution as the release medium and a 12 to 14 kDa regenerated cellulose dialysis membrane, as shown in Supplemental Figure 22H. The membrane was immersed in purified water at 100°C for 10 minutes. PTX-Rg3-lipo formulation was placed within the dialysis bag and fully immersed in the release medium. The system was maintained at 37°C with constant magnetic stirring at 0.167*g*. Samples were taken at specified time intervals to measure drug concentration for analysis of release profiles. The release kinetics of PTX and Rg3 was assessed by fitting of the time-dependent concentration data using Origin’s Nonlinear Curve Fit tool, and the half-life (*t*_1/2_) was calculated from the resulting parameters (7, 57, 58). In conclusion, PTX-Rg3-lipo showed good stability under various environmental conditions.

To prepare coumarin-6– and DiR-loaded Rg3-lipo and C-lipo with the same membrane composition, we weighed coumarin-6 powder and DiR powder in a dark environment, dissolved the powders in DMSO to prepare stock solutions at a concentration of 2 mg/mL each, weighed the required membrane materials, added 10 μL of coumarin-6 solution or 10 μL of DiR solution, and dissolved the mixture in a 1:1 ratio of anhydrous ethanol and chloroform.

PTX, Rg3-lipo, and PTX-Rg3-lipo were provided by Xiamen Ginposome Pharmaceutical Co. Ltd. Nab-PTX was purchased from Celgene Corp. PTX-lipo was purchased from Nanjing Luye Pharmaceutical Corp. Rg3-lipo and PTX-Rg3-lipo were dissolved in 5% glucose solution. PTX was dissolved in a 5% glucose solution containing 1% DMSO. Nab-PTX was dissolved in normal saline.

### Animals and cell lines.

SD rats (age 6 weeks, average weight 210 g, equal numbers of males and females) were purchased from Beijing Vital River Laboratory Animal Technology Co. Ltd., and beagle dogs (age 14 months, average weight 10.3 kg, equal numbers of males and females) were purchased from Beijing Marshall Biotechnology Co. Ltd. C57BL/6J mice (age 6 weeks, average weight 18 g, female) were purchased from Shanghai SLAC Laboratory Animal Co. Ltd. BALB/c nude mice (age 4 weeks, average weight 18 g, female) were purchased from Shanghai Institute of Materia Medica. All the mice were maintained in a specific pathogen–free animal facility of Fudan University in laboratory conditions (23°C, 50% humidity, 12-hour light/12-hour dark).

Human, beagle dog, SD rat, and ICR mouse exogenous plasma samples were obtained from BioIVT, while cynomolgus monkey plasma was obtained from Bioduro to study Rg3 metabolites in exogenous plasma incubation systems. Human, beagle dog, SD rat, and ICR mouse liver microsomes were obtained from Xeno Tech, and cynomolgus monkey liver microsomes were obtained from Corning Gentest to study the metabolic stability of Rg3 in liver microsomes.

The Caco2, SW480, SW620, and HT29 cells were purchased from the Cell Bank of the Type Culture Collection of the Chinese Academy of Sciences (Shanghai, China). The MC38 cells were purchased from Kerafast. Panc02 and SNU-16 were purchased from American Type Culture Collection. OE19, OE33, and EC109 were purchased from Nanjing Cobioer Biotechnology Co. Ltd. MKN-45, MKN-74, and SH-10-TC were purchased from Riken Biological Resources Center. SNU-1 and SNU-5 were purchased from Korean Cell Line Bank. CAL-51 was purchased from Deutsche Sammlung von Mikroorganismen und Zellkulturen. MCF-7 was purchased from Shanghai Institute of Nutrition and Health. BT-549 was purchased from Shanghai Institute of Cell Biology. All cell lines were cultured in DMEM (Gibco) supplemented with 10% fetal bovine serum (FBS; Gibco) and 50 IU/mL penicillin/streptomycin (Gibco) at 37°C, 5% CO_2_.

Following trypsin digestion of logarithmic-phase tumor cells (SNU-16, OE19, and MDA-MB-231), the reaction was terminated, and the cell suspension was centrifuged at 500*g* for 5 minutes to remove the supernatant. The cells suspended in 100 μL of PBS were inoculated subcutaneously into the right axilla of BALB/c nude mice at a density of 5 × 10^6^ cells per mouse. The transplanted tumor tissue was cut into 1.5 mm^3^ sections and inoculated subcutaneously into the right axilla of nude mice under sterile conditions.

The human esophageal carcinoma EC043 model derived from a tumor patient was used as the P9 generation transplanted tumor model. The tumor tissue in the vigorous growth stage was cut into approximately 1.5 mm^3^ sections and inoculated subcutaneously into the right axilla of nude mice under sterile conditions. The diameter of the transplanted tumors in nude mice was measured using a Vernier caliper, and the animals were randomly divided after the average volume of the tumor grew to approximately 100–120 mm^3^. Each group was given the corresponding drugs via tail vein injection once a week for 3 weeks. The diameter of the transplanted tumors was measured twice per week, and the body weight was measured throughout the experiment.

The effect of a single i.v. injection of PTX-Rg3-lipo through the tail vein on the central nervous system was evaluated in SD rats. The modified Irwin test was conducted at multiple time points before and after administration, and body temperature was measured.

An unanesthetized, unrestrained SD rat model was used, in which a single i.v. injection of the appropriate medication was administered through the tail vein to assess the impact on respiratory parameters. A whole-body plethysmograph system was used to record and analyze the respiratory rate, tidal volume, and minute ventilation at several time points before and after administration.

An unanesthetized, unrestrained beagle dog model was used, in which a single i.v. injection of the appropriate medication was administered through the limb vein to evaluate the potential cardiovascular effects. Telemetry devices were implanted for blood pressure and electrocardiography measurements. Cardiovascular data collected via a DSI Physiological Data Acquisition System (Data Sciences International) were used to analyze various metrics, including mean arterial pressure, diastolic blood pressure, systolic blood pressure, heart rate, RR interval, and QRS duration.

### Generation of myeloid-derived suppressor cells from murine bone marrow cells.

Mouse bone marrow–derived myeloid-derived suppressor cells (MDSCs) were differentiated in vitro as previously described (59–61). Briefly, bone marrow cells were flushed from the femora of mice using PBS. The suspension was filtered through a 70 μm cell strainer (Corning), transferred to a 50 mL centrifuge tube, and centrifuged for 5 minutes at 500*g*. Red blood cells were lysed using ACK lysis buffer for 3 minutes at room temperature. Cells were washed with PBS and cultured in RPMI 1640 medium supplemented with 10% heat-inactivated FBS, GM-CSF (40 ng/mL), and IL-6 (10 ng/mL). MDSCs were harvested on day 5 and seeded in 24-well plates, followed by induction with tumor-conditioned media from MC38 cells for 24 hours.

To ensure the scientific validity of the method, we scored the similarity of the transcriptomic phenotype of bone marrow–derived MDSCs with various subpopulations of tumor-local myeloid cells by utilizing the analysis of scRNA-Seq data from a public database (Gene Expression Omnibus [GEO] GSE244797) (Supplemental Figure 23, A and B). scRNA-Seq data from colorectal cancer patients were processed using the Seurat framework, including normalization, principal component analysis, identification of nearest neighbors, clustering, and dimensionality reduction via uniform manifold approximation and projection (UMAP). Cell populations were identified by reported markers: macrophages (F4/80, Sirpa, Mrc1), DCs (CD11c, MHC class II), MDSCs (Ly6g, S100A8, S100A9, Ptgs2, Ccl2, Arg1, iNOS), and monocytes (Ly6c, Ccr2, lacking other lineage markers and suppressive genes) (62). The average gene expression profile for each myeloid cell subpopulation was calculated to represent the overall transcriptional signature of that subpopulation. Subsequently, the RNA-sequencing results of bone marrow–derived MDSCs in the current study (PBS-treated group in Figure 6A) were compared with tumor-local MDSCs, monocytes, DCs, proliferating cells, and macrophages in the colorectal cancer single-cell data set using Seurat’s AddModuleScore function to quantify the transcriptional similarity, followed by Wilcoxon’s test for statistical significance. The results of the analysis demonstrated that the bone marrow–derived MDSCs in the current study exhibited the highest similarity score to tumor-local MDSCs, much higher than those of macrophages and DCs, suggesting that this model can effectively simulate tumor-local MDSCs. Furthermore, public databases were used to facilitate a comparative analysis of the markers between bone marrow–derived MDSCs and bone marrow–derived macrophages (BMDMs) (GSE18704 and GSE242723). The results demonstrated notable discrepancies in the expression of several pivotal markers. In particular, bone marrow–derived MDSCs in the current research (PBS-treated group in Figure 6A) and the reference data set demonstrated markedly elevated levels of *Ly6c* and *Ly6g* in comparison with BMDMs. Moreover, bone marrow–derived MDSCs in the current research and the reference data set exhibited elevated expression of *S100A8* and *S100A9*, markers intimately linked to the establishment of an immunosuppressive milieu. These molecular differences suggest that the bone marrow–derived MDSCs in the current research align with the reference bone marrow–derived MDSCs but differ from BMDMs (Supplemental Figure 23C).

### Statistics.

Statistical analyses were conducted using Prism 8.3.0s software (GraphPad Software Inc.). Data are expressed as means ± SEM, and normality was investigated using the Shapiro-Wilk normality test. Statistical differences between 2 groups were determined by 2-tailed Student’s *t* tests. Differences among multiple groups were evaluated using post hoc testing of analysis of variance (1-way or 2-way ANOVA) with Tukey’s honestly significant difference test. Significant differences between or among groups are indicated by *P* < 0.05.

### Study approval.

All animal experiments were performed according to the guidelines of the Administration of Laboratory Animals issued by the Science and Technology Commission of China and approved by the Animal Experimental Ethical Committee of Fudan University, Shanghai, China (202308016Z). At the experimental endpoint, euthanasia of the animals was conducted using CO_2_ asphyxiation.

### Data availability.

All data are available in this article. All materials are available upon reasonable request. All the data used to generate graphs are provided in the Supporting Data Values file. Sequencing data in the study were deposited into Genome Sequence Archive (PRJCA020609).

## Author contributions

JL and FL generated the ideas, designed experiments, and wrote the manuscript. YS, BZ, WZ, and DW performed most experiments, organized all figures, and wrote the manuscript. LC, HS, XP, SM, BJ, and HC analyzed the data and revised the manuscript. HZ provided important materials or reagents. All authors critically reviewed and approved the manuscript.

## Supplementary Material

Supplemental data

Unedited blot and gel images

Supporting data values

## Version 1 11/15/2024

Electronic publication
