# Supplementary material for: Rg3-lipo biomimetic delivery of paclitaxel enhances targeting of tumors and myeloid-derived suppressor cells
Source: J Clin Invest. 2024 Nov 15;134(22):e178617. doi: 10.1172/JCI178617 (PMC11563678; doi:10.1172/JCI178617)
Supplement: Supplemental data [file jci-134-178617-s038.pdf]

Rg3-lipo biomimetic delivery of paclitaxel enhances targeting of tumors and myeloid-derived suppressor cells

Authors: Yuru Shen[1]†, Bin Zhong[1,2]†, Wanwei Zheng[1]†, Dan Wang[3†], Lin Chen[1], Huan Song[1], Xuanxuan Pan[1], Shaocong Mo[1], Bryan Jin[1], Haoshu Cui[1], Huaxing Zhan[3], Feifei Luo[1*], Jie Liu[1*]

Affiliations:

[1]Department of Digestive Diseases, Huashan Hospital, Fudan University; Shanghai, 200040, China.

[2]Biotherapy Research Center, Fudan University; Shanghai, 200032, China.

[3]Xiamen Ginposome Pharmaceutical Co., Ltd; Xiamen, 361026, China.

[†]These authors contributed equally to this work.

* Correspondence: Dr. Jie Liu, Tel/Fax: +86-21-52888236, E-mail: jieliu@fudan.edu.cn; or Dr. Feifei Luo, Tel/Fax: +86-21-52888234, E-mail: feifeiluo@fudan.edu.cn. Department of Digestive Diseases, Huashan Hospital, Fudan University, 12 Wulumuqi Middle Road, Shanghai 200040, China.

The PDF file includes:

Supplemental Methods; Supplemental Figures 1 to 23; Supplemental Tables 1 to 17; References

Supplemental Methods

Tumor model and treatment

MC38 (3×10^5 per mouse) and Panc02 (1×10^6 per mouse) tumor cells suspended in 100 µL of PBS were implanted subcutaneously in the right flank of C57BL/6J mice. Treatment was initiated after the tumor nodules grew to 60 mm^3, and mice were randomly assigned to 8 treatment groups (each group comprising 6 mice), including PBS (equivalent volume), DMSO, PTX, PTX-lipo, Rg3-lipo, PTX combined with Rg3-lipo, Nab-PTX, or PTX-Rg3-lipo. These treatments were administered via tail vein injections every other day. The dosages provided consisted of PTX 10 mg/kg body weight and/or Rg3 15 mg/kg body weight. Tumor volume was measured every other day and calculated as $0.5 \times \text{length} \times \text{width}^2$. In the MDSC depletion experiment, mice were intraperitoneally injected with IgG isotype-matched control antibody (BioXcel, CT, USA; LTF-2), or Gr-1 antibody (BioXcel, CT, USA; RB6-8C5, 100 µg per mouse) every other day.

For the acute toxicity study, C57BL/6J mice were injected subcutaneously with 100 µL of MC38 tumor cell suspension (3×10^5 per mouse). When tumors reach 100 mm^3, mice are randomly assigned to different treatment groups and a single dose of the drug is administered intravenously. Mice are observed for 24 hours after dosing and blood is collected for toxicity assessment. For the long-term toxicity study, the same tumor-bearing mouse model has been established as previously described. When tumors reach 100 mm^3, the drug is administered by the same route every other day for 25 days. Body weight and activity are recorded daily throughout the study. Blood samples are collected at the end of the study for toxicity evaluation.

Cytotoxicity test

We evaluated the antitumor potency of PTX, PTX-lipo, Rg3-lipo, and PTX-Rg3-lipo by calculating the IC50 of the drugs (the concentration of a drug that inhibits tumor cell proliferation by 50%). We set up blank wells (samples without cells to correct the background interference of the system), control group (samples without any drugs to measure the baseline of cell activity), and experimental groups (containing different

concentrations of drugs to observe the effect of drugs on biological activity), respectively. Cell viability was then assayed using the CCK8 kit and microplate reader.

For data processing, we employed the following formula for normalization: Normalized cell viability = $(OD_{experimental\ group} - OD_{blank\ wells}) / (OD_{control\ group} - OD_{blank\ wells}) \times 100\%$. Here, the control group represented the cell viability when no drug was added. The normalized data was then analyzed by non-linear regression using GraphPad Prism and the concentration of the drug that reduced cell viability to 50% was identified as IC50.

Apoptosis assays

Cells were seeded at 2×10^5 cells/well in a 24-well plate overnight and treated with an equivalent concentration of PTX for 24 h. At the end of treatments, single-cell suspensions were blocked with the anti-mouse CD16/CD32 (clone 93) mAb for murine cells and stained with Annexin V (BD Biosciences, CA, USA) for 30 min at 4°C, then with 7AAD (Invitrogen, CA, USA) for 5 min at room temperature. The percentages of apoptotic and necrotic cells were measured using a BD FACSCalibur flow cytometer (BD Biosciences, CA, USA) and analyzed using FlowJo software version 10 (FlowJo LLC).

Quantitative RT-PCR (qPCR)

Cells were harvested and resuspended in 1 mL of TRIzol (Invitrogen, CA, USA) and the mixture was frozen at -80°C until used. For RNA extraction, 0.2 mL of chloroform was added to the samples and mixed thoroughly. Samples were incubated at room temperature for 3 min before centrifugation for 15 min at 12,000 rpm (4°C). The aqueous phase was removed and mixed with an equal volume of isopropanol. The RNA pellet was collected via centrifugation and washed with 70% ethanol. The pellet was then dissolved in 20 µL of nuclease-free water. RNA from each sample was used to generate cDNA using a PrimeScript RT Reagent Kit (Takara Biotechnology, Dalian, China) according to the manufacturer's instructions. qPCR was performed using the Hieff qPCR SYBR Green Master Mix (Yeasen, Shanghai, China) on a 7500 Real-Time PCR System (Applied Biosystems, CA, USA). The amount of target mRNA was normalized to the expression level of β-actin generated from the same sample and subsequently to the controls. Relative expression was calculated as $2^{-\Delta Ct}$, while fold induction was calculated as $2^{-\Delta\Delta Ct}$(*1*). The primers are listed in Supplemental Table 17.

In vitro T cell suppression experiments

T cells were isolated from the spleen of C57BL/6J mice using a Miltenyi Pan T Cell Isolation Kit II (Miltenyi Biotec, NRW, Germany). For proliferation assay, T cells were labeled with 5 μM of CellTrace Violet (CFSE, Invitrogen, CA, USA) at 37°C for 20 min, washed with PBS containing 1% FBS, and counted before being co-cultured with MDSCs. Bone marrow MDSCs were seeded and pretreated with an equivalent concentration of PTX as indicated for 24 h. MDSCs were washed to remove the drug and tumor-conditioned medium before co-culturing with T cells. After two days, T cells were harvested for flow cytometry.

Flow cytometry profiling of tumor-infiltrating leukocytes

Single-cell suspensions from tumor-infiltrating leukocytes were blocked with an anti-mouse CD16/CD32 (Invitrogen, CA, USA; clone 93) mAb for 10 min at 4°C and stained with antibodies to surface markers for 30 min at 4°C. For intracellular cytokine detection, cells were harvested and stained with cell surface antibodies for 30 min at 4°C, washed in PBS, and fixed in 100 μL IC fixation for 30 min. The cells were stained with intracellular antibodies at room temperature for 30 min to analyze their activation status, according to the manufacturer's instructions. To ensure the reliability of our results, we employed untreated cells to assess baseline cellular characteristics, and in some experiments, we incorporated positive controls to validate the effectiveness of the flow cytometry procedure. We collected an average of 5×10^5 cells per sample to guarantee the statistical robustness of our data and consistency across all samples. Data were acquired using a BD FACSCalibur flow cytometer (BD Biosciences, CA, USA) and analyzed using FlowJo. All antibody information is listed in Supplemental Table 16.

Isolation of tumor-infiltrating leukocytes and peripheral blood mononuclear cells

Tumors were cut into small pieces and enzymatically digested for 1 h at 37°C with 200 rpm shaking using Tumor Dissociation Kit (Miltenyi Biotec, NRW, Germany). After digestion, the remaining tissue pieces were dissociated using the gentleMACS Octo Dissociator, and cell suspensions were filtered through a 100 μm cell-strainer (Corning, NY, USA) in PBS, and overlayed on the top of 40% and 70% Percoll (Sigma-Aldrich, MO, USA). After centrifuging at 2,000 rpm for 20 min at 20°C, the tumor-infiltrating leukocytes were obtained from the interface of 40%/70% Percoll solution. Finally, the cells were resuspended in cold PBS for further analysis.

Fresh blood was collected from the tumor-bearing mice and mixed at a ratio of 1:1 (volume/volume) with PBS at room temperature and layered to Ficoll (GE Healthcare Bio-Sciences, PA, USA) in tubes and centrifuged at 2,000 rpm for 20 min at 20°C. Peripheral blood mononuclear cells were obtained from the Ficoll solution interface. Red blood cells were lysed in ACK lysis buffer for 3 min at room temperature and washed with PBS. Finally, cells were resuspended in cold PBS for further analysis.

Seahorse assay

An Agilent Seahorse XF Real-Time ATP Rate Assay Kit (Agilent, CA, USA; 103592-100) was used to measure the rate of ATP production through mitochondrial oxidative phosphorylation and glycolysis. The oxygen consumption rate (OCR), extracellular acidification rate (ECAR), and ATP production rate were assessed using an XFe96 Extracellular Flux Analyzer (Agilent, CA, USA) following the manufacturer's instructions.

For the measurements, the probe plate was hydrated with HPLC-grade water in a CO_2-free incubator the day before and the solution was replaced with XF Calibrant the following day. The plates were incubated in a CO_2-free incubator at 37°C for at least 1 h. For all assays, MDSCs were pretreated with the drugs for 24 h. After the treatment period, MDSCs were seeded in poly L-lysine-coated XF96 cell culture microplates (Agilent, CA, USA; 101085-004) at a density of 30,000 cells per well. The cells were cultured in Seahorse XP RPMI medium (Agilent, CA, USA; 103576-100) supplemented with 5 mM glucose (Sigma, MO, USA; G8270-100G), 2 mM l-glutamine (Thermo Fisher Scientific, CA, USA; 25030081), 1 mM sodium pyruvate, and 1% FBS.

RNA-sequencing

Total RNA from MDSC was extracted using TRIzol reagent (Invitrogen, CA, USA) following the manufacturer's instructions. RNA integrity was assessed through agarose gel electrophoresis (28S:18S ≥ 1.5), RNA purity was determined using a Nanodrop spectrophotometer, and RNA concentration was precisely quantified with Qubit fluorometry. cDNA libraries were prepared using the Novaseq 6000 S4 Reagent Kit v1.5 (300 cycles) (Illumina, CA, USA) and sequenced on a Novaseq 6000 (Illumina, CA, USA). Quality control of the sequencing results was done by FastQC and the data was further filtered with trim_galore. Reads were aligned to the Mus musculus GRCm38 reference genome using STAR. Differential

expression analysis utilized DESeq2, defining differential genes as those with at least a two-fold change and an FDR of 5%. Functional enrichment analysis was conducted using clusterProfiler, and pathway information was obtained by KEGG databases. The RNA-sequencing was performed in Genenergy (Shanghai, China). Single-cell RNA-sequencing (scRNA-Seq) dataset GSE132257 was used to determine the predominant expression of glucose transporter genes in specific cell types (*2*).

In vivo imaging

Tumor-bearing mice were administered C-LPs or Rg3-LPs containing DiR through tail vein injection. After 8 h, the mice were imaged using the IVIS Spectrum system (PerkinElmer, USA) and then euthanized. The heart, liver, spleen, lungs, kidneys, and tumors were collected and imaged at 8 h with the IVIS Spectrum system.

siRNA transfection

The siRNA was designed by Suzhou Genepharma Co., Ltd. to downregulate Glut3, Maf, and Mafb expression levels. siRNA sequences were as follows: Control-siRNA, 5'-UUCUUCGAACGUGUCACGUTT-3'; Slc2a3-siRNA, 5'-GCCAUGAGCUUUGUCUGUATT-3'; Maf-siRNA, 5'-GGCCAUGGAAUAUGUUAAUTT-3'; Mafb-siRNA, 5'-AACGACUUCGACCUUCUCATT-3', Slc2a1-siRNA, 5'-CACUGCAGUUCGGCUAUAATT-3'. MDSCs were transfected with either siRNA using electroporation with an ECM830 Electro Square Wave Porator (Harvard Apparatus BTX, MA, USA). Briefly, MDSCs were harvested, washed three times in pre-chilled Opti-MEM (Gibco), and resuspended in pre-chilled Opti-MEM. Electroporation was performed at 500 Volt (300 ms per pulse). Subsequently, cells were transferred to pre-warmed Opti-MEM and follow-up experiments were performed at 24 h post-transfection. Downregulation of Glut3 was measured using qPCR.

Western blotting

The cells were collected via centrifugation and lysed in RIPA lysis buffer (Thermo Fisher Scientific, Inc., CA, USA). The protein concentration was determined using a BCA protein assay kit (Beyotime Institute of Biotechnology, Shanghai, China), and the sample loading buffer (Beyotime Institute of Biotechnology, Shanghai, China) was added to the protein sample for SDS-PAGE. Proteins were separated by non-reducing SDS-PAGE 10% Tris-HCl gels (Beyotime Institute of Biotechnology, Shanghai, China) and transferred to

PVDF membranes (Millipore, MA, USA), which were then washed in Tris-buffered saline with 0.1% Tween-20 and blocked with 5% milk. Next, the membranes were incubated with primary anti-c-Maf antibody (Abcam, Cambs, UK; ab243901; 1:1,000), anti-Mafb antibody (Abcam, Cambridge, UK; ab243902; 1:1,000), and anti-Vinculin antibody (CST Biological Reagents Co., Ltd., MA, USA; 13901S; 1:1,000) overnight at 4°C and washed with TBST. The cells were then incubated with a horseradish peroxidase-conjugated anti-rabbit IgG secondary antibody (CST Biological Reagents Co., Ltd., MA, USA; 7074S; 1:5,000) for 90 min at 50 rpm. Finally, the blots were visualized using an ImageQuant LAS 4000 mini scanner (GE Healthcare Bio-Sciences, PA, USA) and bands were quantified using ImageJ software.

Supplemental Figures

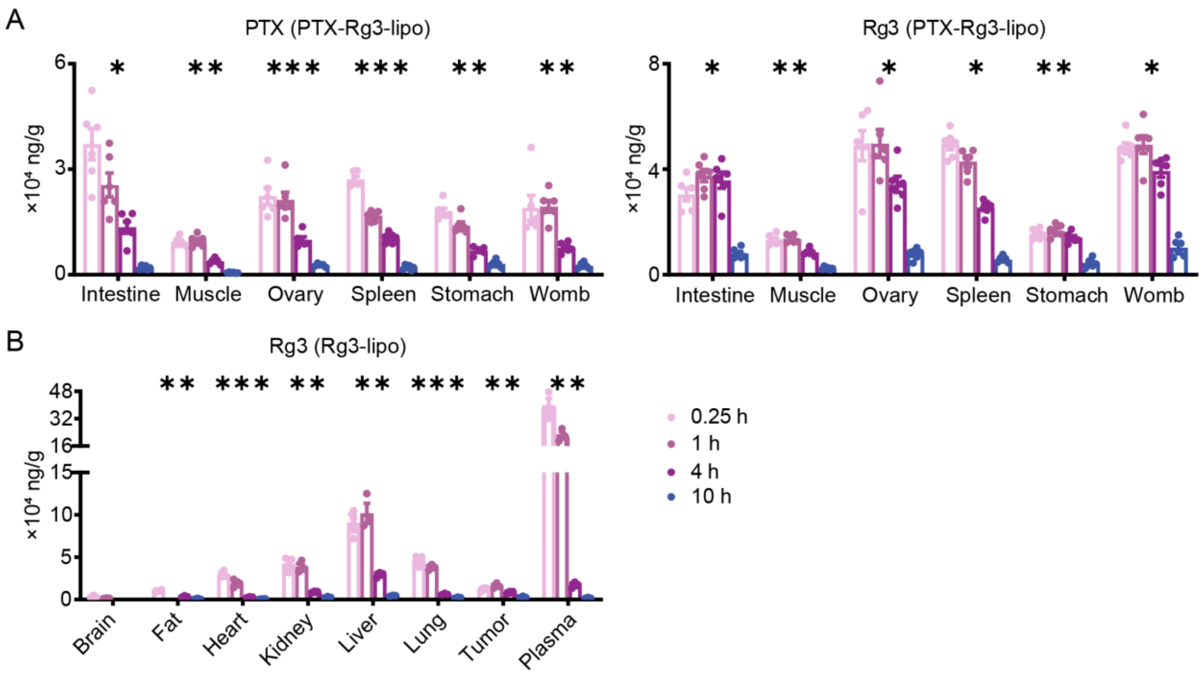

Fig. S1. Tissue Distribution of PTX and Rg3 in tumor bearing mouse model. (A)Tissue PTX and Rg3 concentrations following a single intravenous injection of PTX-Rg3-lipo (PTX 30 mg/kg and Rg3 45 mg/kg) in human gastric cancer SNU-16 subcutaneous transplantation mouse model (n = 6). (B)Tissue Rg3 concentrations following a single intravenous injection of Rg3-lipo (Rg3 45 mg/kg) in human gastric cancer SNU-16 subcutaneous transplantation mouse model (n = 6). Data are shown as mean ± SEM. One-way ANOVA (A, B) with a post hoc Bonferroni test was used for statistical analysis. *, $P < 0.05$; **, $P < 0.01$; ***, $P < 0.001$.

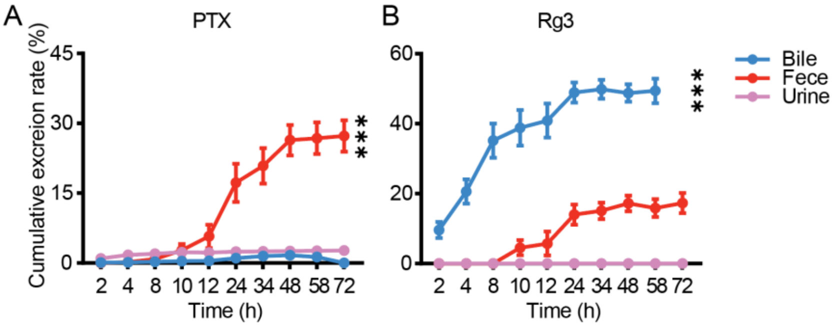

Fig. S2. Drug excretion assay in SD rats after single intravenous injection (n = 6). Data are shown as mean ± SEM. Two-way ANOVA with a post hoc Bonferroni test (A, B) was used for statistical analysis. ***, $P < 0.001$.

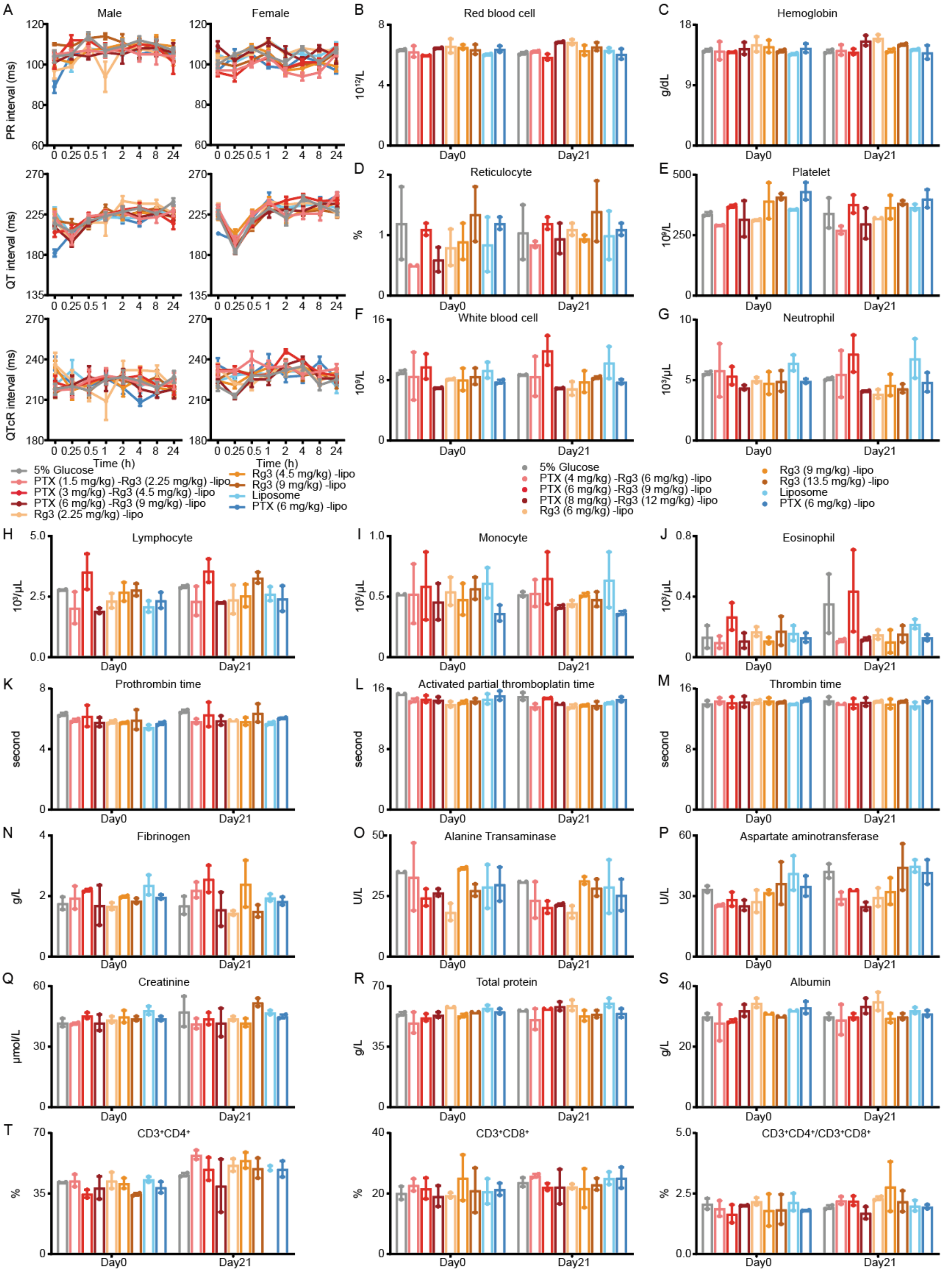

Fig. S3. Safety study of PTX-Rg3-lipo in the beagle dog model. (A) Effects of PTX-Rg3-lipo on beagle dogs' cardiovascular system using electrocardiogram tests with telemetry implants (n = 4). The effects of a single intravenous dose of PTX-Rg3-lipo on (B-J) hematology and (K-N) coagulation function, (O-S) serum

biochemistry, and (T) immune system in beagle dogs (n = 2). Data are shown as mean ± SEM. One-way ANOVA (B-T) and two-way ANOVA (A) with a post hoc Bonferroni test were used for statistical analysis.

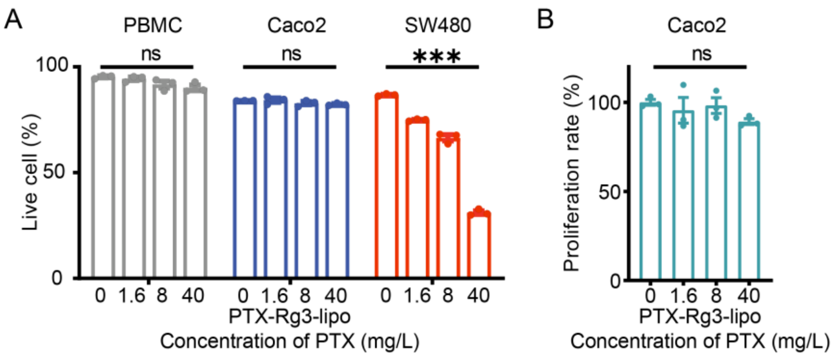

Fig. S4. PTX-Rg3-lipo has minimal effect on the immune system. (A) The peripheral blood mononuclear cells (PBMCs), normal intestinal epithelial cells (Caco2), and cancer cells (SW480) were stained with Annexin V and 7-AAD Viability Staining Solution after PTX-Rg3-lipo treatment. Live cells are defined as Annexin V^- $7AAD^-$. (B) Effect of PTX-Rg3-lipo on the proliferation of normal intestinal epithelial cells. Data are shown as mean ± SEM. One-way ANOVA with a post hoc Bonferroni test (A, B) was used for statistical analysis. ***, $P < 0.001$.

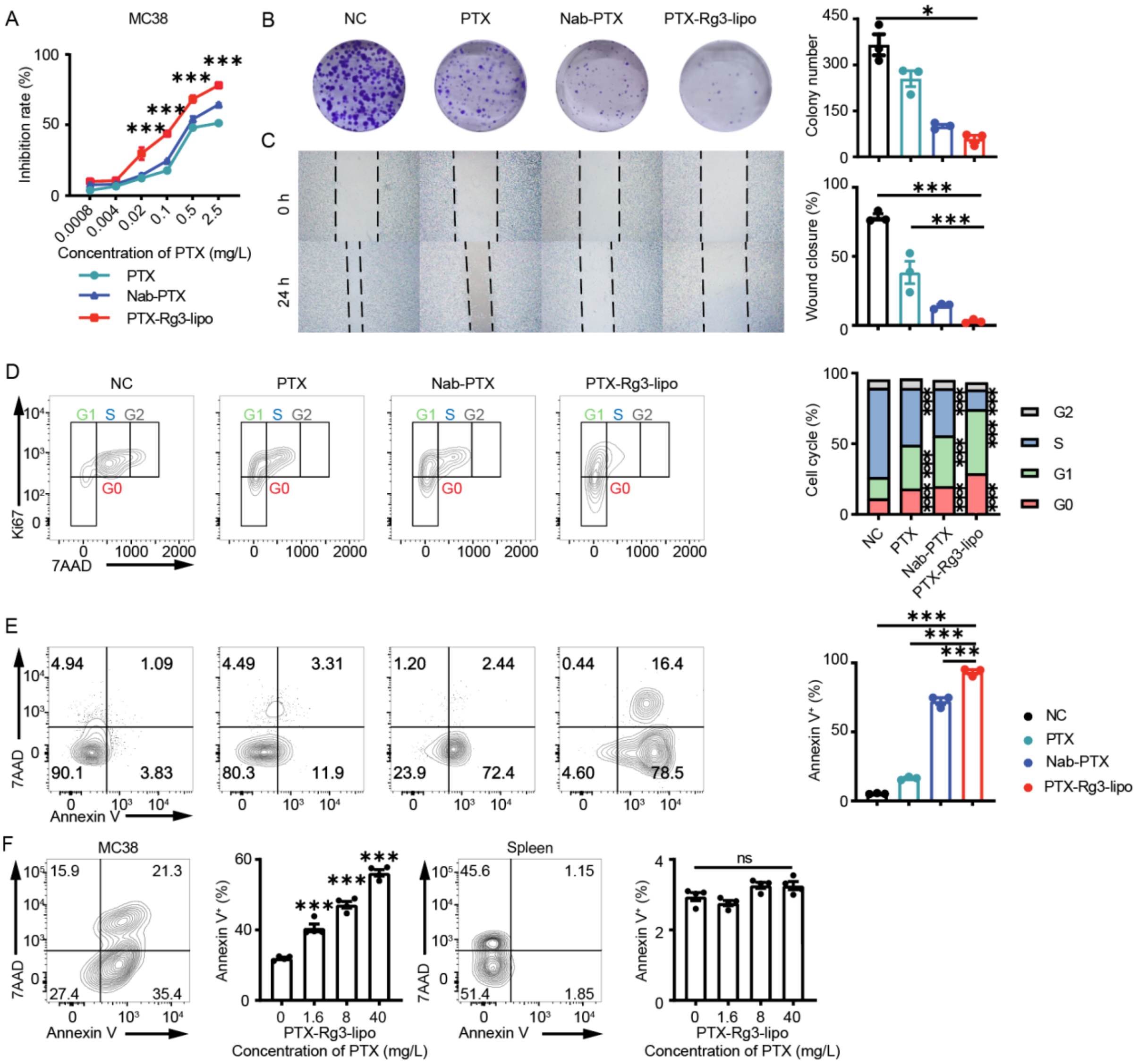

Fig. S5. PTX-Rg3-lipo exhibits antitumor activity *in vitro*. (A) The inhibitory rates of PTX, Nab-PTX, and PTX-Rg3-lipo (equivalent to indicated PTX concentration) on MC38 cells. (B) Tumor cell proliferation was measured using a colony formation assay. (C) The migratory ability of the cells was assessed using wound healing assays. (D) The cell cycles in MC38 cells are shown by representative flow cytometry plots. (E) Frequencies of apoptosis MC38 cells are shown by representative flow cytometry plots and quantification. (F) Frequencies of apoptotic MC38 cells and spleen of normal mice are shown by representative flow cytometry plots and quantification. Data are shown as mean ± SEM. One-way ANOVA (B-F) and two-way ANOVA (A) with a post hoc Bonferroni test were used for statistical analysis. *, $P < 0.05$; ***, $P < 0.001$.

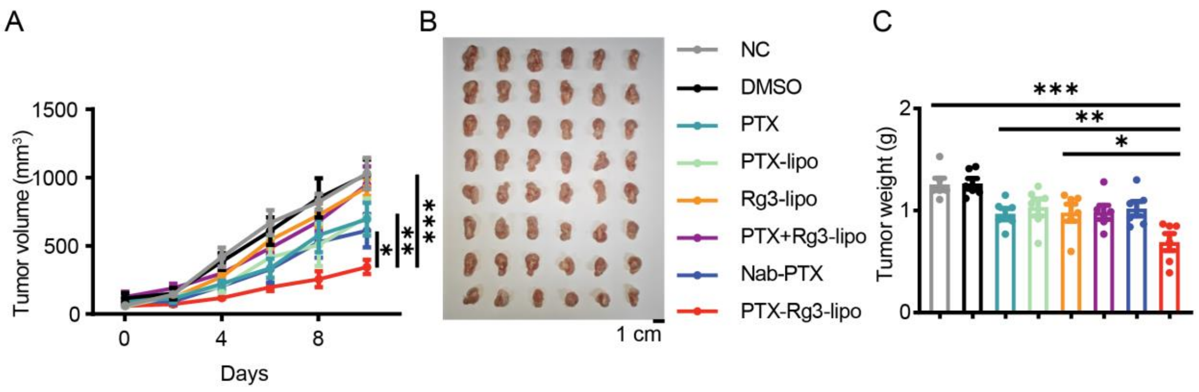

Fig. S6. PTX-Rg3-lipo suppresses tumor growth in immunodeficient (NSG) murine model. (A) (B) The tumor growth curves and tumor image in each group (n = 6). (C) Quantification of tumor weights in each group. Data are shown as mean ± SEM. One-way ANOVA (C) and two-way ANOVA (A) with a post hoc Bonferroni test were used for statistical analysis. *, $P < 0.05$; **, $P < 0.01$; ***, $P < 0.001$.

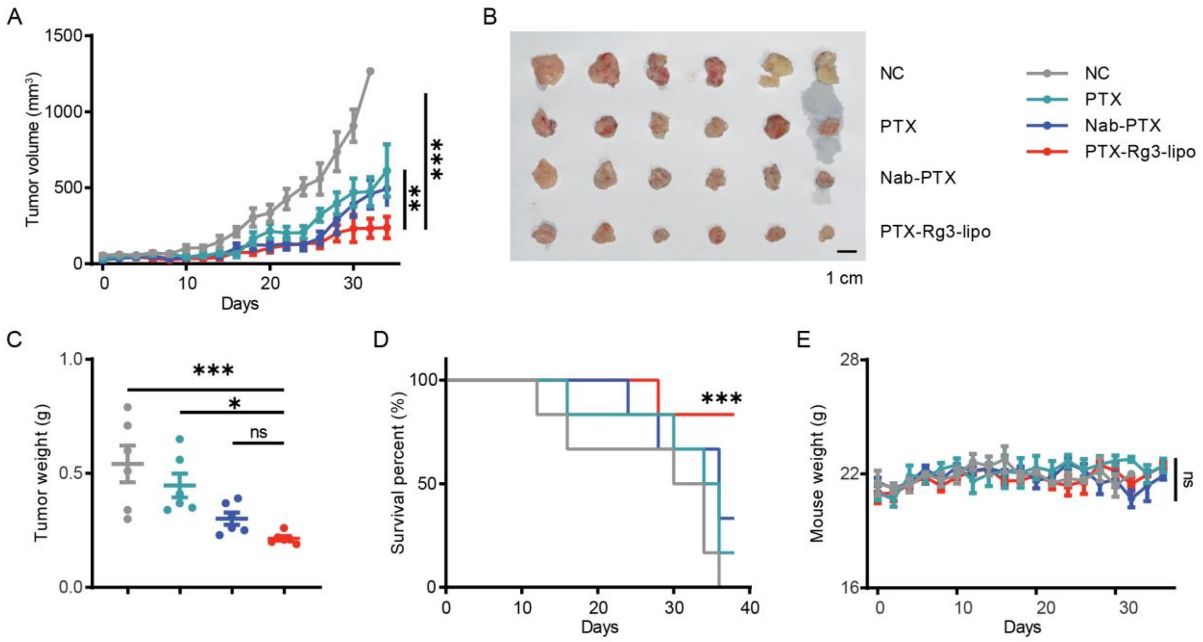

Fig. S7. PTX-Rg3-lipo suppresses tumor growth and prolongs survival in pancreatic cancer. (A) (B) The tumor growth curves and tumor image of Panc02 (n = 6). (C) Quantification of tumor weights in each group. (D) Thesurvival percent of mice in each group. (E) Body weight curves of each group (n = 6). Data are shown as mean ± SEM. One-way ANOVA (C) and two-way ANOVA (A, E) with a post hoc Bonferroni test, as well as the log-rank (Mantel-Cox) test (D), were used for statistical analysis. *, P < 0.05; **, P < 0.01; ***, P < 0.001.

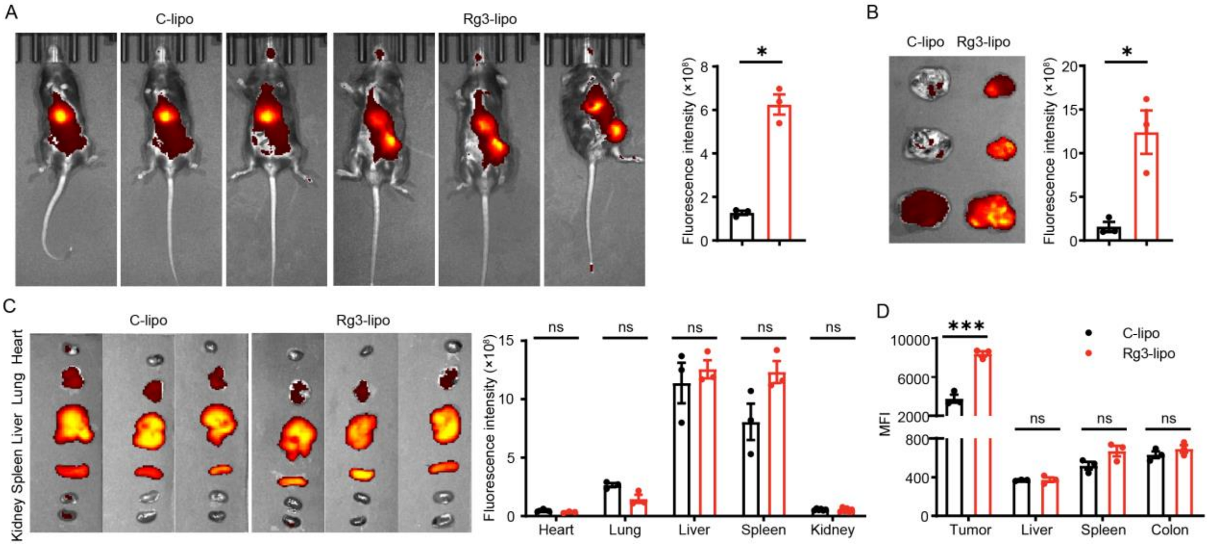

Fig. S8. Rg3-lipo enhances liposomes to accumulate in tumors. (A) Biodistribution and quantification of DiR-labeled C-lipo and Rg3-lipo after intravenous injection into MC38 tumor-bearing mice at 8 h. (B) Fluorescence images and quantification of excised tumors at 8 h. (C) Fluorescence images and quantification of dissected organs from mice sacrificed at 8 h. (D) Distribution of coumarin 6-labeled Rg3-lipo in tumor, liver, spleen and intestine. Data are shown as mean ± SEM. An unpaired two-tailed *t*-test (A-D) was used for statistical analysis. *, $P < 0.05$; ***, $P < 0.001$.

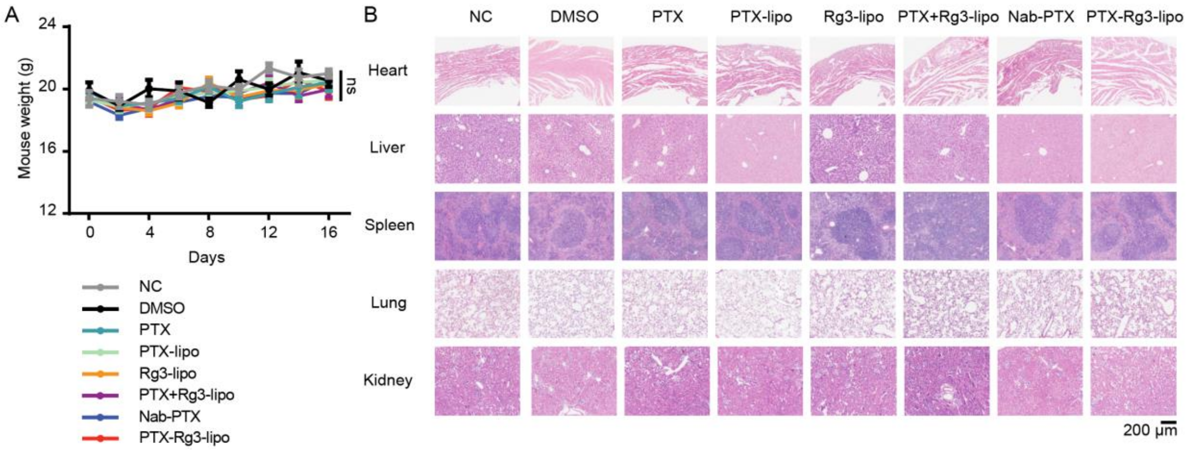

Fig. S9. PTX-Rg3-lipo exhibits a favorable safety profile. (A) Body weight curves of each group (n = 6). (B) Representative images of major organ sections from each group. Data are shown as mean ± SEM. Two-way ANOVA with a post hoc Bonferroni test (A) was used for statistical analysis.

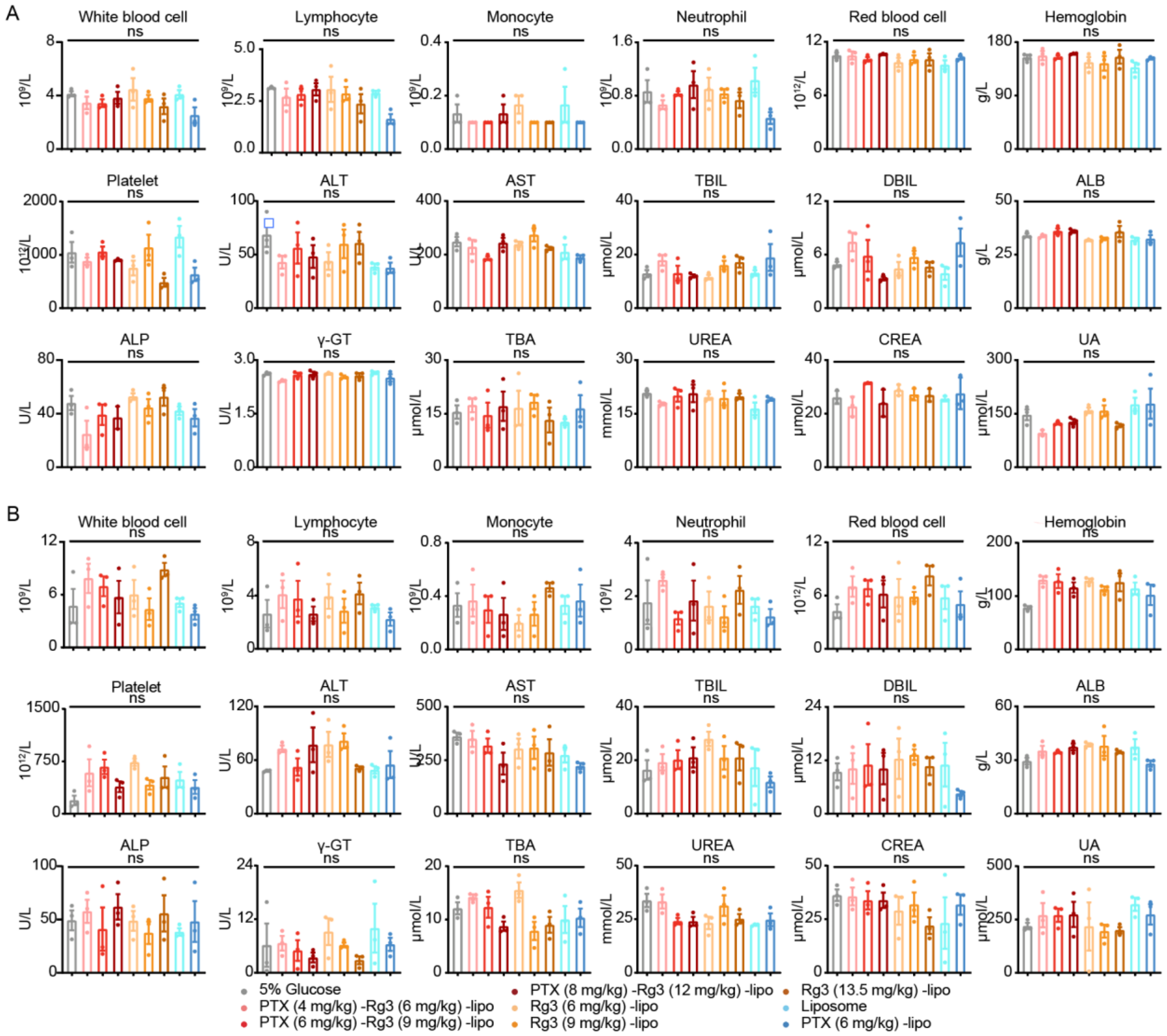

Fig. S10. PTX-Rg3-lipo demonstrates safety in terms of complete blood count and liver/renal functions. (A) The results of acute toxicity tests (n = 3). (B) The results of long-term toxicity tests (n = 3). Data are shown as mean ± SEM. One-way ANOVA with a post hoc Bonferroni test (A, B) was used for statistical analysis.

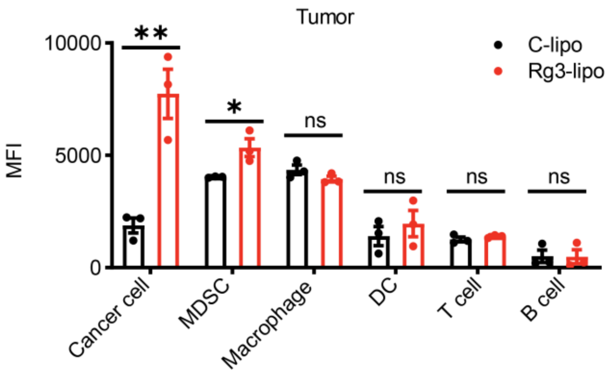

Fig. S11. The cellular distribution of Rg3-lipo within the tumor. Data are shown as mean ± SEM. An unpaired two-tailed *t*-test was used for statistical analysis. *, $P < 0.05$; **, $P < 0.01$.

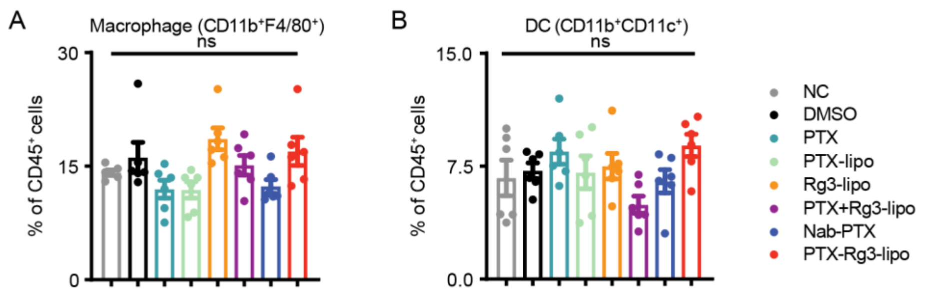

Fig. S12. Frequency of macrophage and DC in the tumor. Data are shown as mean ± SEM. One-way ANOVA with a post hoc Bonferroni test (A, B) was used for statistical analysis.

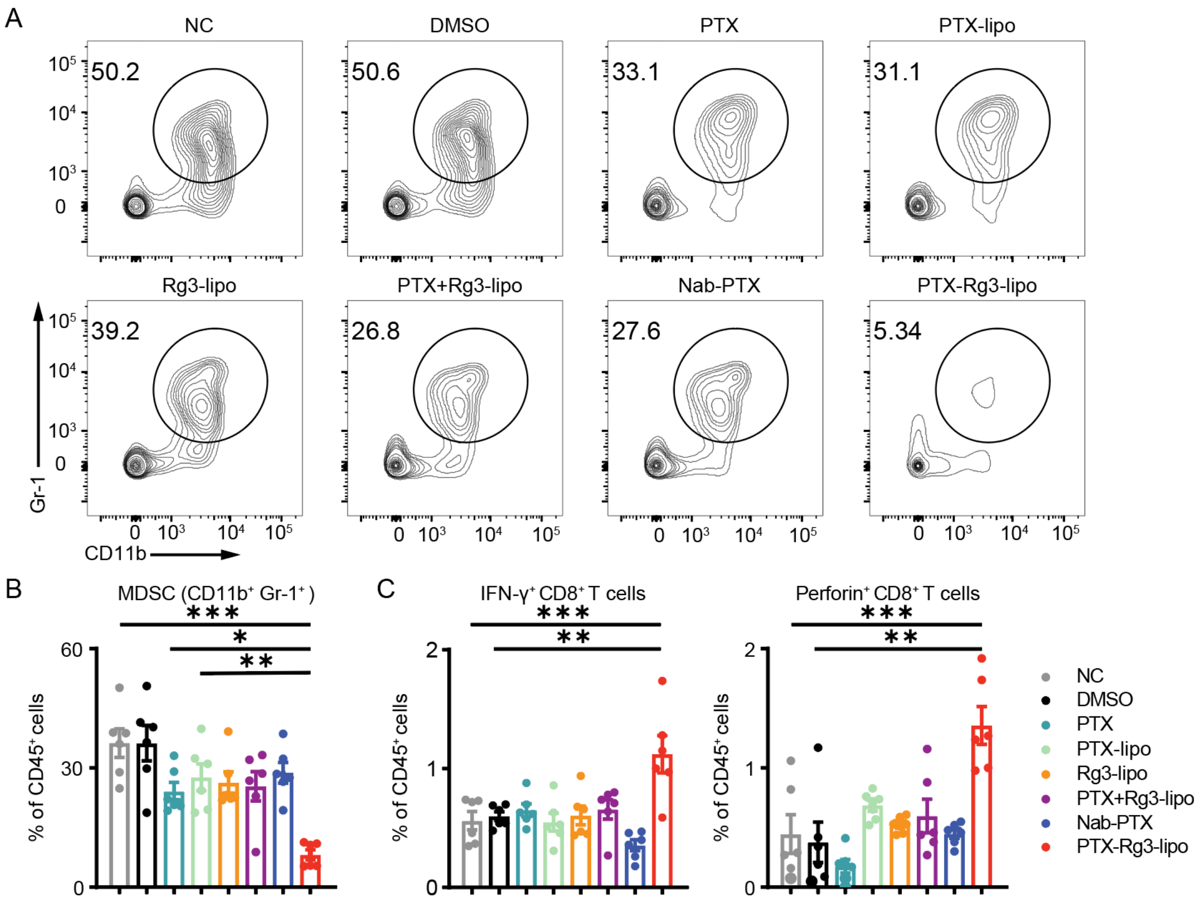

Fig. S13. PTX-Rg3-lipo alleviates immunosuppression in the peripheral by suppressing MDSCs and activating T-cell immune responses. (A) (B) Frequencies of MDSC ratio in peripheral from each group. (C) IFN-γ and Perforin production by $CD8^+$ T cells in the peripheral from each group. Data are shown as mean ± SEM. One-way ANOVA with a post hoc Bonferroni test (B, C) was used for statistical analysis. *, P < 0.05; **, P < 0.01; ***, P < 0.001.

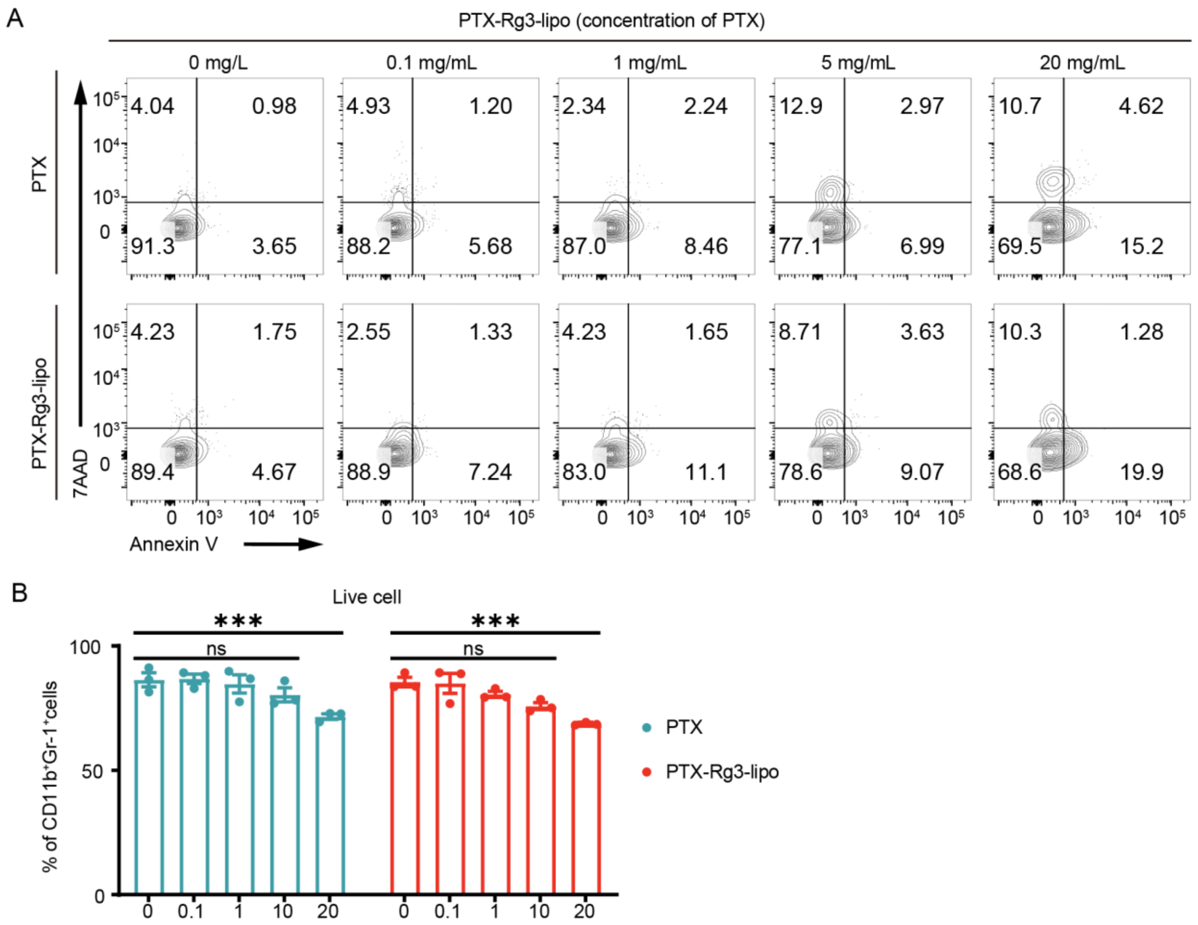

Fig. S14. PTX-Rg3-lipo induces MDSC cell death only at very high doses. (A) (B) Frequencies of live MDSC cells are shown by representative flow cytometry plots and quantification. Data are shown as mean ± SEM. One-way ANOVA with a post hoc Bonferroni test (B) was used for statistical analysis. ***, P < 0.001.

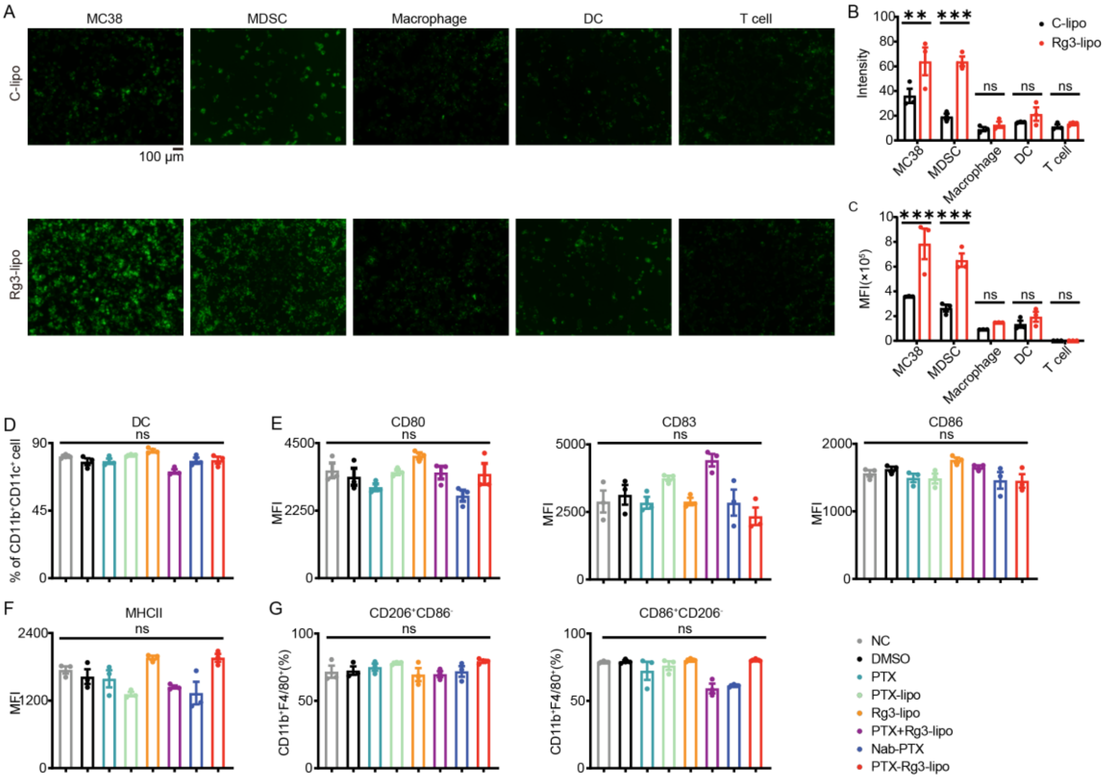

Fig. S15. PTX-Rg3-lipo has no significant effect on macrophages and DCs. (A) Fluorescence images demonstrating the uptake of C-lipo and Rg3-lipo by MC38 cells, MDSCs, macrophages, DCs, and T cells. (B) Quantitative analysis of liposome uptake levels. (C) Flow cytometry analysis of l the uptake of C-lipo and Rg3-lipo in the different cell types. (D) (E) (F) PTX-Rg3-lipo had little effect on the proportion of DCs and their maturation and antigen-presenting capacity. (G) PTX-Rg3-lipo also had no significant effect on the M1/M2 ratio of macrophages. Data are shown as mean ± SEM. An unpaired two-tailed *t*-test (B, C) and One-way ANOVA with a post hoc Bonferroni test (D-G) was used for statistical analysis. **, P < 0.01; ***, P < 0.001.

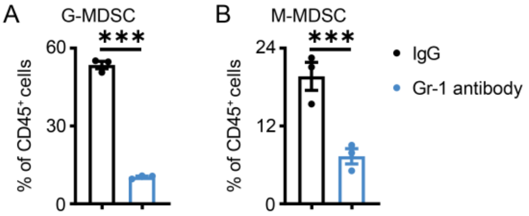

Fig. S16. The MDSC depletion model using the Gr-1 antibody was established. (A) (B) The knockdown efficiency of G-MDSC and M-MDSC in tumors (n = 3). Data are shown as mean ± SEM. An unpaired two-tailed *t*-test (A, B) was used for statistical analysis. ***, P < 0.001.

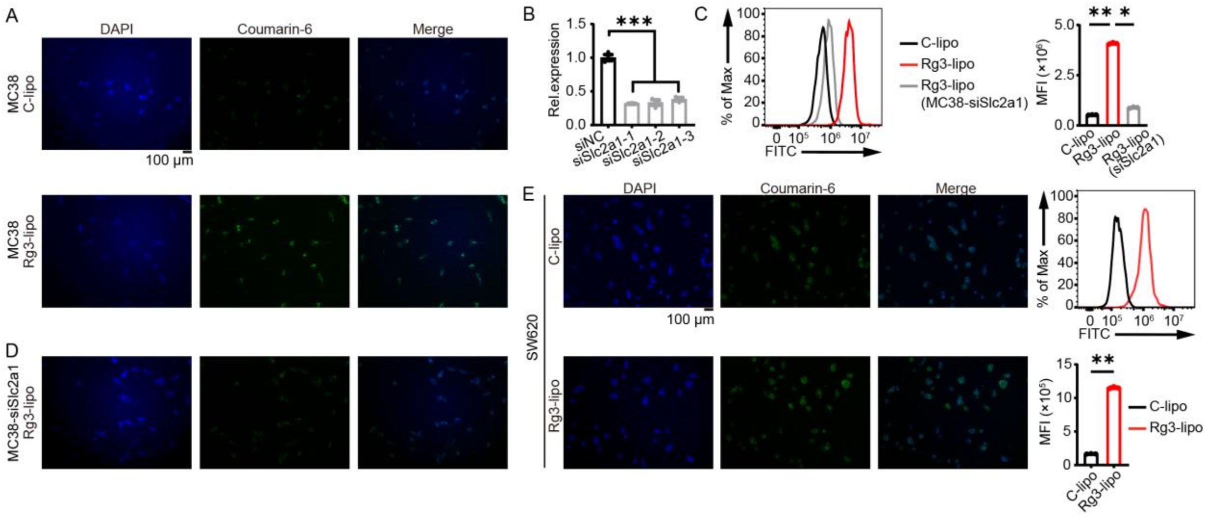

Fig. S17. Rg3-lipo targets tumor cells via Glut1. (A) Uptake of C-lipo and Rg3-lipo by MC38 cells. (B) The knockout efficacy of Glut1 in MC38 cells. (C) Flow cytometry analysis of C-lipo and Rg3-lipo uptake by MC38 cells and the effect of Glut1 deletion on Rg3-lipo uptake. (D) Uptake of MC38 following Glut1 deletion and Rg3-lipo treatments. (E) Microscopic and flow cytometric analysis of C-Lipo and Rg3-Lipo uptake by SW620 cells. One-way ANOVA with a post hoc Bonferroni test (B, C) and an unpaired two-tailed *t*-test (E) were used for statistical analysis. *, $P < 0.05$; **, $P < 0.01$; ***, $P < 0.001$.

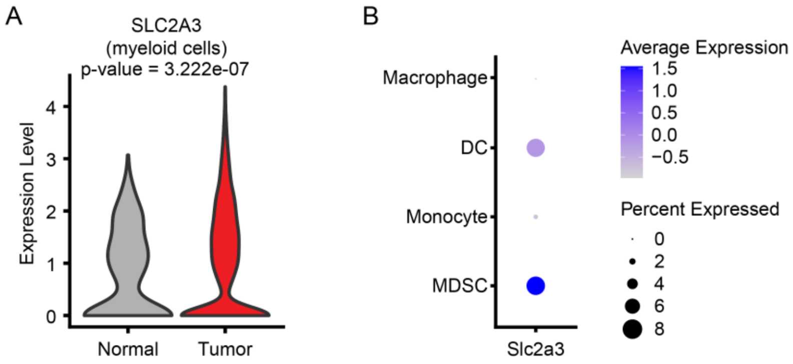

Fig. S18. Single-cell analysis of Glut3 expression distribution. (A) Glut3 expression in myeloid cells from colorectal cancer tissues and adjacent normal tissues. (B) Glut3 expression in macrophage, DC, monocyte and MDSC within colorectal cancer tissues. The Wilcoxon Rank-Sum test (A) was used for statistical analysis.

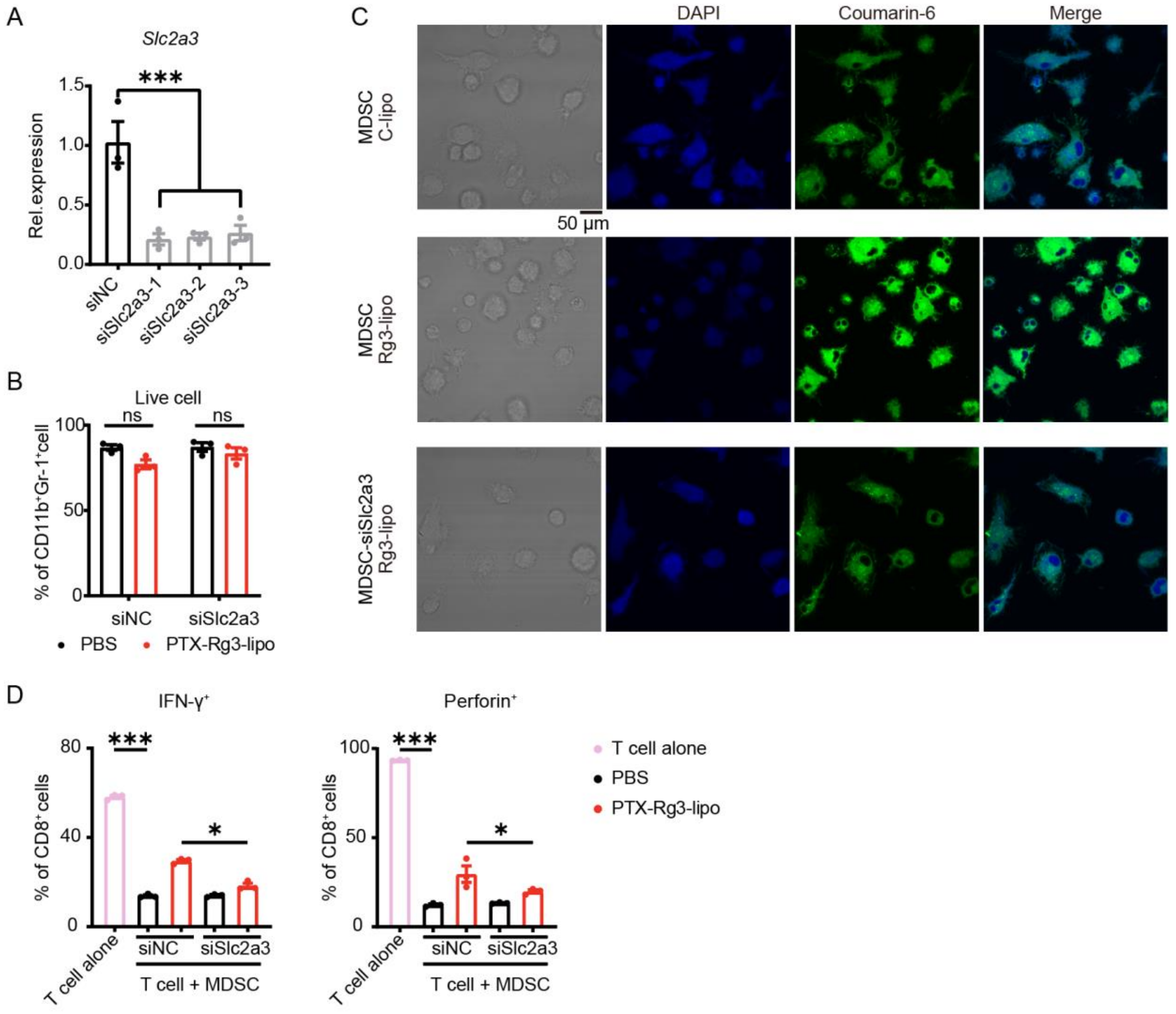

Fig. S19. The blockage of MDSC immunosuppression by PTX-Rg3-lipo is dependent on Glut3. (A) The knockout efficacy of Glut3 in MDSCs. (B) Proportion of live MDSCs after knockdown of Glut3 on the surface of MDSCs. (C) C-lipo and Rg3-lipo uptake in MDSC and changes in Rg3-lipo uptake in MDSC after knockdown of Glut3. (D) Alteration in the function of T cell cytokine production following Glut3 deletion and PTX-Rg3-lipo treatments. Data are shown as mean ± SEM. One-way ANOVA with a post hoc Bonferroni test (A, B, D) was used for statistical analysis. *, $P < 0.05$; ***, $P < 0.001$.

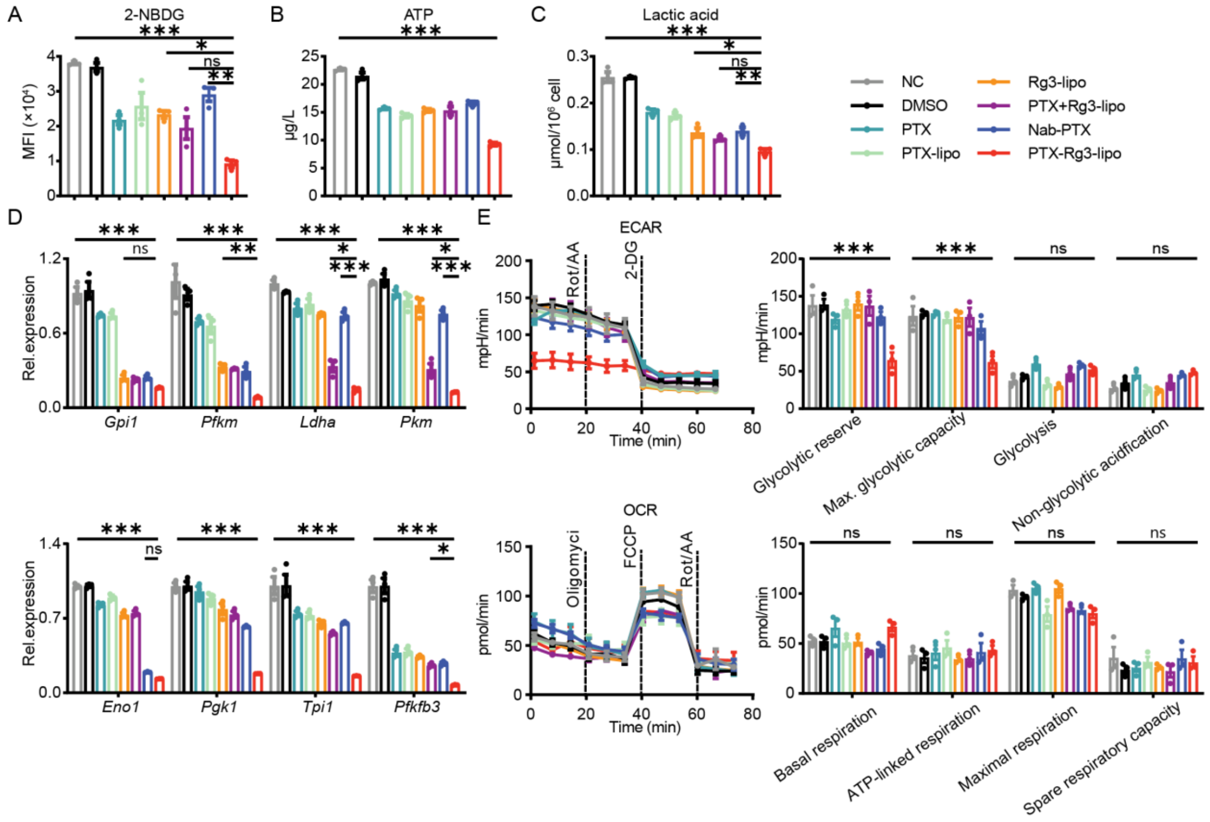

Fig. S20. The metabolic effects of the DMSO, PTX, PTX-lipo, Rg3-lipo, PTX combined with Rg3-lipo, and PTX-Rg3-lipo on MDSCs. (A) 2-NBDG assessment of glucose uptake changes following treatment. (B) (C) Quantification of ATP and lactic acid production. (D) Relative expression levels of glycolytic genes following treatment. (E) Seahorse analysis of MDSCs following treatment. Data are shown as mean ± SEM. One-way ANOVA (A-E) with a post hoc Bonferroni test were used for statistical analysis. *, P < 0.05; **, P < 0.01; ***, P < 0.001.

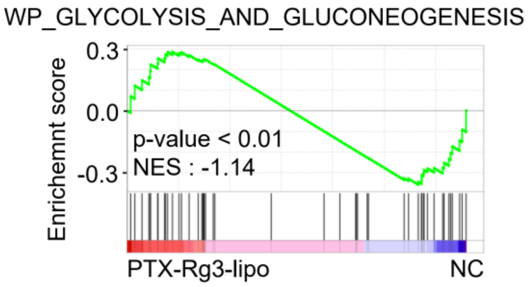

Fig. S21. GSEA was employed to reveal the alterations in the glycolysis pathway following PTX-Rg3-lipo intervention.

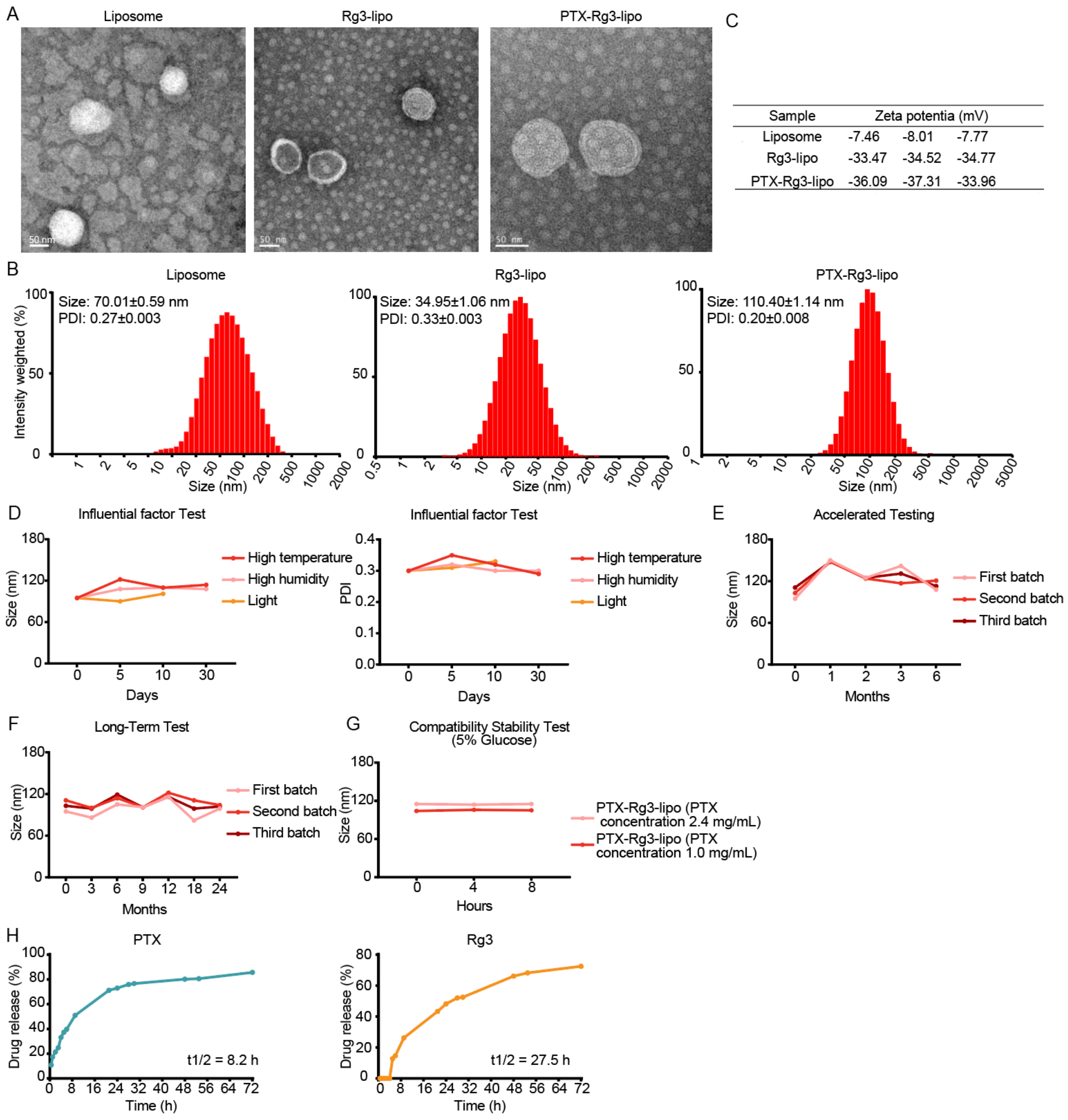

Fig. S22. Characterization and stability of liposomes. (A) (B) Size distribution of normal liposome, Rg3-lipo and PTX-Rg3-lipo. (C) Zeta potential of liposome, Rg3-lipo and PTX-Rg3-lipo. (D) (E) (F) (G) Influential factor tests, accelerated tests, long-term tests, and compound stability tests were used to evaluate the stability of PTX-Rg3-lipo. (H) Drug release curve of PTX-Rg3-lipo and t1/2 of PTX and Rg3 release.

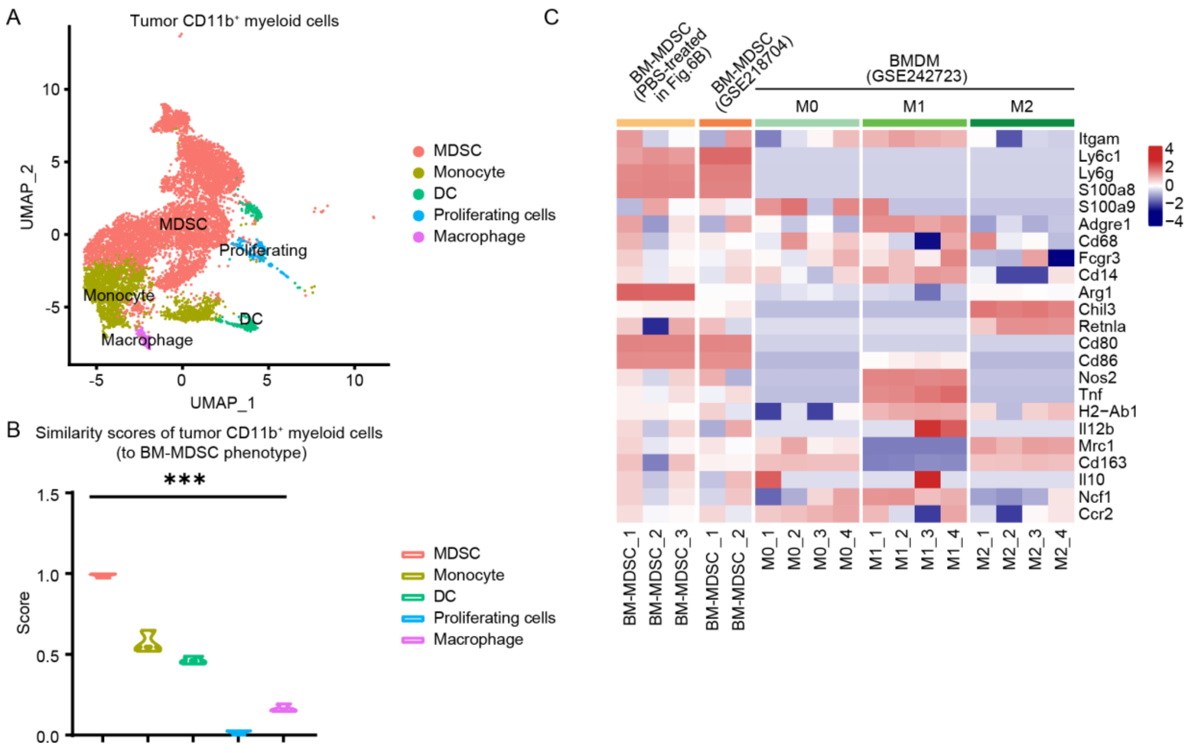

Fig. S23. Scientific validity of the bone marrow-induced MDSC method. (A) UMAP of tumor-localized myeloid cells (GSE244797). (B) Similarity scores of bone marrow-induced MDSCs compared to myeloid cell subpopulations. (C) Heatmap highlighting distinct variations in the expression of key markers reported in public databases in bone marrow-induced MDSCs and bone marrow-derived macrophages (BMDMs), indicating that the bone marrow-induced MDSCs in the current research are partially similar to the reference bone marrow-induced MDSCs, but distinctly different from BMDMs. The Wilcoxon Rank-Sum test (B) was used for statistical analysis. ***, $P < 0.001$.

Supplemental tables

Table S1. Summary of modified Irwin test observation results in male animals before administration

Group		S	V	L1	M1	H1	L2	M2	H2	C	P
Number of animals		n=5	n=5	n=5	n=5	n=5	n=5	n=5	n=5	n=5	n=5
Observation items within the cage	Piloerection										
	0	5	5	5	5	5	5	5	5	5	5
	1	0	0	0	0	0	0	0	0	0	0
	2	0	0	0	0	0	0	0	0	0	0
	Eyelid closure										
	0	5	5	5	5	5	5	5	5	5	5
	1	0	0	0	0	0	0	0	0	0	0
	2	0	0	0	0	0	0	0	0	0	0
Observation after removal from the cage	Resistance to handling										
	0	5	5	5	5	5	5	5	5	5	5
	1	0	0	0	0	0	0	0	0	0	0
	2	0	0	0	0	0	0	0	0	0	0
	3	0	0	0	0	0	0	0	0	0	0
	Body tension										
	0	0	0	0	0	0	0	0	0	0	0
	1	5	5	5	5	5	5	5	5	5	5
	2	0	0	0	0	0	0	0	0	0	0
	Skin color										
	0	0	0	0	0	0	0	0	0	0	0
	1	5	5	5	5	5	5	5	5	5	5
	2	0	0	0	0	0	0	0	0	0	0
	Lacrimation										
	0	5	5	5	5	5	5	5	5	5	5
	1	0	0	0	0	0	0	0	0	0	0
	Salivation										
	0	5	5	5	5	5	5	5	5	5	5
	1	0	0	0	0	0	0	0	0	0	0
	2	0	0	0	0	0	0	0	0	0	0
	3	0	0	0	0	0	0	0	0	0	0
	4	0	0	0	0	0	0	0	0	0	0

S: 5% glucose, 0 mg/kg
V: Liposome, 0 mg/kg
L1: PTX (7.5 mg/kg) -Rg3 (11.25 mg/kg) -lipo
M1: PTX (15 mg/kg) -Rg3 (22.5 mg/kg) -lipo
H1: PTX (30 mg/kg) -Rg3 (45 mg/kg) -lipo
L2: Rg3 (11.25 mg/kg) -lipo
M2: Rg3 (22.5 mg/kg) -lipo
H2: Rg3 (45 mg/kg) -lipo
C: PTX (15 mg/kg) -lipo
P: positive control group, 12 mg/kg chlorpromazine hydrochloride injection
The results are presented as frequencies. Compared to the 5% glucose group, $P > 0.05$.

Summary of modified Irwin test observation results in male animals before administration (Continued)

Group		S	V	L1	M1	H1	L2	M2	H2	C	P
Number of animals		n=5	n=5	n=5	n=5	n=5	n=5	n=5	n=5	n=5	n=5
Observation inside the observation box	Awakeness										
	0	0	0	0	0	0	0	0	0	0	0
	1	0	0	0	0	0	0	0	0	0	0
	2	5	5	5	5	5	5	5	5	5	5
	3	0	0	0	0	0	0	0	0	0	0
	4	0	0	0	0	0	0	0	0	0	0
	Loss of balance										
	0	5	5	5	5	5	5	5	5	5	5
	1	0	0	0	0	0	0	0	0	0	0
	2	0	0	0	0	0	0	0	0	0	0
	3	0	0	0	0	0	0	0	0	0	0
	Paralysis										
	0	5	5	5	5	5	5	5	5	5	5
	1	0	0	0	0	0	0	0	0	0	0
	Exophthalmos										
	0	5	5	5	5	5	5	5	5	5	5
	1	0	0	0	0	0	0	0	0	0	0
	2	0	0	0	0	0	0	0	0	0	0
	3	0	0	0	0	0	0	0	0	0	0
	Piloerection										
	0	5	5	5	5	5	5	5	5	5	5
	1	0	0	0	0	0	0	0	0	0	0
	2	0	0	0	0	0	0	0	0	0	0
	Arching of the back										
	0	5	5	5	5	5	5	5	5	5	5
	1	0	0	0	0	0	0	0	0	0	0
	2	0	0	0	0	0	0	0	0	0	0
	3	0	0	0	0	0	0	0	0	0	0
	Writhing										
	0	5	5	5	5	5	5	5	5	5	5
	1	0	0	0	0	0	0	0	0	0	0
	2	0	0	0	0	0	0	0	0	0	0
	3	0	0	0	0	0	0	0	0	0	0

Summary of modified Irwin test observation results in male animals before administration (Continued)

Group		S	V	L1	M1	H1	L2	M2	H2	C	P
Number of animals		n=5	n=5	n=5	n=5	n=5	n=5	n=5	n=5	n=5	n=5
Observation inside the observation box	Shivering										
	0	5	5	5	5	5	5	5	5	5	5
	1	0	0	0	0	0	0	0	0	0	0
	2	0	0	0	0	0	0	0	0	0	0
	3	0	0	0	0	0	0	0	0	0	0
	Wet dog shake-like trembling										
	0	5	5	5	5	5	5	5	5	5	5
	1	0	0	0	0	0	0	0	0	0	0
	2	0	0	0	0	0	0	0	0	0	0
	3	0	0	0	0	0	0	0	0	0	0
	Convulsions										
	0	5	5	5	5	5	5	5	5	5	5
	1	0	0	0	0	0	0	0	0	0	0
	Respiration										
	0	0	0	0	0	0	0	0	0	0	0
	1	0	0	0	0	0	0	0	0	0	0
	2	5	5	5	5	5	5	5	5	5	5
	3	0	0	0	0	0	0	0	0	0	0
	Chewing										
	0	5	5	5	5	5	5	5	5	5	5
	1	0	0	0	0	0	0	0	0	0	0
	2	0	0	0	0	0	0	0	0	0	0
	3	0	0	0	0	0	0	0	0	0	0
	Sniffing										
	0	0	0	0	0	0	0	0	0	0	0
	1	0	0	0	0	0	0	0	0	0	0
	2	0	0	0	0	0	0	0	0	0	0
	3	5	5	5	5	5	5	5	5	5	5
	Hind leg spreading										
	0	5	5	5	5	5	5	5	5	5	5
	1	0	0	0	0	0	0	0	0	0	0

Summary of modified Irwin test observation results in male animals before administration (Continued)

Group		S	V	L1	M1	H1	L2	M2	H2	C	P
Number of animals		n=5	n=5	n=5	n=5	n=5	n=5	n=5	n=5	n=5	n=5
Observation inside the observation box	Body posture										
	0	0	0	0	0	0	0	0	0	0	0
	1	0	0	0	0	0	0	0	0	0	0
	2	5	5	5	5	5	5	5	5	5	5
	3	0	0	0	0	0	0	0	0	0	0
	4	0	0	0	0	0	0	0	0	0	0
	Tail position										
	0	0	0	0	0	0	0	0	0	0	0
	1	5	5	5	5	5	5	5	5	5	5
	2	0	0	0	0	0	0	0	0	0	0
	3	0	0	0	0	0	0	0	0	0	0
	Spontaneous activity										
	0	0	0	0	0	0	0	0	0	0	0
	1	0	0	0	0	0	0	0	0	0	0
	2	5	5	5	5	5	5	5	5	5	5
	3	0	0	0	0	0	0	0	0	0	0
	4	0	0	0	0	0	0	0	0	0	0
	Abnormal gait (Ataxia)										
	0	5	5	5	5	5	5	5	5	5	5
	1	0	0	0	0	0	0	0	0	0	0
	2	0	0	0	0	0	0	0	0	0	0
	3	0	0	0	0	0	0	0	0	0	0
	Grooming										
	0	5	5	5	5	5	5	5	5	5	5
	1	0	0	0	0	0	0	0	0	0	0
	2	0	0	0	0	0	0	0	0	0	0
	3	0	0	0	0	0	0	0	0	0	0
	Rearing										
	0	5	5	5	5	5	5	5	5	5	5
	1	0	0	0	0	0	0	0	0	0	0
	2	0	0	0	0	0	0	0	0	0	0
	3	0	0	0	0	0	0	0	0	0	0

Summary of modified Irwin test observation results in male animals before administration (Continued)

Group		S	V	L1	M1	H1	L2	M2	H2	C	P
Number of animals		n=5	n=5	n=5	n=5	n=5	n=5	n=5	n=5	n=5	n=5
Observation inside the observation box	Scratching										
	0	5	5	5	5	5	5	5	5	5	5
	1	0	0	0	0	0	0	0	0	0	0
	2	0	0	0	0	0	0	0	0	0	0
	3	0	0	0	0	0	0	0	0	0	0
	Twitching										
	0	5	5	5	5	5	5	5	5	5	5
	1	0	0	0	0	0	0	0	0	0	0
	2	0	0	0	0	0	0	0	0	0	0
	3	0	0	0	0	0	0	0	0	0	0
	Eyelid closure										
	0	5	5	5	5	5	5	5	5	5	5
	1	0	0	0	0	0	0	0	0	0	0
	2	0	0	0	0	0	0	0	0	0	0
	Urination										
	0	4	1	3	2	3	4	4	1	2	4
	1	1	4	2	3	2	1	1	4	3	1
	Defecation										
	0	2	3	2	3	3	4	2	4	2	2
	1	3	2	3	2	2	1	3	1	3	3
	Death										
	0	5	5	5	5	5	5	5	5	5	5
	1	0	0	0	0	0	0	0	0	0	0
Manipulation inside the observation box	Approach response										
	0	0	0	0	0	0	0	0	0	0	0
	1	0	0	0	0	0	0	0	0	0	0
	2	0	0	0	0	0	0	0	0	0	0
	3	5	5	5	5	5	5	5	5	5	5
	4	0	0	0	0	0	0	0	0	0	0
	5	0	0	0	0	0	0	0	0	0	0

Summary of modified Irwin test observation results in male animals before administration (Continued)

Group		S	V	L1	M1	H1	L2	M2	H2	C	P
Number of animals		n=5	n=5	n=5	n=5	n=5	n=5	n=5	n=5	n=5	n=5
Manipulation inside the observation box	Startle response										
	0	0	0	0	0	0	0	0	0	0	0
	1	0	0	0	0	0	0	0	0	0	0
	2	5	5	5	5	5	5	5	5	5	5
	3	0	0	0	0	0	0	0	0	0	0
	Tail suspension test										
	0	0	0	0	0	0	0	0	0	0	0
	1	0	0	0	0	0	0	0	0	0	0
	2	5	5	5	5	5	5	5	5	5	5
	3	0	0	0	0	0	0	0	0	0	0
	4	0	0	0	0	0	0	0	0	0	0
Manipulation outside the observation box	Vocalization due to handling										
	0	0	0	0	0	0	0	0	0	0	0
	1	5	5	5	5	5	5	5	5	5	5
	2	0	0	0	0	0	0	0	0	0	0
	3	0	0	0	0	0	0	0	0	0	0
	Grid test										
	0	0	0	0	0	0	0	0	0	0	0
	1	0	0	0	0	0	0	0	0	0	0
	2	5	5	5	5	5	5	5	5	5	5
	3	0	0	0	0	0	0	0	0	0	0
	4	0	0	0	0	0	0	0	0	0	0
	Visual orientation										
	0	0	0	0	0	0	0	0	0	0	0
	1	0	0	0	0	0	0	0	0	0	0
	2	5	5	5	5	5	5	5	5	5	5
	Righting reflex										
	0	0	0	0	0	0	0	0	0	0	0
	1	0	0	0	0	0	0	0	0	0	0
	2	5	5	5	5	5	5	5	5	5	5

Summary of modified Irwin test observation results in male animals before administration (Continued)

Group		S	V	L1	M1	H1	L2	M2	H2	C	P
Number of animals		n=5	n=5	n=5	n=5	n=5	n=5	n=5	n=5	n=5	n=5
	Corneal reflex										
	0	0	0	0	0	0	0	0	0	0	0
	1	0	0	0	0	0	0	0	0	0	0
	2	5	5	5	5	5	5	5	5	5	5
	Pinna reflex										
	0	0	0	0	0	0	0	0	0	0	0
	1	0	0	0	0	0	0	0	0	0	0
	2	5	5	5	5	5	5	5	5	5	5
	Grasping reflex										
	0	0	0	0	0	0	0	0	0	0	0
	1	0	0	0	0	0	0	0	0	0	0
	2	5	5	5	5	5	5	5	5	5	5
	Flexor reflex										
	0	0	0	0	0	0	0	0	0	0	0
	1	0	0	0	0	0	0	0	0	0	0
	2	5	5	5	5	5	5	5	5	5	5

Table S2. Summary of modified Irwin test observation results in male animals after 3 minutes of administration

Group		S	V	L1	M1	H1	L2	M2	H2	C	P
Number of animals		n=5	n=5	n=5	n=5	n=5	n=5	n=5	n=5	n=5	n=5
Observation items within the cage	Piloerection										
	0	5	5	5	5	5	5	5	5	5	5
	1	0	0	0	0	0	0	0	0	0	0
	2	0	0	0	0	0	0	0	0	0	0
	Eyelid closure										**
	0	5	5	5	5	5	5	5	5	5	0
	1	0	0	0	0	0	0	0	0	0	5
	2	0	0	0	0	0	0	0	0	0	0
Observation after removal from the cage	Resistance to handling										
	0	5	5	5	5	5	5	5	5	5	5
	1	0	0	0	0	0	0	0	0	0	0
	2	0	0	0	0	0	0	0	0	0	0
	3	0	0	0	0	0	0	0	0	0	0
	Body tension										**
	0	0	0	0	0	0	0	0	0	0	5
	1	5	5	5	5	5	5	5	5	5	0
	2	0	0	0	0	0	0	0	0	0	0
	Skin color										
	0	0	0	0	0	0	0	0	0	0	0
	1	5	5	5	5	5	5	5	5	5	5
	2	0	0	0	0	0	0	0	0	0	0
	Lacrimation										**
	0	5	5	5	5	5	5	5	5	5	0
	1	0	0	0	0	0	0	0	0	0	5
	Salivation										
	0	5	5	5	5	5	5	5	5	5	5
	1	0	0	0	0	0	0	0	0	0	0
	2	0	0	0	0	0	0	0	0	0	0
	3	0	0	0	0	0	0	0	0	0	0
	4	0	0	0	0	0	0	0	0	0	0

S: 5% glucose, 0 mg/kg
V: Liposome, 0 mg/kg
L1: PTX (7.5 mg/kg) -Rg3 (11.25 mg/kg) -lipo
M1: PTX (15 mg/kg) -Rg3 (22.5 mg/kg) -lipo
H1: PTX (30 mg/kg) -Rg3 (45 mg/kg) -lipo
L2: Rg3 (11.25 mg/kg) -lipo
M2: Rg3 (22.5 mg/kg) -lipo
H2: Rg3 (45 mg/kg) -lipo
C: PTX (15 mg/kg) -lipo
P: positive control group, 12 mg/kg chlorpromazine hydrochloride injection

The results are presented as frequencies. **, Compared to the 5% glucose group $P < 0.01$.

Summary of modified Irwin test observation results in male animals after 3 minutes of administration (Continued)

Group		S	V	L1	M1	H1	L2	M2	H2	C	P
Number of animals		n=5	n=5	n=5	n=5	n=5	n=5	n=5	n=5	n=5	n=5
Observation inside the observation box	Awakeness										**
	0	0	0	0	0	0	0	0	0	0	5
	1	0	0	0	0	0	0	0	0	0	0
	2	5	5	5	5	5	5	5	5	5	0
	3	0	0	0	0	0	0	0	0	0	0
	4	0	0	0	0	0	0	0	0	0	0
	Loss of balance										
	0	5	5	5	5	5	5	5	5	5	5
	1	0	0	0	0	0	0	0	0	0	0
	2	0	0	0	0	0	0	0	0	0	0
	3	0	0	0	0	0	0	0	0	0	0
	Paralysis										
	0	5	5	5	5	5	5	5	5	5	5
	1	0	0	0	0	0	0	0	0	0	0
	Exophthalmos										
	0	5	5	5	5	5	5	5	5	5	5
	1	0	0	0	0	0	0	0	0	0	0
	2	0	0	0	0	0	0	0	0	0	0
	3	0	0	0	0	0	0	0	0	0	0
	Piloerection										
	0	5	5	5	5	5	5	5	5	5	5
	1	0	0	0	0	0	0	0	0	0	0
	2	0	0	0	0	0	0	0	0	0	0
	Arching of the back										
	0	5	5	5	5	5	5	5	5	5	5
	1	0	0	0	0	0	0	0	0	0	0
	2	0	0	0	0	0	0	0	0	0	0
	3	0	0	0	0	0	0	0	0	0	0
	Writhing										
	0	5	5	5	5	5	5	5	5	5	5
	1	0	0	0	0	0	0	0	0	0	0
	2	0	0	0	0	0	0	0	0	0	0
	3	0	0	0	0	0	0	0	0	0	0

Summary of modified Irwin test observation results in male animals after 3 minutes of administration (Continued)

Group		S	V	L1	M1	H1	L2	M2	H2	C	P
Number of animals		n=5	n=5	n=5	n=5	n=5	n=5	n=5	n=5	n=5	n=5
Observation inside the observation box	Shivering										
	0	5	5	5	5	5	5	5	5	5	5
	1	0	0	0	0	0	0	0	0	0	0
	2	0	0	0	0	0	0	0	0	0	0
	3	0	0	0	0	0	0	0	0	0	0
	Wet dog shake-like trembling										
	0	5	5	5	5	5	5	5	5	5	5
	1	0	0	0	0	0	0	0	0	0	0
	2	0	0	0	0	0	0	0	0	0	0
	3	0	0	0	0	0	0	0	0	0	0
	Convulsions										
	0	5	5	5	5	5	5	5	5	5	5
	1	0	0	0	0	0	0	0	0	0	0
	Respiration										**
	0	0	0	0	0	0	0	0	0	0	0
	1	0	0	0	0	0	0	0	0	0	5
	2	5	5	5	5	5	5	5	5	5	0
	3	0	0	0	0	0	0	0	0	0	0
	Chewing										
	0	5	5	5	5	5	5	5	5	5	5
	1	0	0	0	0	0	0	0	0	0	0
	2	0	0	0	0	0	0	0	0	0	0
	3	0	0	0	0	0	0	0	0	0	0
	Sniffing										**
	0	0	0	0	0	0	0	0	0	0	5
	1	0	0	0	0	0	0	0	0	0	0
	2	0	0	0	0	0	0	0	0	0	0
	3	5	5	5	5	5	5	5	5	5	0
	Hind leg spreading										
	0	5	5	5	5	5	5	5	5	5	5
	1	0	0	0	0	0	0	0	0	0	0

Summary of modified Irwin test observation results in male animals after 3 minutes of administration (Continued)

Group		S	V	L1	M1	H1	L2	M2	H2	C	P
Number of animals		n=5	n=5	n=5	n=5	n=5	n=5	n=5	n=5	n=5	n=5
Observation inside the observation box	Body posture										**
	0	0	0	0	0	0	0	0	0	0	5
	1	0	0	0	0	0	0	0	0	0	0
	2	5	5	5	5	5	5	5	5	5	0
	3	0	0	0	0	0	0	0	0	0	0
	4	0	0	0	0	0	0	0	0	0	0
	Tail position										
	0	0	0	0	0	0	0	0	0	0	0
	1	5	5	5	5	5	5	5	5	5	5
	2	0	0	0	0	0	0	0	0	0	0
	3	0	0	0	0	0	0	0	0	0	0
	Spontaneous activity										**
	0	0	0	0	0	0	0	0	0	0	5
	1	0	0	0	0	0	0	0	0	0	0
	2	5	5	5	5	5	5	5	5	5	0
	3	0	0	0	0	0	0	0	0	0	0
	4	0	0	0	0	0	0	0	0	0	0
	Abnormal gait (Ataxia)										
	0	5	5	5	5	5	5	5	5	5	5
	1	0	0	0	0	0	0	0	0	0	0
	2	0	0	0	0	0	0	0	0	0	0
	3	0	0	0	0	0	0	0	0	0	0
	Grooming										
	0	5	5	5	5	5	5	5	5	5	5
	1	0	0	0	0	0	0	0	0	0	0
	2	0	0	0	0	0	0	0	0	0	0
	3	0	0	0	0	0	0	0	0	0	0
	Rearing										
	0	5	5	5	5	5	5	5	5	5	5
	1	0	0	0	0	0	0	0	0	0	0
	2	0	0	0	0	0	0	0	0	0	0
	3	0	0	0	0	0	0	0	0	0	0

Summary of modified Irwin test observation results in male animals after 3 minutes of administration (Continued)

Group		S	V	L1	M1	H1	L2	M2	H2	C	P
Number of animals		n=5	n=5	n=5	n=5	n=5	n=5	n=5	n=5	n=5	n=5
Observation inside the observation box	Scratching										
	0	5	5	5	5	5	5	5	5	5	5
	1	0	0	0	0	0	0	0	0	0	0
	2	0	0	0	0	0	0	0	0	0	0
	3	0	0	0	0	0	0	0	0	0	0
	Twitching										
	0	5	5	5	5	5	5	5	5	5	5
	1	0	0	0	0	0	0	0	0	0	0
	2	0	0	0	0	0	0	0	0	0	0
	3	0	0	0	0	0	0	0	0	0	0
	Eyelid closure										**
	0	5	5	5	5	5	5	5	5	5	0
	1	0	0	0	0	0	0	0	0	0	5
	2	0	0	0	0	0	0	0	0	0	0
	Urination										
	0	5	4	3	4	4	5	3	5	5	5
	1	0	1	2	1	1	0	2	0	0	0
	Defecation										
	0	2	5	5	3	4	4	4	3	5	5
	1	3	0	0	2	1	1	1	2	0	0
	Death										
	0	5	5	5	5	5	5	5	5	5	5
	1	0	0	0	0	0	0	0	0	0	0
Manipulation inside the observation box	Approach response										**
	0	0	0	0	0	0	0	0	0	0	5
	1	0	0	0	0	0	0	0	0	0	0
	2	0	0	0	0	0	0	0	0	0	0
	3	5	5	5	5	5	5	5	5	5	0
	4	0	0	0	0	0	0	0	0	0	0
	5	0	0	0	0	0	0	0	0	0	0

Summary of modified Irwin test observation results in male animals after 3 minutes of administration (Continued)

Group		S	V	L1	M1	H1	L2	M2	H2	C	P
Number of animals		n=5	n=5	n=5	n=5	n=5	n=5	n=5	n=5	n=5	n=5
Manipulation inside the observation box	Startle response										**
	0	0	0	0	0	0	0	0	0	0	5
	1	0	0	0	0	0	0	0	0	0	0
	2	5	5	5	5	5	5	5	5	5	0
	3	0	0	0	0	0	0	0	0	0	0
	Tail suspension test										**
	0	0	0	0	0	0	0	0	0	0	0
	1	0	0	0	0	0	0	0	0	0	5
	2	5	5	5	5	5	5	5	5	5	0
	3	0	0	0	0	0	0	0	0	0	0
	4	0	0	0	0	0	0	0	0	0	0
Manipulation outside the observation box	Vocalization due to handling										**
	0	0	0	0	0	0	0	0	0	0	5
	1	5	5	5	5	5	5	5	5	5	0
	2	0	0	0	0	0	0	0	0	0	0
	3	0	0	0	0	0	0	0	0	0	0
	Grid test										**
	0	0	0	0	0	0	0	0	0	0	0
	1	0	0	0	0	0	0	0	0	0	0
	2	5	5	5	5	5	5	5	5	5	0
	3	0	0	0	0	0	0	0	0	0	0
	4	0	0	0	0	0	0	0	0	0	5
	Visual orientation										**
	0	0	0	0	0	0	0	0	0	0	5
	1	0	0	0	0	0	0	0	0	0	0
	2	5	5	5	5	5	5	5	5	5	0
	Righting reflex										**
	0	0	0	0	0	0	0	0	0	0	0
	1	0	0	0	0	0	0	0	0	0	5
	2	5	5	5	5	5	5	5	5	5	0

Summary of modified Irwin test observation results in male animals after 3 minutes of administration (Continued)

Group		S	V	L1	M1	H1	L2	M2	H2	C	P
Number of animals		n=5	n=5	n=5	n=5	n=5	n=5	n=5	n=5	n=5	n=5
	Corneal reflex										**
	0	0	0	0	0	0	0	0	0	0	0
	1	0	0	0	0	0	0	0	0	0	5
	2	5	5	5	5	5	5	5	5	5	0
	Pinna reflex										**
	0	0	0	0	0	0	0	0	0	0	5
	1	0	0	0	0	0	0	0	0	0	0
	2	5	5	5	5	5	5	5	5	5	0
	Grasping reflex										**
	0	0	0	0	0	0	0	0	0	0	0
	1	0	0	0	0	0	0	0	0	0	5
	2	5	5	5	5	5	5	5	5	5	0
	Flexor reflex										**
	0	0	0	0	0	0	0	0	0	0	0
	1	0	0	0	0	0	0	0	0	0	5
	2	5	5	5	5	5	5	5	5	5	0

Table S3. Summary of modified Irwin test observation results in male animals after 1 hour of administration (Continued)

Group		S	V	L1	M1	H1	L2	M2	H2	C	P
Number of animals		n=5	n=5	n=5	n=5	n=5	n=5	n=5	n=5	n=5	n=5
Observation items within the cage	Piloerection										
	0	5	5	5	5	5	5	5	5	5	5
	1	0	0	0	0	0	0	0	0	0	0
	2	0	0	0	0	0	0	0	0	0	0
	Eyelid closure										**
	0	5	5	5	5	5	5	5	5	5	0
	1	0	0	0	0	0	0	0	0	0	5
	2	0	0	0	0	0	0	0	0	0	0
Observation after removal from the cage	Resistance to handling										
	0	5	5	5	5	5	5	5	5	5	5
	1	0	0	0	0	0	0	0	0	0	0
	2	0	0	0	0	0	0	0	0	0	0
	3	0	0	0	0	0	0	0	0	0	0
	Body tension										**
	0	0	0	0	0	0	0	0	0	0	5
	1	5	5	5	5	5	5	5	5	5	0
	2	0	0	0	0	0	0	0	0	0	0
	Skin color										
	0	0	0	0	0	0	0	0	0	0	0
	1	5	5	5	5	5	5	5	5	5	5
	2	0	0	0	0	0	0	0	0	0	0
	Lacrimation										**
	0	5	5	5	5	5	5	5	5	5	0
	1	0	0	0	0	0	0	0	0	0	5
	Salivation										
	0	5	5	5	5	5	5	5	5	5	5
	1	0	0	0	0	0	0	0	0	0	0
	2	0	0	0	0	0	0	0	0	0	0
	3	0	0	0	0	0	0	0	0	0	0
	4	0	0	0	0	0	0	0	0	0	0

S: 5% glucose, 0 mg/kg
V: Liposome, 0 mg/kg
L1: PTX (7.5 mg/kg) -Rg3 (11.25 mg/kg) -lipo
M1: PTX (15 mg/kg) -Rg3 (22.5 mg/kg) -lipo
H1: PTX (30 mg/kg) -Rg3 (45 mg/kg) -lipo
L2: Rg3 (11.25 mg/kg) -lipo
M2: Rg3 (22.5 mg/kg) -lipo
H2: Rg3 (45 mg/kg) -lipo
C: PTX (15 mg/kg) -lipo
P: positive control group, 12 mg/kg chlorpromazine hydrochloride injection

The results are presented as frequencies. **, Compared to the 5% glucose group $P < 0.01$.

Summary of modified Irwin test observation results in male animals after 1 hour of administration (Continued)

Group		S	V	L1	M1	H1	L2	M2	H2	C	P
Number of animals		n=5	n=5	n=5	n=5	n=5	n=5	n=5	n=5	n=5	n=5
Observation inside the observation box	Awakeness										**
	0	0	0	0	0	0	0	0	0	0	5
	1	0	0	0	0	0	0	0	0	0	0
	2	5	5	5	5	5	5	5	5	5	0
	3	0	0	0	0	0	0	0	0	0	0
	4	0	0	0	0	0	0	0	0	0	0
	Loss of balance										
	0	5	5	5	5	5	5	5	5	5	5
	1	0	0	0	0	0	0	0	0	0	0
	2	0	0	0	0	0	0	0	0	0	0
	3	0	0	0	0	0	0	0	0	0	0
	Paralysis										
	0	5	5	5	5	5	5	5	5	5	5
	1	0	0	0	0	0	0	0	0	0	0
	Exophthalmos										
	0	5	5	5	5	5	5	5	5	5	5
	1	0	0	0	0	0	0	0	0	0	0
	2	0	0	0	0	0	0	0	0	0	0
	3	0	0	0	0	0	0	0	0	0	0
	Piloerection										
	0	5	5	5	5	5	5	5	5	5	5
	1	0	0	0	0	0	0	0	0	0	0
	2	0	0	0	0	0	0	0	0	0	0
	Arching of the back										
	0	5	5	5	5	5	5	5	5	5	5
	1	0	0	0	0	0	0	0	0	0	0
	2	0	0	0	0	0	0	0	0	0	0
	3	0	0	0	0	0	0	0	0	0	0
	Writhing										
	0	5	5	5	5	5	5	5	5	5	5
	1	0	0	0	0	0	0	0	0	0	0
	2	0	0	0	0	0	0	0	0	0	0
	3	0	0	0	0	0	0	0	0	0	0

Summary of modified Irwin test observation results in male animals after 1 hour of administration (Continued)

Group		S	V	L1	M1	H1	L2	M2	H2	C	P
Number of animals		n=5	n=5	n=5	n=5	n=5	n=5	n=5	n=5	n=5	n=5
Observation inside the observation box	Shivering										
	0	5	5	5	5	5	5	5	5	5	5
	1	0	0	0	0	0	0	0	0	0	0
	2	0	0	0	0	0	0	0	0	0	0
	3	0	0	0	0	0	0	0	0	0	0
	Wet dog shake-like trembling										
	0	5	5	5	5	5	5	5	5	5	5
	1	0	0	0	0	0	0	0	0	0	0
	2	0	0	0	0	0	0	0	0	0	0
	3	0	0	0	0	0	0	0	0	0	0
	Convulsions										
	0	5	5	5	5	5	5	5	5	5	5
	1	0	0	0	0	0	0	0	0	0	0
	Respiration										**
	0	0	0	0	0	0	0	0	0	0	0
	1	0	0	0	0	0	0	0	0	0	5
	2	5	5	5	5	5	5	5	5	5	0
	3	0	0	0	0	0	0	0	0	0	0
	Chewing										
	0	5	5	5	5	5	5	5	5	5	5
	1	0	0	0	0	0	0	0	0	0	0
	2	0	0	0	0	0	0	0	0	0	0
	3	0	0	0	0	0	0	0	0	0	0
	Sniffing										**
	0	0	0	0	0	0	0	0	0	0	5
	1	0	0	0	0	0	0	0	0	0	0
	2	0	0	0	0	0	0	0	0	0	0
	3	5	5	5	5	5	5	5	5	5	0
	Hind leg spreading										
	0	5	5	5	5	5	5	5	5	5	5
	1	0	0	0	0	0	0	0	0	0	0

Summary of modified Irwin test observation results in male animals after 1 hour of administration (Continued)

Group		S	V	L1	M1	H1	L2	M2	H2	C	P
Number of animals		n=5	n=5	n=5	n=5	n=5	n=5	n=5	n=5	n=5	n=5
Observation inside the observation box	Body posture										**
	0	0	0	0	0	0	0	0	0	0	5
	1	0	0	0	0	0	0	0	0	0	0
	2	5	5	5	5	5	5	5	5	5	0
	3	0	0	0	0	0	0	0	0	0	0
	4	0	0	0	0	0	0	0	0	0	0
	Tail position										
	0	0	0	0	0	0	0	0	0	0	0
	1	5	5	5	5	5	5	5	5	5	5
	2	0	0	0	0	0	0	0	0	0	0
	3	0	0	0	0	0	0	0	0	0	0
	Spontaneous activity										**
	0	0	0	0	0	0	0	0	0	0	5
	1	0	0	0	0	0	0	0	0	0	0
	2	5	5	5	5	5	5	5	5	5	0
	3	0	0	0	0	0	0	0	0	0	0
	4	0	0	0	0	0	0	0	0	0	0
	Abnormal gait (Ataxia)										
	0	5	5	5	5	5	5	5	5	5	5
	1	0	0	0	0	0	0	0	0	0	0
	2	0	0	0	0	0	0	0	0	0	0
	3	0	0	0	0	0	0	0	0	0	0
	Grooming										
	0	5	5	5	5	5	5	5	5	5	5
	1	0	0	0	0	0	0	0	0	0	0
	2	0	0	0	0	0	0	0	0	0	0
	3	0	0	0	0	0	0	0	0	0	0
	Rearing										
	0	5	5	5	5	5	5	5	5	5	5
	1	0	0	0	0	0	0	0	0	0	0
	2	0	0	0	0	0	0	0	0	0	0
	3	0	0	0	0	0	0	0	0	0	0

Summary of modified Irwin test observation results in male animals after 1 hour of administration (Continued)

Group		S	V	L1	M1	H1	L2	M2	H2	C	P
Number of animals		n=5	n=5	n=5	n=5	n=5	n=5	n=5	n=5	n=5	n=5
Observation inside the observation box	Scratching										
	0	5	5	5	5	5	5	5	5	5	5
	1	0	0	0	0	0	0	0	0	0	0
	2	0	0	0	0	0	0	0	0	0	0
	3	0	0	0	0	0	0	0	0	0	0
	Twitching										
	0	5	5	5	5	5	5	5	5	5	5
	1	0	0	0	0	0	0	0	0	0	0
	2	0	0	0	0	0	0	0	0	0	0
	3	0	0	0	0	0	0	0	0	0	0
	Eyelid closure										**
	0	5	5	5	5	5	5	5	5	5	0
	1	0	0	0	0	0	0	0	0	0	5
	2	0	0	0	0	0	0	0	0	0	0
	Urination										
	0	5	5	5	4	4	4	4	2	4	5
	1	0	0	0	1	1	1	1	3	1	0
	Defecation										
	0	1	5	5	5	4	4	4	4	5	5
	1	4	0	0	0	1	1	1	1	0	0
	Death										
	0	5	5	5	5	5	5	5	5	5	5
	1	0	0	0	0	0	0	0	0	0	0
Manipulation inside the observation box	Approach response										**
	0	0	0	0	0	0	0	0	0	0	5
	1	0	0	0	0	0	0	0	0	0	0
	2	0	0	0	0	0	0	0	0	0	0
	3	5	5	5	5	5	5	5	5	5	0
	4	0	0	0	0	0	0	0	0	0	0
	5	0	0	0	0	0	0	0	0	0	0

Summary of modified Irwin test observation results in male animals after 1 hour of administration (Continued)

Group		S	V	L1	M1	H1	L2	M2	H2	C	P
Number of animals		n=5	n=5	n=5	n=5	n=5	n=5	n=5	n=5	n=5	n=5
Manipulation inside the observation box	Startle response										**
	0	0	0	0	0	0	0	0	0	0	5
	1	0	0	0	0	0	0	0	0	0	0
	2	5	5	5	5	5	5	5	5	5	0
	3	0	0	0	0	0	0	0	0	0	0
	Tail suspension test										**
	0	0	0	0	0	0	0	0	0	0	0
	1	0	0	0	0	0	0	0	0	0	5
	2	5	5	5	5	5	5	5	5	5	0
	3	0	0	0	0	0	0	0	0	0	0
	4	0	0	0	0	0	0	0	0	0	0
Manipulation outside the observation box	Vocalization due to handling										**
	0	0	0	0	0	0	0	0	0	0	5
	1	5	5	5	5	5	5	5	5	5	0
	2	0	0	0	0	0	0	0	0	0	0
	3	0	0	0	0	0	0	0	0	0	0
	Grid test										**
	0	0	0	0	0	0	0	0	0	0	0
	1	0	0	0	0	0	0	0	0	0	0
	2	5	5	5	5	5	5	5	5	5	0
	3	0	0	0	0	0	0	0	0	0	0
	4	0	0	0	0	0	0	0	0	0	5
	Visual orientation										**
	0	0	0	0	0	0	0	0	0	0	5
	1	0	0	0	0	0	0	0	0	0	0
	2	5	5	5	5	5	5	5	5	5	0
	Righting reflex										**
	0	0	0	0	0	0	0	0	0	0	0
	1	0	0	0	0	0	0	0	0	0	5
	2	5	5	5	5	5	5	5	5	5	0

Summary of modified Irwin test observation results in male animals after 1 hour of administration (Continued)

Group		S	V	L1	M1	H1	L2	M2	H2	C	P
Number of animals		n=5	n=5	n=5	n=5	n=5	n=5	n=5	n=5	n=5	n=5
	Corneal reflex										**
	0	0	0	0	0	0	0	0	0	0	0
	1	0	0	0	0	0	0	0	0	0	5
	2	5	5	5	5	5	5	5	5	5	0
	Pinna reflex										**
	0	0	0	0	0	0	0	0	0	0	5
	1	0	0	0	0	0	0	0	0	0	0
	2	5	5	5	5	5	5	5	5	5	0
	Grasping reflex										**
	0	0	0	0	0	0	0	0	0	0	0
	1	0	0	0	0	0	0	0	0	0	5
	2	5	5	5	5	5	5	5	5	5	0
	Flexor reflex										**
	0	0	0	0	0	0	0	0	0	0	0
	1	0	0	0	0	0	0	0	0	0	5
	2	5	5	5	5	5	5	5	5	5	0

Table S4. Summary of modified Irwin test observation results in male animals after 4 hours of administration

Group		S	V	L1	M1	H1	L2	M2	H2	C	P
Number of animals		n=5	n=5	n=5	n=5	n=5	n=5	n=5	n=5	n=5	n=5
Observation items within the cage	Piloerection										
	0	5	5	5	5	5	5	5	5	5	5
	1	0	0	0	0	0	0	0	0	0	0
	2	0	0	0	0	0	0	0	0	0	0
	Eyelid closure										**
	0	5	5	5	5	5	5	5	5	5	0
	1	0	0	0	0	0	0	0	0	0	5
	2	0	0	0	0	0	0	0	0	0	0
Observation after removal from the cage	Resistance to handling										
	0	5	5	5	5	5	5	5	5	5	5
	1	0	0	0	0	0	0	0	0	0	0
	2	0	0	0	0	0	0	0	0	0	0
	3	0	0	0	0	0	0	0	0	0	0
	Body tension										**
	0	0	0	0	0	0	0	0	0	0	5
	1	5	5	5	5	5	5	5	5	5	0
	2	0	0	0	0	0	0	0	0	0	0
	Skin color										
	0	0	0	0	0	0	0	0	0	0	0
	1	5	5	5	5	5	5	5	5	5	5
	2	0	0	0	0	0	0	0	0	0	0
	Lacrimation										
	0	5	5	5	5	5	5	5	5	5	5
	1	0	0	0	0	0	0	0	0	0	0
	Salivation										
	0	5	5	5	5	5	5	5	5	5	5
	1	0	0	0	0	0	0	0	0	0	0
	2	0	0	0	0	0	0	0	0	0	0
	3	0	0	0	0	0	0	0	0	0	0
	4	0	0	0	0	0	0	0	0	0	0

S: 5% glucose, 0 mg/kg
V: Liposome, 0 mg/kg
L1: PTX (7.5 mg/kg) -Rg3 (11.25 mg/kg) -lipo
M1: PTX (15 mg/kg) -Rg3 (22.5 mg/kg) -lipo
H1: PTX (30 mg/kg) -Rg3 (45 mg/kg) -lipo
L2: Rg3 (11.25 mg/kg) -lipo
M2: Rg3 (22.5 mg/kg) -lipo
H2: Rg3 (45 mg/kg) -lipo
C: PTX (15 mg/kg) -lipo
P: positive control group, 12 mg/kg chlorpromazine hydrochloride injection

The results are presented as frequencies. **, Compared to the 5% glucose group $P < 0.01$.

Summary of modified Irwin test observation results in male animals after 4 hours of administration (Continued)

Group		S	V	L1	M1	H1	L2	M2	H2	C	P
Number of animals		n=5	n=5	n=5	n=5	n=5	n=5	n=5	n=5	n=5	n=5
Observation inside the observation box	Awakeness										**
	0	0	0	0	0	0	0	0	0	0	5
	1	0	0	0	0	0	0	0	0	0	0
	2	5	5	5	5	5	5	5	5	5	0
	3	0	0	0	0	0	0	0	0	0	0
	4	0	0	0	0	0	0	0	0	0	0
	Loss of balance										
	0	5	5	5	5	5	5	5	5	5	5
	1	0	0	0	0	0	0	0	0	0	0
	2	0	0	0	0	0	0	0	0	0	0
	3	0	0	0	0	0	0	0	0	0	0
	Paralysis										
	0	5	5	5	5	5	5	5	5	5	5
	1	0	0	0	0	0	0	0	0	0	0
	Exophthalmos										
	0	5	5	5	5	5	5	5	5	5	5
	1	0	0	0	0	0	0	0	0	0	0
	2	0	0	0	0	0	0	0	0	0	0
	3	0	0	0	0	0	0	0	0	0	0
	Piloerection										
	0	5	5	5	5	5	5	5	5	5	5
	1	0	0	0	0	0	0	0	0	0	0
	2	0	0	0	0	0	0	0	0	0	0
	Arching of the back										
	0	5	5	5	5	5	5	5	5	5	5
	1	0	0	0	0	0	0	0	0	0	0
	2	0	0	0	0	0	0	0	0	0	0
	3	0	0	0	0	0	0	0	0	0	0
	Writhing										
	0	5	5	5	5	5	5	5	5	5	5
	1	0	0	0	0	0	0	0	0	0	0
	2	0	0	0	0	0	0	0	0	0	0
	3	0	0	0	0	0	0	0	0	0	0

Summary of modified Irwin test observation results in male animals after 4 hours of administration (Continued)

Group		S	V	L1	M1	H1	L2	M2	H2	C	P
Number of animals		n=5	n=5	n=5	n=5	n=5	n=5	n=5	n=5	n=5	n=5
Observation inside the observation box	Shivering										
	0	5	5	5	5	5	5	5	5	5	5
	1	0	0	0	0	0	0	0	0	0	0
	2	0	0	0	0	0	0	0	0	0	0
	3	0	0	0	0	0	0	0	0	0	0
	Wet dog shake-like trembling										
	0	5	5	5	5	5	5	5	5	5	5
	1	0	0	0	0	0	0	0	0	0	0
	2	0	0	0	0	0	0	0	0	0	0
	3	0	0	0	0	0	0	0	0	0	0
	Convulsions										
	0	5	5	5	5	5	5	5	5	5	5
	1	0	0	0	0	0	0	0	0	0	0
	Respiration										**
	0	0	0	0	0	0	0	0	0	0	0
	1	0	0	0	0	0	0	0	0	0	5
	2	5	5	5	5	5	5	5	5	5	0
	3	0	0	0	0	0	0	0	0	0	0
	Chewing										
	0	5	5	5	5	5	5	5	5	5	5
	1	0	0	0	0	0	0	0	0	0	0
	2	0	0	0	0	0	0	0	0	0	0
	3	0	0	0	0	0	0	0	0	0	0
	Sniffing										**
	0	0	0	0	0	0	0	0	0	0	5
	1	0	0	0	0	0	0	0	0	0	0
	2	0	0	0	0	0	0	0	0	0	0
	3	5	5	5	5	5	5	5	5	5	0
	Hind leg spreading										
	0	5	5	5	5	5	5	5	5	5	5
	1	0	0	0	0	0	0	0	0	0	0

Summary of modified Irwin test observation results in male animals after 4 hours of administration (Continued)

Group		S	V	L1	M1	H1	L2	M2	H2	C	P
Number of animals		n=5	n=5	n=5	n=5	n=5	n=5	n=5	n=5	n=5	n=5
Observation inside the observation box	Body posture										**
	0	0	0	0	0	0	0	0	0	0	5
	1	0	0	0	0	0	0	0	0	0	0
	2	5	5	5	5	5	5	5	5	5	0
	3	0	0	0	0	0	0	0	0	0	0
	4	0	0	0	0	0	0	0	0	0	0
	Tail position										
	0	0	0	0	0	0	0	0	0	0	0
	1	5	5	5	5	5	5	5	5	5	5
	2	0	0	0	0	0	0	0	0	0	0
	3	0	0	0	0	0	0	0	0	0	0
	Spontaneous activity										**
	0	0	0	0	0	0	0	0	0	0	5
	1	0	0	0	0	0	0	0	0	0	0
	2	5	5	5	5	5	5	5	5	5	0
	3	0	0	0	0	0	0	0	0	0	0
	4	0	0	0	0	0	0	0	0	0	0
	Abnormal gait (Ataxia)										
	0	5	5	5	5	5	5	5	5	5	5
	1	0	0	0	0	0	0	0	0	0	0
	2	0	0	0	0	0	0	0	0	0	0
	3	0	0	0	0	0	0	0	0	0	0
	Grooming										
	0	5	5	5	5	5	5	5	5	5	5
	1	0	0	0	0	0	0	0	0	0	0
	2	0	0	0	0	0	0	0	0	0	0
	3	0	0	0	0	0	0	0	0	0	0
	Rearing										
	0	5	5	5	5	5	5	5	5	5	5
	1	0	0	0	0	0	0	0	0	0	0
	2	0	0	0	0	0	0	0	0	0	0
	3	0	0	0	0	0	0	0	0	0	0

Summary of modified Irwin test observation results in male animals after 4 hours of administration (Continued)

Group		S	V	L1	M1	H1	L2	M2	H2	C	P
Number of animals		n=5	n=5	n=5	n=5	n=5	n=5	n=5	n=5	n=5	n=5
Observation inside the observation box	Scratching										
	0	5	5	5	5	5	5	5	5	5	5
	1	0	0	0	0	0	0	0	0	0	0
	2	0	0	0	0	0	0	0	0	0	0
	3	0	0	0	0	0	0	0	0	0	0
	Twitching										
	0	5	5	5	5	5	5	5	5	5	5
	1	0	0	0	0	0	0	0	0	0	0
	2	0	0	0	0	0	0	0	0	0	0
	3	0	0	0	0	0	0	0	0	0	0
	Eyelid closure										**
	0	5	5	5	5	5	5	5	5	5	0
	1	0	0	0	0	0	0	0	0	0	5
	2	0	0	0	0	0	0	0	0	0	0
	Urination										
	0	2	4	2	3	4	3	3	3	3	5
	1	3	1	3	2	1	2	2	2	2	0
	Defecation										
	0	2	3	2	3	3	3	2	2	4	5
	1	3	2	3	2	2	2	3	3	1	0
	Death										
	0	5	5	5	5	5	5	5	5	5	5
	1	0	0	0	0	0	0	0	0	0	0
Manipulation inside the observation box	Approach response										**
	0	0	0	0	0	0	0	0	0	0	0
	1	0	0	0	0	0	0	0	0	0	5
	2	0	0	0	0	0	0	0	0	0	0
	3	5	5	5	5	5	5	5	5	5	0
	4	0	0	0	0	0	0	0	0	0	0
	5	0	0	0	0	0	0	0	0	0	0

Summary of modified Irwin test observation results in male animals after 4 hours of administration (Continued)

Group		S	V	L1	M1	H1	L2	M2	H2	C	P
Number of animals		n=5	n=5	n=5	n=5	n=5	n=5	n=5	n=5	n=5	n=5
Manipulation inside the observation box	Startle response										**
	0	0	0	0	0	0	0	0	0	0	0
	1	0	0	0	0	0	0	0	0	0	5
	2	5	5	5	5	5	5	5	5	5	0
	3	0	0	0	0	0	0	0	0	0	0
	Tail suspension test										**
	0	0	0	0	0	0	0	0	0	0	0
	1	0	0	0	0	0	0	0	0	0	5
	2	5	5	5	5	5	5	5	5	5	0
	3	0	0	0	0	0	0	0	0	0	0
	4	0	0	0	0	0	0	0	0	0	0
Manipulation outside the observation box	Vocalization due to handling										**
	0	0	0	0	0	0	0	0	0	0	5
	1	5	5	5	5	5	5	5	5	5	0
	2	0	0	0	0	0	0	0	0	0	0
	3	0	0	0	0	0	0	0	0	0	0
	Grid test										**
	0	0	0	0	0	0	0	0	0	0	0
	1	0	0	0	0	0	0	0	0	0	0
	2	5	5	5	5	5	5	5	5	5	0
	3	0	0	0	0	0	0	0	0	0	0
	4	0	0	0	0	0	0	0	0	0	5
	Visual orientation										
	0	0	0	0	0	0	0	0	0	0	0
	1	0	0	0	0	0	0	0	0	0	0
	2	5	5	5	5	5	5	5	5	5	5
	Righting reflex										**
	0	0	0	0	0	0	0	0	0	0	0
	1	0	0	0	0	0	0	0	0	0	5
	2	5	5	5	5	5	5	5	5	5	0

Summary of modified Irwin test observation results in male animals after 4 hours of administration (Continued)

Group		S	V	L1	M1	H1	L2	M2	H2	C	P
Number of animals		n=5	n=5	n=5	n=5	n=5	n=5	n=5	n=5	n=5	n=5
	Corneal reflex										**
	0	0	0	0	0	0	0	0	0	0	0
	1	0	0	0	0	0	0	0	0	0	5
	2	5	5	5	5	5	5	5	5	5	0
	Pinna reflex										**
	0	0	0	0	0	0	0	0	0	0	0
	1	0	0	0	0	0	0	0	0	0	5
	2	5	5	5	5	5	5	5	5	5	0
	Grasping reflex										**
	0	0	0	0	0	0	0	0	0	0	0
	1	0	0	0	0	0	0	0	0	0	5
	2	5	5	5	5	5	5	5	5	5	0
	Flexor reflex										**
	0	0	0	0	0	0	0	0	0	0	0
	1	0	0	0	0	0	0	0	0	0	5
	2	5	5	5	5	5	5	5	5	5	0

Table S5. Summary of modified Irwin test observation results in male animals after 24 hours of administration

Group		S	V	L1	M1	H1	L2	M2	H2	C	P
Number of animals		n=5	n=5	n=5	n=5	n=5	n=5	n=5	n=5	n=5	n=5
Observation items within the cage	Piloerection										
	0	5	5	5	5	5	5	5	5	5	5
	1	0	0	0	0	0	0	0	0	0	0
	2	0	0	0	0	0	0	0	0	0	0
	Eyelid closure										
	0	5	5	5	5	5	5	5	5	5	5
	1	0	0	0	0	0	0	0	0	0	0
	2	0	0	0	0	0	0	0	0	0	0
Observation after removal from the cage	Resistance to handling										
	0	5	5	5	5	5	5	5	5	5	5
	1	0	0	0	0	0	0	0	0	0	0
	2	0	0	0	0	0	0	0	0	0	0
	3	0	0	0	0	0	0	0	0	0	0
	Body tension										
	0	0	0	0	0	0	0	0	0	0	0
	1	5	5	5	5	5	5	5	5	5	5
	2	0	0	0	0	0	0	0	0	0	0
	Skin color										
	0	0	0	0	0	0	0	0	0	0	0
	1	5	5	5	5	5	5	5	5	5	5
	2	0	0	0	0	0	0	0	0	0	0
	Lacrimation										
	0	5	5	5	5	5	5	5	5	5	5
	1	0	0	0	0	0	0	0	0	0	0
	Salivation										
	0	5	5	5	5	5	5	5	5	5	5
	1	0	0	0	0	0	0	0	0	0	0
	2	0	0	0	0	0	0	0	0	0	0
	3	0	0	0	0	0	0	0	0	0	0
	4	0	0	0	0	0	0	0	0	0	0

S: 5% glucose, 0 mg/kg
V: Liposome, 0 mg/kg
L1: PTX (7.5 mg/kg) -Rg3 (11.25 mg/kg) -lipo
M1: PTX (15 mg/kg) -Rg3 (22.5 mg/kg) -lipo
H1: PTX (30 mg/kg) -Rg3 (45 mg/kg) -lipo
L2: Rg3 (11.25 mg/kg) -lipo
M2: Rg3 (22.5 mg/kg) -lipo
H2: Rg3 (45 mg/kg) -lipo
C: PTX (15 mg/kg) -lipo
P: positive control group, 12 mg/kg chlorpromazine hydrochloride injection

The results are presented as frequencies. Compared to the 5% glucose group, $P > 0.05$.

Summary of modified Irwin test observation results in male animals after 24 hours of administration (Continued)

Group		S	V	L1	M1	H1	L2	M2	H2	C	P
Number of animals		n=5	n=5	n=5	n=5	n=5	n=5	n=5	n=5	n=5	n=5
Observation inside the observation box	Awakeness										
	0	0	0	0	0	0	0	0	0	0	0
	1	0	0	0	0	0	0	0	0	0	0
	2	5	5	5	5	5	5	5	5	5	5
	3	0	0	0	0	0	0	0	0	0	0
	4	0	0	0	0	0	0	0	0	0	0
	Loss of balance										
	0	5	5	5	5	5	5	5	5	5	5
	1	0	0	0	0	0	0	0	0	0	0
	2	0	0	0	0	0	0	0	0	0	0
	3	0	0	0	0	0	0	0	0	0	0
	Paralysis										
	0	5	5	5	5	5	5	5	5	5	5
	1	0	0	0	0	0	0	0	0	0	0
	Exophthalmos										
	0	5	5	5	5	5	5	5	5	5	5
	1	0	0	0	0	0	0	0	0	0	0
	2	0	0	0	0	0	0	0	0	0	0
	3	0	0	0	0	0	0	0	0	0	0
	Piloerection										
	0	5	5	5	5	5	5	5	5	5	5
	1	0	0	0	0	0	0	0	0	0	0
	2	0	0	0	0	0	0	0	0	0	0
	Arching of the back										
	0	5	5	5	5	5	5	5	5	5	5
	1	0	0	0	0	0	0	0	0	0	0
	2	0	0	0	0	0	0	0	0	0	0
	3	0	0	0	0	0	0	0	0	0	0
	Writhing										
	0	5	5	5	5	5	5	5	5	5	5
	1	0	0	0	0	0	0	0	0	0	0
	2	0	0	0	0	0	0	0	0	0	0
	3	0	0	0	0	0	0	0	0	0	0

Summary of modified Irwin test observation results in male animals after 24 hours of administration (Continued)

Group		S	V	L1	M1	H1	L2	M2	H2	C	P
Number of animals		n=5	n=5	n=5	n=5	n=5	n=5	n=5	n=5	n=5	n=5
Observation inside the observation box	Shivering										
	0	5	5	5	5	5	5	5	5	5	5
	1	0	0	0	0	0	0	0	0	0	0
	2	0	0	0	0	0	0	0	0	0	0
	3	0	0	0	0	0	0	0	0	0	0
	Wet dog shake-like trembling										
	0	5	5	5	5	5	5	5	5	5	5
	1	0	0	0	0	0	0	0	0	0	0
	2	0	0	0	0	0	0	0	0	0	0
	3	0	0	0	0	0	0	0	0	0	0
	Convulsions										
	0	5	5	5	5	5	5	5	5	5	5
	1	0	0	0	0	0	0	0	0	0	0
	Respiration										
	0	0	0	0	0	0	0	0	0	0	0
	1	0	0	0	0	0	0	0	0	0	0
	2	5	5	5	5	5	5	5	5	5	5
	3	0	0	0	0	0	0	0	0	0	0
	Chewing										
	0	5	5	5	5	5	5	5	5	5	5
	1	0	0	0	0	0	0	0	0	0	0
	2	0	0	0	0	0	0	0	0	0	0
	3	0	0	0	0	0	0	0	0	0	0
	Sniffing										
	0	0	0	0	0	0	0	0	0	0	0
	1	0	0	0	0	0	0	0	0	0	0
	2	0	0	0	0	0	0	0	0	0	0
	3	5	5	5	5	5	5	5	5	5	5
	Hind leg spreading										
	0	5	5	5	5	5	5	5	5	5	5
	1	0	0	0	0	0	0	0	0	0	0

Summary of modified Irwin test observation results in male animals after 24 hours of administration (Continued)

Group		S	V	L1	M1	H1	L2	M2	H2	C	P
Number of animals		n=5	n=5	n=5	n=5	n=5	n=5	n=5	n=5	n=5	n=5
Observation inside the observation box	Body posture										
	0	0	0	0	0	0	0	0	0	0	0
	1	0	0	0	0	0	0	0	0	0	0
	2	5	5	5	5	5	5	5	5	5	5
	3	0	0	0	0	0	0	0	0	0	0
	4	0	0	0	0	0	0	0	0	0	0
	Tail position										
	0	0	0	0	0	0	0	0	0	0	0
	1	5	5	5	5	5	5	5	5	5	5
	2	0	0	0	0	0	0	0	0	0	0
	3	0	0	0	0	0	0	0	0	0	0
	Spontaneous activity										
	0	0	0	0	0	0	0	0	0	0	0
	1	0	0	0	0	0	0	0	0	0	0
	2	5	5	5	5	5	5	5	5	5	5
	3	0	0	0	0	0	0	0	0	0	0
	4	0	0	0	0	0	0	0	0	0	0
	Abnormal gait (Ataxia)										
	0	5	5	5	5	5	5	5	5	5	5
	1	0	0	0	0	0	0	0	0	0	0
	2	0	0	0	0	0	0	0	0	0	0
	3	0	0	0	0	0	0	0	0	0	0
	Grooming										
	0	5	5	5	5	5	5	5	5	5	5
	1	0	0	0	0	0	0	0	0	0	0
	2	0	0	0	0	0	0	0	0	0	0
	3	0	0	0	0	0	0	0	0	0	0
	Rearing										
	0	5	5	5	5	5	5	5	5	5	5
	1	0	0	0	0	0	0	0	0	0	0
	2	0	0	0	0	0	0	0	0	0	0
	3	0	0	0	0	0	0	0	0	0	0

Summary of modified Irwin test observation results in male animals after 24 hours of administration (Continued)

Group		S	V	L1	M1	H1	L2	M2	H2	C	P
Number of animals		n=5	n=5	n=5	n=5	n=5	n=5	n=5	n=5	n=5	n=5
Observation inside the observation box	Scratching										
	0	5	5	5	5	5	5	5	5	5	5
	1	0	0	0	0	0	0	0	0	0	0
	2	0	0	0	0	0	0	0	0	0	0
	3	0	0	0	0	0	0	0	0	0	0
	Twitching										
	0	5	5	5	5	5	5	5	5	5	5
	1	0	0	0	0	0	0	0	0	0	0
	2	0	0	0	0	0	0	0	0	0	0
	3	0	0	0	0	0	0	0	0	0	0
	Eyelid closure										
	0	5	5	5	5	5	5	5	5	5	5
	1	0	0	0	0	0	0	0	0	0	0
	2	0	0	0	0	0	0	0	0	0	0
	Urination										
	0	3	4	2	3	3	4	3	3	3	1
	1	2	1	3	2	2	1	2	2	2	4
	Defecation										
	0	3	3	2	3	4	1	2	1	1	3
	1	2	2	3	2	1	4	3	4	4	2
	Death										
	0	5	5	5	5	5	5	5	5	5	5
	1	0	0	0	0	0	0	0	0	0	0
Manipulation inside the observation box	Approach response										
	0	0	0	0	0	0	0	0	0	0	0
	1	0	0	0	0	0	0	0	0	0	0
	2	0	0	0	0	0	0	0	0	0	0
	3	5	5	5	5	5	5	5	5	5	5
	4	0	0	0	0	0	0	0	0	0	0
	5	0	0	0	0	0	0	0	0	0	0

Summary of modified Irwin test observation results in male animals after 24 hours of administration (Continued)

Group		S	V	L1	M1	H1	L2	M2	H2	C	P
Number of animals		n=5	n=5	n=5	n=5	n=5	n=5	n=5	n=5	n=5	n=5
Manipulation inside the observation box	Startle response										
	0	0	0	0	0	0	0	0	0	0	0
	1	0	0	0	0	0	0	0	0	0	0
	2	5	5	5	5	5	5	5	5	5	5
	3	0	0	0	0	0	0	0	0	0	0
	Tail suspension test										
	0	0	0	0	0	0	0	0	0	0	0
	1	0	0	0	0	0	0	0	0	0	0
	2	5	5	5	5	5	5	5	5	5	5
	3	0	0	0	0	0	0	0	0	0	0
	4	0	0	0	0	0	0	0	0	0	0
Manipulation outside the observation box	Vocalization due to handling										
	0	0	0	0	0	0	0	0	0	0	0
	1	5	5	5	5	5	5	5	5	5	5
	2	0	0	0	0	0	0	0	0	0	0
	3	0	0	0	0	0	0	0	0	0	0
	Grid test										
	0	0	0	0	0	0	0	0	0	0	0
	1	0	0	0	0	0	0	0	0	0	0
	2	5	5	5	5	5	5	5	5	5	5
	3	0	0	0	0	0	0	0	0	0	0
	4	0	0	0	0	0	0	0	0	0	0
	Visual orientation										
	0	0	0	0	0	0	0	0	0	0	0
	1	0	0	0	0	0	0	0	0	0	0
	2	5	5	5	5	5	5	5	5	5	5
	Righting reflex										
	0	0	0	0	0	0	0	0	0	0	0
	1	0	0	0	0	0	0	0	0	0	0
	2	5	5	5	5	5	5	5	5	5	5

Summary of modified Irwin test observation results in male animals after 24 hours of administration (Continued)

Group		S	V	L1	M1	H1	L2	M2	H2	C	P
Number of animals		n=5	n=5	n=5	n=5	n=5	n=5	n=5	n=5	n=5	n=5
	Corneal reflex										
	0	0	0	0	0	0	0	0	0	0	0
	1	0	0	0	0	0	0	0	0	0	0
	2	5	5	5	5	5	5	5	5	5	5
	Pinna reflex										
	0	0	0	0	0	0	0	0	0	0	0
	1	0	0	0	0	0	0	0	0	0	0
	2	5	5	5	5	5	5	5	5	5	5
	Grasping reflex										
	0	0	0	0	0	0	0	0	0	0	0
	1	0	0	0	0	0	0	0	0	0	0
	2	5	5	5	5	5	5	5	5	5	5
	Flexor reflex										
	0	0	0	0	0	0	0	0	0	0	0
	1	0	0	0	0	0	0	0	0	0	0
	2	5	5	5	5	5	5	5	5	5	5

Table S6. Summary of modified Irwin test observation results in male animals after 72 hours of administration

Group		S	V	L1	M1	H1	L2	M2	H2	C	P
Number of animals		n=5	n=5	n=5	n=5	n=5	n=5	n=5	n=5	n=5	n=5
Observation items within the cage	Piloerection										
	0	5	5	5	5	5	5	5	5	5	5
	1	0	0	0	0	0	0	0	0	0	0
	2	0	0	0	0	0	0	0	0	0	0
	Eyelid closure										
	0	5	5	5	5	5	5	5	5	5	5
	1	0	0	0	0	0	0	0	0	0	0
	2	0	0	0	0	0	0	0	0	0	0
Observation after removal from the cage	Resistance to handling										
	0	5	5	5	5	5	5	5	5	5	5
	1	0	0	0	0	0	0	0	0	0	0
	2	0	0	0	0	0	0	0	0	0	0
	3	0	0	0	0	0	0	0	0	0	0
	Body tension										
	0	0	0	0	0	0	0	0	0	0	0
	1	5	5	5	5	5	5	5	5	5	5
	2	0	0	0	0	0	0	0	0	0	0
	Skin color										
	0	0	0	0	0	0	0	0	0	0	0
	1	5	5	5	5	5	5	5	5	5	5
	2	0	0	0	0	0	0	0	0	0	0
	Lacrimation										
	0	5	5	5	5	5	5	5	5	5	5
	1	0	0	0	0	0	0	0	0	0	0
	Salivation										
	0	5	5	5	5	5	5	5	5	5	5
	1	0	0	0	0	0	0	0	0	0	0
	2	0	0	0	0	0	0	0	0	0	0
	3	0	0	0	0	0	0	0	0	0	0
	4	0	0	0	0	0	0	0	0	0	0

S: 5% glucose, 0 mg/kg
V: Liposome, 0 mg/kg
L1: PTX (7.5 mg/kg) -Rg3 (11.25 mg/kg) -lipo
M1: PTX (15 mg/kg) -Rg3 (22.5 mg/kg) -lipo
H1: PTX (30 mg/kg) -Rg3 (45 mg/kg) -lipo
L2: Rg3 (11.25 mg/kg) -lipo
M2: Rg3 (22.5 mg/kg) -lipo
H2: Rg3 (45 mg/kg) -lipo
C: PTX (15 mg/kg) -lipo
P: positive control group, 12 mg/kg chlorpromazine hydrochloride injection
The results are presented as frequencies. Compared to the 5% glucose group, $P > 0.05$.

Summary of modified Irwin test observation results in male animals after 72 hours of administration (Continued)

Group		S	V	L1	M1	H1	L2	M2	H2	C	P
Number of animals		n=5	n=5	n=5	n=5	n=5	n=5	n=5	n=5	n=5	n=5
Observation inside the observation box	Awakeness										
	0	0	0	0	0	0	0	0	0	0	0
	1	0	0	0	0	0	0	0	0	0	0
	2	5	5	5	5	5	5	5	5	5	5
	3	0	0	0	0	0	0	0	0	0	0
	4	0	0	0	0	0	0	0	0	0	0
	Loss of balance										
	0	5	5	5	5	5	5	5	5	5	5
	1	0	0	0	0	0	0	0	0	0	0
	2	0	0	0	0	0	0	0	0	0	0
	3	0	0	0	0	0	0	0	0	0	0
	Paralysis										
	0	5	5	5	5	5	5	5	5	5	5
	1	0	0	0	0	0	0	0	0	0	0
	Exophthalmos										
	0	5	5	5	5	5	5	5	5	5	5
	1	0	0	0	0	0	0	0	0	0	0
	2	0	0	0	0	0	0	0	0	0	0
	3	0	0	0	0	0	0	0	0	0	0
	Piloerection										
	0	5	5	5	5	5	5	5	5	5	5
	1	0	0	0	0	0	0	0	0	0	0
	2	0	0	0	0	0	0	0	0	0	0
	Arching of the back										
	0	5	5	5	5	5	5	5	5	5	5
	1	0	0	0	0	0	0	0	0	0	0
	2	0	0	0	0	0	0	0	0	0	0
	3	0	0	0	0	0	0	0	0	0	0
	Writhing										
	0	5	5	5	5	5	5	5	5	5	5
	1	0	0	0	0	0	0	0	0	0	0
	2	0	0	0	0	0	0	0	0	0	0
	3	0	0	0	0	0	0	0	0	0	0

Summary of modified Irwin test observation results in male animals after 72 hours of administration (Continued)

Group		S	V	L1	M1	H1	L2	M2	H2	C	P
Number of animals		n=5	n=5	n=5	n=5	n=5	n=5	n=5	n=5	n=5	n=5
Observation inside the observation box	Shivering										
	0	5	5	5	5	5	5	5	5	5	5
	1	0	0	0	0	0	0	0	0	0	0
	2	0	0	0	0	0	0	0	0	0	0
	3	0	0	0	0	0	0	0	0	0	0
	Wet dog shake-like trembling										
	0	5	5	5	5	5	5	5	5	5	5
	1	0	0	0	0	0	0	0	0	0	0
	2	0	0	0	0	0	0	0	0	0	0
	3	0	0	0	0	0	0	0	0	0	0
	Convulsions										
	0	5	5	5	5	5	5	5	5	5	5
	1	0	0	0	0	0	0	0	0	0	0
	Respiration										
	0	0	0	0	0	0	0	0	0	0	0
	1	0	0	0	0	0	0	0	0	0	0
	2	5	5	5	5	5	5	5	5	5	5
	3	0	0	0	0	0	0	0	0	0	0
	Chewing										
	0	5	5	5	5	5	5	5	5	5	5
	1	0	0	0	0	0	0	0	0	0	0
	2	0	0	0	0	0	0	0	0	0	0
	3	0	0	0	0	0	0	0	0	0	0
	Sniffing										
	0	0	0	0	0	0	0	0	0	0	0
	1	0	0	0	0	0	0	0	0	0	0
	2	0	0	0	0	0	0	0	0	0	0
	3	5	5	5	5	5	5	5	5	5	5
	Hind leg spreading										
	0	5	5	5	5	5	5	5	5	5	5
	1	0	0	0	0	0	0	0	0	0	0

Summary of modified Irwin test observation results in male animals after 72 hours of administration (Continued)

Group		S	V	L1	M1	H1	L2	M2	H2	C	P
Number of animals		n=5	n=5	n=5	n=5	n=5	n=5	n=5	n=5	n=5	n=5
Observation inside the observation box	Body posture										
	0	0	0	0	0	0	0	0	0	0	0
	1	0	0	0	0	0	0	0	0	0	0
	2	5	5	5	5	5	5	5	5	5	5
	3	0	0	0	0	0	0	0	0	0	0
	4	0	0	0	0	0	0	0	0	0	0
	Tail position										
	0	0	0	0	0	0	0	0	0	0	0
	1	5	5	5	5	5	5	5	5	5	5
	2	0	0	0	0	0	0	0	0	0	0
	3	0	0	0	0	0	0	0	0	0	0
	Spontaneous activity										
	0	0	0	0	0	0	0	0	0	0	0
	1	0	0	0	0	0	0	0	0	0	0
	2	5	5	5	5	5	5	5	5	5	5
	3	0	0	0	0	0	0	0	0	0	0
	4	0	0	0	0	0	0	0	0	0	0
	Abnormal gait (Ataxia)										
	0	5	5	5	5	5	5	5	5	5	5
	1	0	0	0	0	0	0	0	0	0	0
	2	0	0	0	0	0	0	0	0	0	0
	3	0	0	0	0	0	0	0	0	0	0
	Grooming										
	0	5	5	5	5	5	5	5	5	5	5
	1	0	0	0	0	0	0	0	0	0	0
	2	0	0	0	0	0	0	0	0	0	0
	3	0	0	0	0	0	0	0	0	0	0
	Rearing										
	0	5	5	5	5	5	5	5	5	5	5
	1	0	0	0	0	0	0	0	0	0	0
	2	0	0	0	0	0	0	0	0	0	0
	3	0	0	0	0	0	0	0	0	0	0

Summary of modified Irwin test observation results in male animals after 72 hours of administration (Continued)

Group		S	V	L1	M1	H1	L2	M2	H2	C	P
Number of animals		n=5	n=5	n=5	n=5	n=5	n=5	n=5	n=5	n=5	n=5
Observation inside the observation box	Scratching										
	0	5	5	5	5	5	5	5	5	5	5
	1	0	0	0	0	0	0	0	0	0	0
	2	0	0	0	0	0	0	0	0	0	0
	3	0	0	0	0	0	0	0	0	0	0
	Twitching										
	0	5	5	5	5	5	5	5	5	5	5
	1	0	0	0	0	0	0	0	0	0	0
	2	0	0	0	0	0	0	0	0	0	0
	3	0	0	0	0	0	0	0	0	0	0
	Eyelid closure										
	0	5	5	5	5	5	5	5	5	5	5
	1	0	0	0	0	0	0	0	0	0	0
	2	0	0	0	0	0	0	0	0	0	0
	Urination										
	0	5	5	5	5	5	5	5	5	5	5
	1	0	0	0	0	0	0	0	0	0	0
	Defecation										
	0	3	4	5	4	4	4	3	5	4	5
	1	2	1	0	1	1	1	2	0	1	0
	Death										
	0	5	5	5	5	5	5	5	5	5	5
	1	0	0	0	0	0	0	0	0	0	0
Manipulation inside the observation box	Approach response										
	0	0	0	0	0	0	0	0	0	0	0
	1	0	0	0	0	0	0	0	0	0	0
	2	0	0	0	0	0	0	0	0	0	0
	3	5	5	5	5	5	5	5	5	5	5
	4	0	0	0	0	0	0	0	0	0	0
	5	0	0	0	0	0	0	0	0	0	0

Summary of modified Irwin test observation results in male animals after 72 hours of administration (Continued)

Group		S	V	L1	M1	H1	L2	M2	H2	C	P
Number of animals		n=5	n=5	n=5	n=5	n=5	n=5	n=5	n=5	n=5	n=5
Manipulation inside the observation box	Startle response										
	0	0	0	0	0	0	0	0	0	0	0
	1	0	0	0	0	0	0	0	0	0	0
	2	5	5	5	5	5	5	5	5	5	5
	3	0	0	0	0	0	0	0	0	0	0
	Tail suspension test										
	0	0	0	0	0	0	0	0	0	0	0
	1	0	0	0	0	0	0	0	0	0	0
	2	5	5	5	5	5	5	5	5	5	5
	3	0	0	0	0	0	0	0	0	0	0
	4	0	0	0	0	0	0	0	0	0	0
Manipulation outside the observation box	Vocalization due to handling										
	0	0	0	0	0	0	0	0	0	0	0
	1	5	5	5	5	5	5	5	5	5	5
	2	0	0	0	0	0	0	0	0	0	0
	3	0	0	0	0	0	0	0	0	0	0
	Grid test										
	0	0	0	0	0	0	0	0	0	0	0
	1	0	0	0	0	0	0	0	0	0	0
	2	5	5	5	5	5	5	5	5	5	5
	3	0	0	0	0	0	0	0	0	0	0
	4	0	0	0	0	0	0	0	0	0	0
	Visual orientation										
	0	0	0	0	0	0	0	0	0	0	0
	1	0	0	0	0	0	0	0	0	0	0
	2	5	5	5	5	5	5	5	5	5	5
	Righting reflex										
	0	0	0	0	0	0	0	0	0	0	0
	1	0	0	0	0	0	0	0	0	0	0
	2	5	5	5	5	5	5	5	5	5	5

Summary of modified Irwin test observation results in male animals after 72 hours of administration (Continued)

Group		S	V	L1	M1	H1	L2	M2	H2	C	P
Number of animals		n=5	n=5	n=5	n=5	n=5	n=5	n=5	n=5	n=5	n=5
	Corneal reflex										
	0	0	0	0	0	0	0	0	0	0	0
	1	0	0	0	0	0	0	0	0	0	0
	2	5	5	5	5	5	5	5	5	5	5
	Pinna reflex										
	0	0	0	0	0	0	0	0	0	0	0
	1	0	0	0	0	0	0	0	0	0	0
	2	5	5	5	5	5	5	5	5	5	5
	Grasping reflex										
	0	0	0	0	0	0	0	0	0	0	0
	1	0	0	0	0	0	0	0	0	0	0
	2	5	5	5	5	5	5	5	5	5	5
	Flexor reflex										
	0	0	0	0	0	0	0	0	0	0	0
	1	0	0	0	0	0	0	0	0	0	0
	2	5	5	5	5	5	5	5	5	5	5

Table S7. Summary of modified Irwin test observation results in male animals after 168 hours of administration

Group		S	V	L1	M1	H1	L2	M2	H2	C	P
Number of animals		n=5	n=5	n=5	n=5	n=5	n=5	n=5	n=5	n=5	n=5
Observation items within the cage	Piloerection										
	0	5	5	5	5	5	5	5	5	5	5
	1	0	0	0	0	0	0	0	0	0	0
	2	0	0	0	0	0	0	0	0	0	0
	Eyelid closure										
	0	5	5	5	5	5	5	5	5	5	5
	1	0	0	0	0	0	0	0	0	0	0
	2	0	0	0	0	0	0	0	0	0	0
Observation after removal from the cage	Resistance to handling										
	0	5	5	5	5	5	5	5	5	5	5
	1	0	0	0	0	0	0	0	0	0	0
	2	0	0	0	0	0	0	0	0	0	0
	3	0	0	0	0	0	0	0	0	0	0
	Body tension										
	0	0	0	0	0	0	0	0	0	0	0
	1	5	5	5	5	5	5	5	5	5	5
	2	0	0	0	0	0	0	0	0	0	0
	Skin color										
	0	0	0	0	0	0	0	0	0	0	0
	1	5	5	5	5	5	5	5	5	5	5
	2	0	0	0	0	0	0	0	0	0	0
	Lacrimation										
	0	5	5	5	5	5	5	5	5	5	5
	1	0	0	0	0	0	0	0	0	0	0
	Salivation										
	0	5	5	5	5	5	5	5	5	5	5
	1	0	0	0	0	0	0	0	0	0	0
	2	0	0	0	0	0	0	0	0	0	0
	3	0	0	0	0	0	0	0	0	0	0
	4	0	0	0	0	0	0	0	0	0	0

S: 5% glucose, 0 mg/kg
V: Liposome, 0 mg/kg
L1: PTX (7.5 mg/kg) -Rg3 (11.25 mg/kg) -lipo
M1: PTX (15 mg/kg) -Rg3 (22.5 mg/kg) -lipo
H1: PTX (30 mg/kg) -Rg3 (45 mg/kg) -lipo
L2: Rg3 (11.25 mg/kg) -lipo
M2: Rg3 (22.5 mg/kg) -lipo
H2: Rg3 (45 mg/kg) -lipo
C: PTX (15 mg/kg) -lipo
P: positive control group, 12 mg/kg chlorpromazine hydrochloride injection

The results are presented as frequencies. Compared to the 5% glucose group, P > 0.05.

Summary of modified Irwin test observation results in male animals after 168 hours of administration (Continued)

Group		S	V	L1	M1	H1	L2	M2	H2	C	P
Number of animals		n=5	n=5	n=5	n=5	n=5	n=5	n=5	n=5	n=5	n=5
Observation inside the observation box	Awakeness										
	0	0	0	0	0	0	0	0	0	0	0
	1	0	0	0	0	0	0	0	0	0	0
	2	5	5	5	5	5	5	5	5	5	5
	3	0	0	0	0	0	0	0	0	0	0
	4	0	0	0	0	0	0	0	0	0	0
	Loss of balance										
	0	5	5	5	5	5	5	5	5	5	5
	1	0	0	0	0	0	0	0	0	0	0
	2	0	0	0	0	0	0	0	0	0	0
	3	0	0	0	0	0	0	0	0	0	0
	Paralysis										
	0	5	5	5	5	5	5	5	5	5	5
	1	0	0	0	0	0	0	0	0	0	0
	Exophthalmos										
	0	5	5	5	5	5	5	5	5	5	5
	1	0	0	0	0	0	0	0	0	0	0
	2	0	0	0	0	0	0	0	0	0	0
	3	0	0	0	0	0	0	0	0	0	0
	Piloerection										
	0	5	5	5	5	5	5	5	5	5	5
	1	0	0	0	0	0	0	0	0	0	0
	2	0	0	0	0	0	0	0	0	0	0
	Arching of the back										
	0	5	5	5	5	5	5	5	5	5	5
	1	0	0	0	0	0	0	0	0	0	0
	2	0	0	0	0	0	0	0	0	0	0
	3	0	0	0	0	0	0	0	0	0	0
	Writhing										
	0	5	5	5	5	5	5	5	5	5	5
	1	0	0	0	0	0	0	0	0	0	0
	2	0	0	0	0	0	0	0	0	0	0
	3	0	0	0	0	0	0	0	0	0	0

Summary of modified Irwin test observation results in male animals after 168 hours of administration (Continued)

Group		S	V	L1	M1	H1	L2	M2	H2	C	P
Number of animals		n=5	n=5	n=5	n=5	n=5	n=5	n=5	n=5	n=5	n=5
Observation inside the observation box	Shivering										
	0	5	5	5	5	5	5	5	5	5	5
	1	0	0	0	0	0	0	0	0	0	0
	2	0	0	0	0	0	0	0	0	0	0
	3	0	0	0	0	0	0	0	0	0	0
	Wet dog shake-like trembling										
	0	5	5	5	5	5	5	5	5	5	5
	1	0	0	0	0	0	0	0	0	0	0
	2	0	0	0	0	0	0	0	0	0	0
	3	0	0	0	0	0	0	0	0	0	0
	Convulsions										
	0	5	5	5	5	5	5	5	5	5	5
	1	0	0	0	0	0	0	0	0	0	0
	Respiration										
	0	0	0	0	0	0	0	0	0	0	0
	1	0	0	0	0	0	0	0	0	0	0
	2	5	5	5	5	5	5	5	5	5	5
	3	0	0	0	0	0	0	0	0	0	0
	Chewing										
	0	5	5	5	5	5	5	5	5	5	5
	1	0	0	0	0	0	0	0	0	0	0
	2	0	0	0	0	0	0	0	0	0	0
	3	0	0	0	0	0	0	0	0	0	0
	Sniffing										
	0	0	0	0	0	0	0	0	0	0	0
	1	0	0	0	0	0	0	0	0	0	0
	2	0	0	0	0	0	0	0	0	0	0
	3	5	5	5	5	5	5	5	5	5	5
	Hind leg spreading										
	0	5	5	5	5	5	5	5	5	5	5
	1	0	0	0	0	0	0	0	0	0	0

Summary of modified Irwin test observation results in male animals after 168 hours of administration (Continued)

Group		S	V	L1	M1	H1	L2	M2	H2	C	P
Number of animals		n=5	n=5	n=5	n=5	n=5	n=5	n=5	n=5	n=5	n=5
Observation inside the observation box	Body posture										
	0	0	0	0	0	0	0	0	0	0	0
	1	0	0	0	0	0	0	0	0	0	0
	2	5	5	5	5	5	5	5	5	5	5
	3	0	0	0	0	0	0	0	0	0	0
	4	0	0	0	0	0	0	0	0	0	0
	Tail position										
	0	0	0	0	0	0	0	0	0	0	0
	1	5	5	5	5	5	5	5	5	5	5
	2	0	0	0	0	0	0	0	0	0	0
	3	0	0	0	0	0	0	0	0	0	0
	Spontaneous activity										
	0	0	0	0	0	0	0	0	0	0	0
	1	0	0	0	0	0	0	0	0	0	0
	2	5	5	5	5	5	5	5	5	5	5
	3	0	0	0	0	0	0	0	0	0	0
	4	0	0	0	0	0	0	0	0	0	0
	Abnormal gait (Ataxia)										
	0	5	5	5	5	5	5	5	5	5	5
	1	0	0	0	0	0	0	0	0	0	0
	2	0	0	0	0	0	0	0	0	0	0
	3	0	0	0	0	0	0	0	0	0	0
	Grooming										
	0	5	5	5	5	5	5	5	5	5	5
	1	0	0	0	0	0	0	0	0	0	0
	2	0	0	0	0	0	0	0	0	0	0
	3	0	0	0	0	0	0	0	0	0	0
	Rearing										
	0	5	5	5	5	5	5	5	5	5	5
	1	0	0	0	0	0	0	0	0	0	0
	2	0	0	0	0	0	0	0	0	0	0
	3	0	0	0	0	0	0	0	0	0	0

Summary of modified Irwin test observation results in male animals after 168 hours of administration (Continued)

Group		S	V	L1	M1	H1	L2	M2	H2	C	P
Number of animals		n=5	n=5	n=5	n=5	n=5	n=5	n=5	n=5	n=5	n=5
Observation inside the observation box	Scratching										
	0	5	5	5	5	5	5	5	5	5	5
	1	0	0	0	0	0	0	0	0	0	0
	2	0	0	0	0	0	0	0	0	0	0
	3	0	0	0	0	0	0	0	0	0	0
	Twitching										
	0	5	5	5	5	5	5	5	5	5	5
	1	0	0	0	0	0	0	0	0	0	0
	2	0	0	0	0	0	0	0	0	0	0
	3	0	0	0	0	0	0	0	0	0	0
	Eyelid closure										
	0	5	5	5	5	5	5	5	5	5	5
	1	0	0	0	0	0	0	0	0	0	0
	2	0	0		0	0	0	0	0	0	0
	Urination										
	0	4	3	2	2	3	2	2	4	3	2
	1	1	2	3	3	2	3	3	1	2	3
	Defecation										
	0	4	4	5	5	5	3	5	4	4	5
	1	1	1	0	0	0	2	0	1	1	0
	Death										
	0	5	5	5	5	5	5	5	5	5	5
	1	0	0	0	0	0	0	0	0	0	0
Manipulation inside the observation box	Approach response										
	0	0	0	0	0	0	0	0	0	0	0
	1	0	0	0	0	0	0	0	0	0	0
	2	0	0	0	0	0	0	0	0	0	0
	3	5	5	5	5	5	5	5	5	5	5
	4	0	0	0	0	0	0	0	0	0	0
	5	0	0	0	0	0	0	0	0	0	0

Summary of modified Irwin test observation results in male animals after 168 hours of administration (Continued)

Group		S	V	L1	M1	H1	L2	M2	H2	C	P
Number of animals		n=5	n=5	n=5	n=5	n=5	n=5	n=5	n=5	n=5	n=5
Manipulation inside the observation box	Startle response										
	0	0	0	0	0	0	0	0	0	0	0
	1	0	0	0	0	0	0	0	0	0	0
	2	5	5	5	5	5	5	5	5	5	5
	3	0	0	0	0	0	0	0	0	0	0
	Tail suspension test										
	0	0	0	0	0	0	0	0	0	0	0
	1	0	0	0	0	0	0	0	0	0	0
	2	5	5	5	5	5	5	5	5	5	5
	3	0	0	0	0	0	0	0	0	0	0
	4	0	0	0	0	0	0	0	0	0	0
Manipulation outside the observation box	Vocalization due to handling										
	0	0	0	0	0	0	0	0	0	0	0
	1	5	5	5	5	5	5	5	5	5	5
	2	0	0	0	0	0	0	0	0	0	0
	3	0	0	0	0	0	0	0	0	0	0
	Grid test										
	0	0	0	0	0	0	0	0	0	0	0
	1	0	0	0	0	0	0	0	0	0	0
	2	5	5	5	5	5	5	5	5	5	5
	3	0	0	0	0	0	0	0	0	0	0
	4	0	0	0	0	0	0	0	0	0	0
	Visual orientation										
	0	0	0	0	0	0	0	0	0	0	0
	1	0	0	0	0	0	0	0	0	0	0
	2	5	5	5	5	5	5	5	5	5	5
	Righting reflex										
	0	0	0	0	0	0	0	0	0	0	0
	1	0	0	0	0	0	0	0	0	0	0
	2	5	5	5	5	5	5	5	5	5	5

Summary of modified Irwin test observation results in male animals after 168 hours of administration (Continued)

Group		S	V	L1	M1	H1	L2	M2	H2	C	P
Number of animals		n=5	n=5	n=5	n=5	n=5	n=5	n=5	n=5	n=5	n=5
	Corneal reflex										
	0	0	0	0	0	0	0	0	0	0	0
	1	0	0	0	0	0	0	0	0	0	0
	2	5	5	5	5	5	5	5	5	5	5
	Pinna reflex										
	0	0	0	0	0	0	0	0	0	0	0
	1	0	0	0	0	0	0	0	0	0	0
	2	5	5	5	5	5	5	5	5	5	5
	Grasping reflex										
	0	0	0	0	0	0	0	0	0	0	0
	1	0	0	0	0	0	0	0	0	0	0
	2	5	5	5	5	5	5	5	5	5	5
	Flexor reflex										
	0	0	0	0	0	0	0	0	0	0	0
	1	0	0	0	0	0	0	0	0	0	0
	2	5	5	5	5	5	5	5	5	5	5

Table S8. Summary of modified Irwin test observation results in female animals before administration

Group		S	V	L1	M1	H1	L2	M2	H2	C	P
Number of animals		n=5	n=5	n=5	n=5	n=5	n=5	n=5	n=5	n=5	n=5
Observation items within the cage	Piloerection										
	0	5	5	5	5	5	5	5	5	5	5
	1	0	0	0	0	0	0	0	0	0	0
	2	0	0	0	0	0	0	0	0	0	0
	Eyelid closure										
	0	5	5	5	5	5	5	5	5	5	5
	1	0	0	0	0	0	0	0	0	0	0
	2	0	0	0	0	0	0	0	0	0	0
Observation after removal from the cage	Resistance to handling										
	0	0	0	0	0	0	0	0	0	0	0
	1	5	5	5	5	5	5	5	5	5	5
	2	0	0	0	0	0	0	0	0	0	0
	3	0	0	0	0	0	0	0	0	0	0
	Body tension										
	0	0	0	0	0	0	0	0	0	0	0
	1	5	5	5	5	5	5	5	5	5	5
	2	0	0	0	0	0	0	0	0	0	0
	Skin color										
	0	0	0	0	0	0	0	0	0	0	0
	1	5	5	5	5	5	5	5	5	5	5
	2	0	0	0	0	0	0	0	0	0	0
	Lacrimation										
	0	5	5	5	5	5	5	5	5	5	5
	1	0	0	0	0	0	0	0	0	0	0
	Salivation										
	0	5	5	5	5	5	5	5	5	5	5
	1	0	0	0	0	0	0	0	0	0	0
	2	0	0	0	0	0	0	0	0	0	0
	3	0	0	0	0	0	0	0	0	0	0
	4	0	0	0	0	0	0	0	0	0	0

S: 5% glucose, 0 mg/kg
V: Liposome, 0 mg/kg
L1: PTX (7.5 mg/kg) -Rg3 (11.25 mg/kg) -lipo
M1: PTX (15 mg/kg) -Rg3 (22.5 mg/kg) -lipo
H1: PTX (30 mg/kg) -Rg3 (45 mg/kg) -lipo
L2: Rg3 (11.25 mg/kg) -lipo
M2: Rg3 (22.5 mg/kg) -lipo
H2: Rg3 (45 mg/kg) -lipo
C: PTX (15 mg/kg) -lipo
P: positive control group, 12 mg/kg chlorpromazine hydrochloride injection

The results are presented as frequencies. Compared to the 5% glucose group, $P > 0.05$.

Summary of modified Irwin test observation results in female animals before administration (Continued)

Group		S	V	L1	M1	H1	L2	M2	H2	C	P
Number of animals		n=5	n=5	n=5	n=5	n=5	n=5	n=5	n=5	n=5	n=5
Observation inside the observation box	Awakeness										
	0	0	0	0	0	0	0	0	0	0	0
	1	0	0	0	0	0	0	0	0	0	0
	2	5	5	5	5	5	5	5	5	5	5
	3	0	0	0	0	0	0	0	0	0	0
	4	0	0	0	0	0	0	0	0	0	0
	Loss of balance										
	0	5	5	5	5	5	5	5	5	5	5
	1	0	0	0	0	0	0	0	0	0	0
	2	0	0	0	0	0	0	0	0	0	0
	3	0	0	0	0	0	0	0	0	0	0
	Paralysis										
	0	5	5	5	5	5	5	5	5	5	5
	1	0	0	0	0	0	0	0	0	0	0
	Exophthalmos										
	0	5	5	5	5	5	5	5	5	5	5
	1	0	0	0	0	0	0	0	0	0	0
	2	0	0	0	0	0	0	0	0	0	0
	3	0	0	0	0	0	0	0	0	0	0
	Piloerection										
	0	5	5	5	5	5	5	5	5	5	5
	1	0	0	0	0	0	0	0	0	0	0
	2	0	0	0	0	0	0	0	0	0	0
	Arching of the back										
	0	5	5	5	5	5	5	5	5	5	5
	1	0	0	0	0	0	0	0	0	0	0
	2	0	0	0	0	0	0	0	0	0	0
	3	0	0	0	0	0	0	0	0	0	0
	Writhing										
	0	5	5	5	5	5	5	5	5	5	5
	1	0	0	0	0	0	0	0	0	0	0
	2	0	0	0	0	0	0	0	0	0	0
	3	0	0	0	0	0	0	0	0	0	0

Summary of modified Irwin test observation results in female animals before administration (Continued)

Group		S	V	L1	M1	H1	L2	M2	H2	C	P
Number of animals		n=5	n=5	n=5	n=5	n=5	n=5	n=5	n=5	n=5	n=5
Observation inside the observation box	Shivering										
	0	5	5	5	5	5	5	5	5	5	5
	1	0	0	0	0	0	0	0	0	0	0
	2	0	0	0	0	0	0	0	0	0	0
	3	0	0	0	0	0	0	0	0	0	0
	Wet dog shake-like trembling										
	0	5	5	5	5	5	5	5	5	5	5
	1	0	0	0	0	0	0	0	0	0	0
	2	0	0	0	0	0	0	0	0	0	0
	3	0	0	0	0	0	0	0	0	0	0
	Convulsions										
	0	5	5	5	5	5	5	5	5	5	5
	1	0	0	0	0	0	0	0	0	0	0
	Respiration										
	0	0	0	0	0	0	0	0	0	0	0
	1	0	0	0	0	0	0	0	0	0	0
	2	5	5	5	5	5	5	5	5	5	5
	3	0	0	0	0	0	0	0	0	0	0
	Chewing										
	0	5	5	5	5	5	5	5	5	5	5
	1	0	0	0	0	0	0	0	0	0	0
	2	0	0	0	0	0	0	0	0	0	0
	3	0	0	0	0	0	0	0	0	0	0
	Sniffing										
	0	0	0	0	0	0	0	0	0	0	0
	1	0	0	0	0	0	0	0	0	0	0
	2	0	0	0	0	0	0	0	0	0	0
	3	5	5	5	5	5	5	5	5	5	5
	Hind leg spreading										
	0	5	5	5	5	5	5	5	5	5	5
	1	0	0	0	0	0	0	0	0	0	0

Summary of modified Irwin test observation results in female animals before administration (Continued)

Group		S	V	L1	M1	H1	L2	M2	H2	C	P
Number of animals		n=5	n=5	n=5	n=5	n=5	n=5	n=5	n=5	n=5	n=5
Observation inside the observation box	Body posture										
	0	0	0	0	0	0	0	0	0	0	0
	1	0	0	0	0	0	0	0	0	0	0
	2	5	5	5	5	5	5	5	5	5	5
	3	0	0	0	0	0	0	0	0	0	0
	4	0	0	0	0	0	0	0	0	0	0
	Tail position										
	0	0	0	0	0	0	0	0	0	0	0
	1	5	5	5	5	5	5	5	5	5	5
	2	0	0	0	0	0	0	0	0	0	0
	3	0	0	0	0	0	0	0	0	0	0
	Spontaneous activity										
	0	0	0	0	0	0	0	0	0	0	0
	1	0	0	0	0	0	0	0	0	0	0
	2	5	5	5	5	5	5	5	5	5	5
	3	0	0	0	0	0	0	0	0	0	0
	4	0	0	0	0	0	0	0	0	0	0
	Abnormal gait (Ataxia)										
	0	5	5	5	5	5	5	5	5	5	5
	1	0	0	0	0	0	0	0	0	0	0
	2	0	0	0	0	0	0	0	0	0	0
	3	0	0	0	0	0	0	0	0	0	0
	Grooming										
	0	5	5	5	5	5	5	5	5	5	5
	1	0	0	0	0	0	0	0	0	0	0
	2	0	0	0	0	0	0	0	0	0	0
	3	0	0	0	0	0	0	0	0	0	0
	Rearing										
	0	5	5	5	5	5	5	5	5	5	5
	1	0	0	0	0	0	0	0	0	0	0
	2	0	0	0	0	0	0	0	0	0	0
	3	0	0	0	0	0	0	0	0	0	0

Summary of modified Irwin test observation results in female animals before administration (Continued)

Group		S	V	L1	M1	H1	L2	M2	H2	C	P
Number of animals		n=5	n=5	n=5	n=5	n=5	n=5	n=5	n=5	n=5	n=5
Observation inside the observation box	Scratching										
	0	5	5	5	5	5	5	5	5	5	5
	1	0	0	0	0	0	0	0	0	0	0
	2	0	0	0	0	0	0	0	0	0	0
	3	0	0	0	0	0	0	0	0	0	0
	Twitching										
	0	5	5	5	5	5	5	5	5	5	5
	1	0	0	0	0	0	0	0	0	0	0
	2	0	0	0	0	0	0	0	0	0	0
	3	0	0	0	0	0	0	0	0	0	0
	Eyelid closure										
	0	5	5	5	5	5	5	5	5	5	5
	1	0	0	0	0	0	0	0	0	0	0
	2	0	0	0	0	0	0	0	0	0	0
	Urination										
	0	2	4	2	3	3	3	4	2	2	3
	1	3	1	3	2	2	2	1	3	3	2
	Defecation										
	0	3	0	4	2	3	3	3	2	2	3
	1	2	5	1	3	2	2	2	3	3	2
	Death										
	0	5	5	5	5	5	5	5	5	5	5
	1	0	0	0	0	0	0	0	0	0	0
Manipulation inside the observation box	Approach response										
	0	0	0	0	0	0	0	0	0	0	0
	1	0	0	0	0	0	0	0	0	0	0
	2	0	0	0	0	0	0	0	0	0	0
	3	5	5	5	5	5	5	5	5	5	5
	4	0	0	0	0	0	0	0	0	0	0
	5	0	0	0	0	0	0	0	0	0	0

Summary of modified Irwin test observation results in female animals before administration (Continued)

Group		S	V	L1	M1	H1	L2	M2	H2	C	P
Number of animals		n=5	n=5	n=5	n=5	n=5	n=5	n=5	n=5	n=5	n=5
Manipulation inside the observation box	Startle response										
	0	0	0	0	0	0	0	0	0	0	0
	1	0	0	0	0	0	0	0	0	0	0
	2	5	5	5	5	5	5	5	5	5	5
	3	0	0	0	0	0	0	0	0	0	0
	Tail suspension test										
	0	0	0	0	0	0	0	0	0	0	0
	1	0	0	0	0	0	0	0	0	0	0
	2	5	5	5	5	5	5	5	5	5	5
	3	0	0	0	0	0	0	0	0	0	0
	4	0	0	0	0	0	0	0	0	0	0
Manipulation outside the observation box	Vocalization due to handling										
	0	0	0	0	0	0	0	0	0	0	0
	1	5	5	5	5	5	5	5	5	5	5
	2	0	0	0	0	0	0	0	0	0	0
	3	0	0	0	0	0	0	0	0	0	0
	Grid test										
	0	0	0	0	0	0	0	0	0	0	0
	1	0	0	0	0	0	0	0	0	0	0
	2	5	5	5	5	5	5	5	5	5	5
	3	0	0	0	0	0	0	0	0	0	0
	4	0	0	0	0	0	0	0	0	0	0
	Visual orientation										
	0	0	0	0	0	0	0	0	0	0	0
	1	0	0	0	0	0	0	0	0	0	0
	2	5	5	5	5	5	5	5	5	5	5
	Righting reflex										
	0	0	0	0	0	0	0	0	0	0	0
	1	0	0	0	0	0	0	0	0	0	0
	2	5	5	5	5	5	5	5	5	5	5

Summary of modified Irwin test observation results in female animals before administration (Continued)

Group		S	V	L1	M1	H1	L2	M2	H2	C	P
Number of animals		n=5	n=5	n=5	n=5	n=5	n=5	n=5	n=5	n=5	n=5
	Corneal reflex										
	0	0	0	0	0	0	0	0	0	0	0
	1	0	0	0	0	0	0	0	0	0	0
	2	5	5	5	5	5	5	5	5	5	5
	Pinna reflex										
	0	0	0	0	0	0	0	0	0	0	0
	1	0	0	0	0	0	0	0	0	0	0
	2	5	5	5	5	5	5	5	5	5	5
	Grasping reflex										
	0	0	0	0	0	0	0	0	0	0	0
	1	0	0	0	0	0	0	0	0	0	0
	2	5	5	5	5	5	5	5	5	5	5
	Flexor reflex										
	0	0	0	0	0	0	0	0	0	0	0
	1	0	0	0	0	0	0	0	0	0	0
	2	5	5	5	5	5	5	5	5	5	5

Table S9. Summary of modified Irwin test observation results in female animals after 3 minutes of administration

Group		S	V	L1	M1	H1	L2	M2	H2	C	P
Number of animals		n=5	n=5	n=5	n=5	n=5	n=5	n=5	n=5	n=5	n=5
Observation items within the cage	Piloerection	5	5	5	5	5	5	5	5	5	5
	0	0	0	0	0	0	0	0	0	0	0
	1	0	0	0	0	0	0	0	0	0	0
	2										**
	Eyelid closure	5	5	5	5	5	5	5	5	5	0
	0	0	0	0	0	0	0	0	0	0	5
	1	0	0	0	0	0	0	0	0	0	0
	2										
Observation after removal from the cage	Resistance to handling	5	5	5	5	5	5	5	5	5	5
	0	0	0	0	0	0	0	0	0	0	0
	1	0	0	0	0	0	0	0	0	0	0
	2	0	0	0	0	0	0	0	0	0	0
	3										**
	Body tension	0	0	0	0	0	0	0	0	0	5
	0	5	5	5	5	5	5	5	5	5	0
	1	0	0	0	0	0	0	0	0	0	0
	2										
	Skin color	0	0	0	0	0	0	0	0	0	0
	0	5	5	5	5	5	5	5	5	5	5
	1	0	0	0	0	0	0	0	0	0	0
	2										**
	Lacrimation	5	5	5	5	5	5	5	5	5	0
	0	0	0	0	0	0	0	0	0	0	5
	1										
	Salivation	5	5	5	5	5	5	5	5	5	5
	0	0	0	0	0	0	0	0	0	0	0
	1	0	0	0	0	0	0	0	0	0	0
	2	0	0	0	0	0	0	0	0	0	0
	3	0	0	0	0	0	0	0	0	0	0
	4	5	5	5	5	5	5	5	5	5	5

S: 5% glucose, 0 mg/kg
V: Liposome, 0 mg/kg
L1: PTX (7.5 mg/kg) -Rg3 (11.25 mg/kg) -lipo
M1: PTX (15 mg/kg) -Rg3 (22.5 mg/kg) -lipo
H1: PTX (30 mg/kg) -Rg3 (45 mg/kg) -lipo
L2: Rg3 (11.25 mg/kg) -lipo
M2: Rg3 (22.5 mg/kg) -lipo
H2: Rg3 (45 mg/kg) -lipo
C: PTX (15 mg/kg) -lipo
P: positive control group, 12 mg/kg chlorpromazine hydrochloride injection

The results are presented as frequencies. **, Compared to the 5% glucose group $P < 0.01$.

Summary of modified Irwin test observation results in female animals after 3 minutes of administration (Continued)

Group		S	V	L1	M1	H1	L2	M2	H2	C	P
Number of animals		n=5	n=5	n=5	n=5	n=5	n=5	n=5	n=5	n=5	n=5
Observation inside the observation box	Awakeness										**
	0	0	0	0	0	0	0	0	0	0	5
	1	0	0	0	0	0	0	0	0	0	0
	2	5	5	5	5	5	5	5	5	5	0
	3	0	0	0	0	0	0	0	0	0	0
	4	0	0	0	0	0	0	0	0	0	0
	Loss of balance										
	0	5	5	5	5	5	5	5	5	5	5
	1	0	0	0	0	0	0	0	0	0	0
	2	0	0	0	0	0	0	0	0	0	0
	3	0	0	0	0	0	0	0	0	0	0
	Paralysis										
	0	5	5	5	5	5	5	5	5	5	5
	1	0	0	0	0	0	0	0	0	0	0
	Exophthalmos										
	0	5	5	5	5	5	5	5	5	5	5
	1	0	0	0	0	0	0	0	0	0	0
	2	0	0	0	0	0	0	0	0	0	0
	3	0	0	0	0	0	0	0	0	0	0
	Piloerection										
	0	5	5	5	5	5	5	5	5	5	5
	1	0	0	0	0	0	0	0	0	0	0
	2	0	0	0	0	0	0	0	0	0	0
	Arching of the back										
	0	5	5	5	5	5	5	5	5	5	5
	1	0	0	0	0	0	0	0	0	0	0
	2	0	0	0	0	0	0	0	0	0	0
	3	0	0	0	0	0	0	0	0	0	0
	Writhing										
	0	5	5	5	5	5	5	5	5	5	5
	1	0	0	0	0	0	0	0	0	0	0
	2	0	0	0	0	0	0	0	0	0	0
	3	0	0	0	0	0	0	0	0	0	0

Summary of modified Irwin test observation results in female animals after 3 minutes of administration (Continued)

Group		S	V	L1	M1	H1	L2	M2	H2	C	P
Number of animals		n=5	n=5	n=5	n=5	n=5	n=5	n=5	n=5	n=5	n=5
Observation inside the observation box	Shivering										
	0	5	5	5	5	5	5	5	5	5	5
	1	0	0	0	0	0	0	0	0	0	0
	2	0	0	0	0	0	0	0	0	0	0
	3	0	0	0	0	0	0	0	0	0	0
	Wet dog shake-like trembling										
	0	5	5	5	5	5	5	5	5	5	5
	1	0	0	0	0	0	0	0	0	0	0
	2	0	0	0	0	0	0	0	0	0	0
	3	0	0	0	0	0	0	0	0	0	0
	Convulsions										
	0	5	5	5	5	5	5	5	5	5	5
	1	0	0	0	0	0	0	0	0	0	0
	Respiration										**
	0	0	0	0	0	0	0	0	0	0	0
	1	0	0	0	0	0	0	0	0	0	5
	2	5	5	5	5	5	5	5	5	5	0
	3	0	0	0	0	0	0	0	0	0	0
	Chewing										
	0	5	5	5	5	5	5	5	5	5	5
	1	0	0	0	0	0	0	0	0	0	0
	2	0	0	0	0	0	0	0	0	0	0
	3	0	0	0	0	0	0	0	0	0	0
	Sniffing										**
	0	0	0	0	0	0	0	0	0	0	5
	1	0	0	0	0	0	0	0	0	0	0
	2	0	0	0	0	0	0	0	0	0	0
	3	5	5	5	5	5	5	5	5	5	0
	Hind leg spreading										
	0	5	5	5	5	5	5	5	5	5	5
	1	0	0	0	0	0	0	0	0	0	0

Summary of modified Irwin test observation results in female animals after 3 minutes of administration (Continued)

Group		S	V	L1	M1	H1	L2	M2	H2	C	P
Number of animals		n=5	n=5	n=5	n=5	n=5	n=5	n=5	n=5	n=5	n=5
Observation inside the observation box	Body posture										**
	0	0	0	0	0	0	0	0	0	0	5
	1	0	0	0	0	0	0	0	0	0	0
	2	5	5	5	5	5	5	5	5	5	0
	3	0	0	0	0	0	0	0	0	0	0
	4	0	0	0	0	0	0	0	0	0	0
	Tail position										
	0	0	0	0	0	0	0	0	0	0	0
	1	5	5	5	5	5	5	5	5	5	5
	2	0	0	0	0	0	0	0	0	0	0
	3	0	0	0	0	0	0	0	0	0	0
	Spontaneous activity										**
	0	0	0	0	0	0	0	0	0	0	5
	1	0	0	0	0	0	0	0	0	0	0
	2	5	5	5	5	5	5	5	5	5	0
	3	0	0	0	0	0	0	0	0	0	0
	4	0	0	0	0	0	0	0	0	0	0
	Abnormal gait (Ataxia)										
	0	5	5	5	5	5	5	5	5	5	5
	1	0	0	0	0	0	0	0	0	0	0
	2	0	0	0	0	0	0	0	0	0	0
	3	0	0	0	0	0	0	0	0	0	0
	Grooming										
	0	5	5	5	5	5	5	5	5	5	5
	1	0	0	0	0	0	0	0	0	0	0
	2	0	0	0	0	0	0	0	0	0	0
	3	0	0	0	0	0	0	0	0	0	0
	Rearing										
	0	5	5	5	5	5	5	5	5	5	5
	1	0	0	0	0	0	0	0	0	0	0
	2	0	0	0	0	0	0	0	0	0	0
	3	0	0	0	0	0	0	0	0	0	0

Summary of modified Irwin test observation results in female animals after 3 minutes of administration (Continued)

Group		S	V	L1	M1	H1	L2	M2	H2	C	P
Number of animals		n=5	n=5	n=5	n=5	n=5	n=5	n=5	n=5	n=5	n=5
Observation inside the observation box	Scratching										
	0	5	5	5	5	5	5	5	5	5	5
	1	0	0	0	0	0	0	0	0	0	0
	2	0	0	0	0	0	0	0	0	0	0
	3	0	0	0	0	0	0	0	0	0	0
	Twitching										
	0	5	5	5	5	5	5	5	5	5	5
	1	0	0	0	0	0	0	0	0	0	0
	2	0	0	0	0	0	0	0	0	0	0
	3	0	0	0	0	0	0	0	0	0	0
	Eyelid closure										**
	0	5	5	5	5	5	5	5	5	5	0
	1	0	0	0	0	0	0	0	0	0	5
	2	0	0	0	0	0	0	0	0	0	0
	Urination										
	0	4	4	3	1	5	5	3	5	5	5
	1	1	1	2	4	0	0	2	0	0	0
	Defecation										
	0	4	3	2	3	4	4	5	5	4	5
	1	1	2	3	2	1	1	0	0	1	0
	Death										
	0	5	5	5	5	5	5	5	5	5	5
	1	0	0	0	0	0	0	0	0	0	0
Manipulation inside the observation box	Approach response										**
	0	0	0	0	0	0	0	0	0	0	5
	1	0	0	0	0	0	0	0	0	0	0
	2	0	0	0	0	0	0	0	0	0	0
	3	5	5	5	5	5	5	5	5	5	0
	4	0	0	0	0	0	0	0	0	0	0
	5	0	0	0	0	0	0	0	0	0	0

Summary of modified Irwin test observation results in female animals after 3 minutes of administration (Continued)

Group		S	V	L1	M1	H1	L2	M2	H2	C	P
Number of animals		n=5	n=5	n=5	n=5	n=5	n=5	n=5	n=5	n=5	n=5
Manipulation inside the observation box	Startle response										**
	0	0	0	0	0	0	0	0	0	0	5
	1	0	0	0	0	0	0	0	0	0	0
	2	5	5	5	5	5	5	5	5	5	0
	3	0	0	0	0	0	0	0	0	0	0
	Tail suspension test										**
	0	0	0	0	0	0	0	0	0	0	0
	1	0	0	0	0	0	0	0	0	0	5
	2	5	5	5	5	5	5	5	5	5	0
	3	0	0	0	0	0	0	0	0	0	0
	4	0	0	0	0	0	0	0	0	0	0
Manipulation outside the observation box	Vocalization due to handling										**
	0	0	0	0	0	0	0	0	0	0	5
	1	5	5	5	5	5	5	5	5	5	0
	2	0	0	0	0	0	0	0	0	0	0
	3	0	0	0	0	0	0	0	0	0	0
	Grid test										**
	0	0	0	0	0	0	0	0	0	0	0
	1	0	0	0	0	0	0	0	0	0	0
	2	5	5	5	5	5	5	5	5	5	0
	3	0	0	0	0	0	0	0	0	0	0
	4	0	0	0	0	0	0	0	0	0	5
	Visual orientation										**
	0	0	0	0	0	0	0	0	0	0	5
	1	0	0	0	0	0	0	0	0	0	0
	2	5	5	5	5	5	5	5	5	5	0
	Righting reflex										**
	0	0	0	0	0	0	0	0	0	0	0
	1	0	0	0	0	0	0	0	0	0	5
	2	5	5	5	5	5	5	5	5	5	0

Summary of modified Irwin test observation results in female animals after 3 minutes of administration (Continued)

Group		S	V	L1	M1	H1	L2	M2	H2	C	P
Number of animals		n=5	n=5	n=5	n=5	n=5	n=5	n=5	n=5	n=5	n=5
	Corneal reflex										**
	0	0	0	0	0	0	0	0	0	0	0
	1	0	0	0	0	0	0	0	0	0	5
	2	5	5	5	5	5	5	5	5	5	0
	Pinna reflex										**
	0	0	0	0	0	0	0	0	0	0	5
	1	0	0	0	0	0	0	0	0	0	0
	2	5	5	5	5	5	5	5	5	5	0
	Grasping reflex										**
	0	0	0	0	0	0	0	0	0	0	0
	1	0	0	0	0	0	0	0	0	0	5
	2	5	5	5	5	5	5	5	5	5	0
	Flexor reflex										**
	0	0	0	0	0	0	0	0	0	0	0
	1	0	0	0	0	0	0	0	0	0	5
	2	5	5	5	5	5	5	5	5	5	0

Table S10. Summary of modified Irwin test observation results in female animals after 1 hour of administration

Group		S	V	L1	M1	H1	L2	M2	H2	C	P
Number of animals		n=5	n=5	n=5	n=5	n=5	n=5	n=5	n=5	n=5	n=5
Observation items within the cage	Piloerection										
	0	5	5	5	5	5	5	5	5	5	5
	1	0	0	0	0	0	0	0	0	0	0
	2	0	0	0	0	0	0	0	0	0	0
	Eyelid closure										**
	0	5	5	5	5	5	5	5	5	5	0
	1	0	0	0	0	0	0	0	0	0	5
	2	0	0	0	0	0	0	0	0	0	0
Observation after removal from the cage	Resistance to handling										
	0	5	5	5	5	5	5	5	5	5	5
	1	0	0	0	0	0	0	0	0	0	0
	2	0	0	0	0	0	0	0	0	0	0
	3	0	0	0	0	0	0	0	0	0	0
	Body tension										**
	0	0	0	0	0	0	0	0	0	0	5
	1	5	5	5	5	5	5	5	5	5	0
	2	0	0	0	0	0	0	0	0	0	0
	Skin color										
	0	0	0	0	0	0	0	0	0	0	0
	1	5	5	5	5	5	5	5	5	5	5
	2	0	0	0	0	0	0	0	0	0	0
	Lacrimation										**
	0	5	5	5	5	5	5	5	5	5	0
	1	0	0	0	0	0	0	0	0	0	5
	Salivation										
	0	5	5	5	5	5	5	5	5	5	5
	1	0	0	0	0	0	0	0	0	0	0
	2	0	0	0	0	0	0	0	0	0	0
	3	0	0	0	0	0	0	0	0	0	0
	4	0	0	0	0	0	0	0	0	0	0

S: 5% glucose, 0 mg/kg
V: Liposome, 0 mg/kg
L1: PTX (7.5 mg/kg) -Rg3 (11.25 mg/kg) -lipo
M1: PTX (15 mg/kg) -Rg3 (22.5 mg/kg) -lipo
H1: PTX (30 mg/kg) -Rg3 (45 mg/kg) -lipo
L2: Rg3 (11.25 mg/kg) -lipo
M2: Rg3 (22.5 mg/kg) -lipo
H2: Rg3 (45 mg/kg) -lipo
C: PTX (15 mg/kg) -lipo
P: positive control group, 12 mg/kg chlorpromazine hydrochloride injection

The results are presented as frequencies. **, Compared to the 5% glucose group $P < 0.01$.

Summary of modified Irwin test observation results in female animals after 1 hour of administration (Continued)

Group		S	V	L1	M1	H1	L2	M2	H2	C	P
Number of animals		n=5	n=5	n=5	n=5	n=5	n=5	n=5	n=5	n=5	n=5
Observation inside the observation box	Awakeness										**
	0	0	0	0	0	0	0	0	0	0	5
	1	0	0	0	0	0	0	0	0	0	0
	2	5	5	5	5	5	5	5	5	5	0
	3	0	0	0	0	0	0	0	0	0	0
	4	0	0	0	0	0	0	0	0	0	0
	Loss of balance										
	0	5	5	5	5	5	5	5	5	5	5
	1	0	0	0	0	0	0	0	0	0	0
	2	0	0	0	0	0	0	0	0	0	0
	3	0	0	0	0	0	0	0	0	0	0
	Paralysis										
	0	5	5	5	5	5	5	5	5	5	5
	1	0	0	0	0	0	0	0	0	0	0
	Exophthalmos										
	0	5	5	5	5	5	5	5	5	5	5
	1	0	0	0	0	0	0	0	0	0	0
	2	0	0	0	0	0	0	0	0	0	0
	3	0	0	0	0	0	0	0	0	0	0
	Piloerection										
	0	5	5	5	5	5	5	5	5	5	5
	1	0	0	0	0	0	0	0	0	0	0
	2	0	0	0	0	0	0	0	0	0	0
	Arching of the back										
	0	5	5	5	5	5	5	5	5	5	5
	1	0	0	0	0	0	0	0	0	0	0
	2	0	0	0	0	0	0	0	0	0	0
	3	0	0	0	0	0	0	0	0	0	0
	Writhing										
	0	5	5	5	5	5	5	5	5	5	5
	1	0	0	0	0	0	0	0	0	0	0
	2	0	0	0	0	0	0	0	0	0	0
	3	0	0	0	0	0	0	0	0	0	0

Summary of modified Irwin test observation results in female animals after 1 hour of administration (Continued)

Group		S	V	L1	M1	H1	L2	M2	H2	C	P
Number of animals		n=5	n=5	n=5	n=5	n=5	n=5	n=5	n=5	n=5	n=5
Observation inside the observation box	Shivering										
	0	5	5	5	5	5	5	5	5	5	5
	1	0	0	0	0	0	0	0	0	0	0
	2	0	0	0	0	0	0	0	0	0	0
	3	0	0	0	0	0	0	0	0	0	0
	Wet dog shake-like trembling										
	0	5	5	5	5	5	5	5	5	5	5
	1	0	0	0	0	0	0	0	0	0	0
	2	0	0	0	0	0	0	0	0	0	0
	3	0	0	0	0	0	0	0	0	0	0
	Convulsions										
	0	5	5	5	5	5	5	5	5	5	5
	1	0	0	0	0	0	0	0	0	0	0
	Respiration										**
	0	0	0	0	0	0	0	0	0	0	0
	1	0	0	0	0	0	0	0	0	0	5
	2	5	5	5	5	5	5	5	5	5	0
	3	0	0	0	0	0	0	0	0	0	0
	Chewing										
	0	5	5	5	5	5	5	5	5	5	5
	1	0	0	0	0	0	0	0	0	0	0
	2	0	0	0	0	0	0	0	0	0	0
	3	0	0	0	0	0	0	0	0	0	0
	Sniffing										**
	0	0	0	0	0	0	0	0	0	0	5
	1	0	0	0	0	0	0	0	0	0	0
	2	0	0	0	0	0	0	0	0	0	0
	3	5	5	5	5	5	5	5	5	5	0
	Hind leg spreading										
	0	5	5	5	5	5	5	5	5	5	5
	1	0	0	0	0	0	0	0	0	0	0

Summary of modified Irwin test observation results in female animals after 1 hour of administration (Continued)

Group		S	V	L1	M1	H1	L2	M2	H2	C	P
Number of animals		n=5	n=5	n=5	n=5	n=5	n=5	n=5	n=5	n=5	n=5
Observation inside the observation box	Body posture										**
	0	0	0	0	0	0	0	0	0	0	5
	1	0	0	0	0	0	0	0	0	0	0
	2	5	5	5	5	5	5	5	5	5	0
	3	0	0	0	0	0	0	0	0	0	0
	4	0	0	0	0	0	0	0	0	0	0
	Tail position										
	0	0	0	0	0	0	0	0	0	0	0
	1	5	5	5	5	5	5	5	5	5	5
	2	0	0	0	0	0	0	0	0	0	0
	3	0	0	0	0	0	0	0	0	0	0
	Spontaneous activity										**
	0	0	0	0	0	0	0	0	0	0	5
	1	0	0	0	0	0	0	0	0	0	0
	2	5	5	5	5	5	5	5	5	5	0
	3	0	0	0	0	0	0	0	0	0	0
	4	0	0	0	0	0	0	0	0	0	0
	Abnormal gait (Ataxia)										
	0	5	5	5	5	5	5	5	5	5	5
	1	0	0	0	0	0	0	0	0	0	0
	2	0	0	0	0	0	0	0	0	0	0
	3	0	0	0	0	0	0	0	0	0	0
	Grooming										
	0	5	5	5	5	5	5	5	5	5	5
	1	0	0	0	0	0	0	0	0	0	0
	2	0	0	0	0	0	0	0	0	0	0
	3	0	0	0	0	0	0	0	0	0	0
	Rearing										
	0	5	5	5	5	5	5	5	5	5	5
	1	0	0	0	0	0	0	0	0	0	0
	2	0	0	0	0	0	0	0	0	0	0
	3	0	0	0	0	0	0	0	0	0	0

Summary of modified Irwin test observation results in female animals after 1 hour of administration (Continued)

Group		S	V	L1	M1	H1	L2	M2	H2	C	P
Number of animals		n=5	n=5	n=5	n=5	n=5	n=5	n=5	n=5	n=5	n=5
Observation inside the observation box	Scratching										
	0	5	5	5	5	5	5	5	5	5	5
	1	0	0	0	0	0	0	0	0	0	0
	2	0	0	0	0	0	0	0	0	0	0
	3	0	0	0	0	0	0	0	0	0	0
	Twitching										
	0	5	5	5	5	5	5	5	5	5	5
	1	0	0	0	0	0	0	0	0	0	0
	2	0	0	0	0	0	0	0	0	0	0
	3	0	0	0	0	0	0	0	0	0	0
	Eyelid closure										**
	0	5	5	5	5	5	5	5	5	5	0
	1	0	0	0	0	0	0	0	0	0	5
	2	0	0	0	0	0	0	0	0	0	0
	Urination										
	0	5	3	5	3	5	3	4	5	4	5
	1	0	2	0	2	0	2	1	0	1	0
	Defecation										
	0	5	4	3	4	5	5	4	5	4	5
	1	0	1	2	1	0	0	1	0	1	0
	Death										
	0	5	5	5	5	5	5	5	5	5	5
	1	0	0	0	0	0	0	0	0	0	0
Manipulation inside the observation box	Approach response										**
	0	0	0	0	0	0	0	0	0	0	5
	1	0	0	0	0	0	0	0	0	0	0
	2	0	0	0	0	0	0	0	0	0	0
	3	5	5	5	5	5	5	5	5	5	0
	4	0	0	0	0	0	0	0	0	0	0
	5	0	0	0	0	0	0	0	0	0	0

Summary of modified Irwin test observation results in female animals after 1 hour of administration (Continued)

Group		S	V	L1	M1	H1	L2	M2	H2	C	P
Number of animals		n=5	n=5	n=5	n=5	n=5	n=5	n=5	n=5	n=5	n=5
Manipulation inside the observation box	Startle response										**
	0	0	0	0	0	0	0	0	0	0	5
	1	0	0	0	0	0	0	0	0	0	0
	2	5	5	5	5	5	5	5	5	5	0
	3	0	0	0	0	0	0	0	0	0	0
	Tail suspension test										**
	0	0	0	0	0	0	0	0	0	0	0
	1	0	0	0	0	0	0	0	0	0	5
	2	5	5	5	5	5	5	5	5	5	0
	3	0	0	0	0	0	0	0	0	0	0
	4	0	0	0	0	0	0	0	0	0	0
Manipulation outside the observation box	Vocalization due to handling										**
	0	0	0	0	0	0	0	0	0	0	5
	1	5	5	5	5	5	5	5	5	5	0
	2	0	0	0	0	0	0	0	0	0	0
	3	0	0	0	0	0	0	0	0	0	0
	Grid test										**
	0	0	0	0	0	0	0	0	0	0	0
	1	0	0	0	0	0	0	0	0	0	0
	2	5	5	5	5	5	5	5	5	5	0
	3	0	0	0	0	0	0	0	0	0	0
	4	0	0	0	0	0	0	0	0	0	5
	Visual orientation										**
	0	0	0	0	0	0	0	0	0	0	5
	1	0	0	0	0	0	0	0	0	0	0
	2	5	5	5	5	5	5	5	5	5	0
	Righting reflex										**
	0	0	0	0	0	0	0	0	0	0	0
	1	0	0	0	0	0	0	0	0	0	5
	2	5	5	5	5	5	5	5	5	5	0

Summary of modified Irwin test observation results in female animals after 1 hour of administration (Continued)

Group		S	V	L1	M1	H1	L2	M2	H2	C	P
Number of animals		n=5	n=5	n=5	n=5	n=5	n=5	n=5	n=5	n=5	n=5
	Corneal reflex										**
	0	0	0	0	0	0	0	0	0	0	0
	1	0	0	0	0	0	0	0	0	0	5
	2	5	5	5	5	5	5	5	5	5	0
	Pinna reflex										**
	0	0	0	0	0	0	0	0	0	0	5
	1	0	0	0	0	0	0	0	0	0	0
	2	5	5	5	5	5	5	5	5	5	0
	Grasping reflex										**
	0	0	0	0	0	0	0	0	0	0	0
	1	0	0	0	0	0	0	0	0	0	5
	2	5	5	5	5	5	5	5	5	5	0
	Flexor reflex										**
	0	0	0	0	0	0	0	0	0	0	0
	1	0	0	0	0	0	0	0	0	0	5
	2	5	5	5	5	5	5	5	5	5	0

Table S11. Summary of modified Irwin test observation results in female animals after 4 hours of administration

Group		S	V	L1	M1	H1	L2	M2	H2	C	P
Number of animals		n=5	n=5	n=5	n=5	n=5	n=5	n=5	n=5	n=5	n=5
Observation items within the cage	Piloerection										
	0	5	5	5	5	5	5	5	5	5	5
	1	0	0	0	0	0	0	0	0	0	0
	2	0	0	0	0	0	0	0	0	0	0
	Eyelid closure										**
	0	5	5	5	5	5	5	5	5	5	0
	1	0	0	0	0	0	0	0	0	0	5
	2	0	0	0	0	0	0	0	0	0	0
Observation after removal from the cage	Resistance to handling										
	0	5	5	5	5	5	5	5	5	5	5
	1	0	0	0	0	0	0	0	0	0	0
	2	0	0	0	0	0	0	0	0	0	0
	3	0	0	0	0	0	0	0	0	0	0
	Body tension										**
	0	0	0	0	0	0	0	0	0	0	5
	1	5	5	5	5	5	5	5	5	5	0
	2	0	0	0	0	0	0	0	0	0	0
	Skin color										
	0	0	0	0	0	0	0	0	0	0	0
	1	5	5	5	5	5	5	5	5	5	5
	2	0	0	0	0	0	0	0	0	0	0
	Lacrimation										*
	0	5	5	5	5	5	5	5	5	5	2
	1	0	0	0	0	0	0	0	0	0	3
	Salivation										
	0	5	5	5	5	5	5	5	5	5	5
	1	0	0	0	0	0	0	0	0	0	0
	2	0	0	0	0	0	0	0	0	0	0
	3	0	0	0	0	0	0	0	0	0	0
	4	0	0	0	0	0	0	0	0	0	0

S: 5% glucose, 0 mg/kg
V: Liposome, 0 mg/kg
L1: PTX (7.5 mg/kg) -Rg3 (11.25 mg/kg) -lipo
M1: PTX (15 mg/kg) -Rg3 (22.5 mg/kg) -lipo
H1: PTX (30 mg/kg) -Rg3 (45 mg/kg) -lipo
L2: Rg3 (11.25 mg/kg) -lipo
M2: Rg3 (22.5 mg/kg) -lipo
H2: Rg3 (45 mg/kg) -lipo
C: PTX (15 mg/kg) -lipo
P: positive control group, 12 mg/kg chlorpromazine hydrochloride injection

The results are presented as frequencies. **, Compared to the 5% glucose group $P < 0.01$.

Summary of modified Irwin test observation results in female animals after 4 hours of administration (Continued)

Group		S	V	L1	M1	H1	L2	M2	H2	C	P
Number of animals		n=5	n=5	n=5	n=5	n=5	n=5	n=5	n=5	n=5	n=5
Observation inside the observation box	Awakeness										**
	0	0	0	0	0	0	0	0	0	0	5
	1	0	0	0	0	0	0	0	0	0	0
	2	5	5	5	5	5	5	5	5	5	0
	3	0	0	0	0	0	0	0	0	0	0
	4	0	0	0	0	0	0	0	0	0	0
	Loss of balance										
	0	5	5	5	5	5	5	5	5	5	5
	1	0	0	0	0	0	0	0	0	0	0
	2	0	0	0	0	0	0	0	0	0	0
	3	0	0	0	0	0	0	0	0	0	0
	Paralysis										
	0	5	5	5	5	5	5	5	5	5	5
	1	0	0	0	0	0	0	0	0	0	0
	Exophthalmos										
	0	5	5	5	5	5	5	5	5	5	5
	1	0	0	0	0	0	0	0	0	0	0
	2	0	0	0	0	0	0	0	0	0	0
	3	0	0	0	0	0	0	0	0	0	0
	Piloerection										
	0	5	5	5	5	5	5	5	5	5	5
	1	0	0	0	0	0	0	0	0	0	0
	2	0	0	0	0	0	0	0	0	0	0
	Arching of the back										
	0	5	5	5	5	5	5	5	5	5	5
	1	0	0	0	0	0	0	0	0	0	0
	2	0	0	0	0	0	0	0	0	0	0
	3	0	0	0	0	0	0	0	0	0	0
	Writhing										
	0	5	5	5	5	5	5	5	5	5	5
	1	0	0	0	0	0	0	0	0	0	0
	2	0	0	0	0	0	0	0	0	0	0
	3	0	0	0	0	0	0	0	0	0	0

Summary of modified Irwin test observation results in female animals after 4 hours of administration (Continued)

Group		S	V	L1	M1	H1	L2	M2	H2	C	P
Number of animals		n=5	n=5	n=5	n=5	n=5	n=5	n=5	n=5	n=5	n=5
Observation inside the observation box	Shivering										
	0	5	5	5	5	5	5	5	5	5	5
	1	0	0	0	0	0	0	0	0	0	0
	2	0	0	0	0	0	0	0	0	0	0
	3	0	0	0	0	0	0	0	0	0	0
	Wet dog shake-like trembling										
	0	5	5	5	5	5	5	5	5	5	5
	1	0	0	0	0	0	0	0	0	0	0
	2	0	0	0	0	0	0	0	0	0	0
	3	0	0	0	0	0	0	0	0	0	0
	Convulsions										
	0	5	5	5	5	5	5	5	5	5	5
	1	0	0	0	0	0	0	0	0	0	0
	Respiration										**
	0	0	0	0	0	0	0	0	0	0	0
	1	0	0	0	0	0	0	0	0	0	5
	2	5	5	5	5	5	5	5	5	5	0
	3	0	0	0	0	0	0	0	0	0	0
	Chewing										
	0	5	5	5	5	5	5	5	5	5	5
	1	0	0	0	0	0	0	0	0	0	0
	2	0	0	0	0	0	0	0	0	0	0
	3	0	0	0	0	0	0	0	0	0	0
	Sniffing										**
	0	0	0	0	0	0	0	0	0	0	5
	1	0	0	0	0	0	0	0	0	0	0
	2	0	0	0	0	0	0	0	0	0	0
	3	5	5	5	5	5	5	5	5	5	0
	Hind leg spreading										
	0	5	5	5	5	5	5	5	5	5	5
	1	0	0	0	0	0	0	0	0	0	0

Summary of modified Irwin test observation results in female animals after 4 hours of administration (Continued)

Group		S	V	L1	M1	H1	L2	M2	H2	C	P
Number of animals		n=5	n=5	n=5	n=5	n=5	n=5	n=5	n=5	n=5	n=5
Observation inside the observation box	Body posture										**
	0	0	0	0	0	0	0	0	0	0	5
	1	0	0	0	0	0	0	0	0	0	0
	2	5	5	5	5	5	5	5	5	5	0
	3	0	0	0	0	0	0	0	0	0	0
	4	0	0	0	0	0	0	0	0	0	0
	Tail position										
	0	0	0	0	0	0	0	0	0	0	0
	1	5	5	5	5	5	5	5	5	5	5
	2	0	0	0	0	0	0	0	0	0	0
	3	0	0	0	0	0	0	0	0	0	0
	Spontaneous activity										**
	0	0	0	0	0	0	0	0	0	0	5
	1	0	0	0	0	0	0	0	0	0	0
	2	5	5	5	5	5	5	5	5	5	0
	3	0	0	0	0	0	0	0	0	0	0
	4	0	0	0	0	0	0	0	0	0	0
	Abnormal gait (Ataxia)										
	0	5	5	5	5	5	5	5	5	5	5
	1	0	0	0	0	0	0	0	0	0	0
	2	0	0	0	0	0	0	0	0	0	0
	3	0	0	0	0	0	0	0	0	0	0
	Grooming										
	0	5	5	5	5	5	5	5	5	5	5
	1	0	0	0	0	0	0	0	0	0	0
	2	0	0	0	0	0	0	0	0	0	0
	3	0	0	0	0	0	0	0	0	0	0
	Rearing										
	0	5	5	5	5	5	5	5	5	5	5
	1	0	0	0	0	0	0	0	0	0	0
	2	0	0	0	0	0	0	0	0	0	0
	3	0	0	0	0	0	0	0	0	0	0

Summary of modified Irwin test observation results in female animals after 4 hours of administration (Continued)

Group		S	V	L1	M1	H1	L2	M2	H2	C	P
Number of animals		n=5	n=5	n=5	n=5	n=5	n=5	n=5	n=5	n=5	n=5
Observation inside the observation box	Scratching										
	0	5	5	5	5	5	5	5	5	5	5
	1	0	0	0	0	0	0	0	0	0	0
	2	0	0	0	0	0	0	0	0	0	0
	3	0	0	0	0	0	0	0	0	0	0
	Twitching										
	0	5	5	5	5	5	5	5	5	5	5
	1	0	0	0	0	0	0	0	0	0	0
	2	0	0	0	0	0	0	0	0	0	0
	3	0	0	0	0	0	0	0	0	0	0
	Eyelid closure										**
	0	5	5	5	5	5	5	5	5	5	0
	1	0	0	0	0	0	0	0	0	0	5
	2	0	0	0	0	0	0	0	0	0	0
	Urination										
	0	5	3	4	3	3	4	1	4	3	5
	1	0	2	1	2	2	1	4	1	2	0
	Defecation										
	0	5	3	2	4	4	2	4	4	3	5
	1	0	2	3	1	1	3	1	1	2	0
	Death										
	0	5	5	5	5	5	5	5	5	5	5
	1	0	0	0	0	0	0	0	0	0	0
Manipulation inside the observation box	Approach response										**
	0	0	0	0	0	0	0	0	0	0	0
	1	0	0	0	0	0	0	0	0	0	5
	2	0	0	0	0	0	0	0	0	0	0
	3	5	5	5	5	5	5	5	5	5	0
	4	0	0	0	0	0	0	0	0	0	0
	5	0	0	0	0	0	0	0	0	0	0

Summary of modified Irwin test observation results in female animals after 4 hours of administration (Continued)

Group		S	V	L1	M1	H1	L2	M2	H2	C	P
Number of animals		n=5	n=5	n=5	n=5	n=5	n=5	n=5	n=5	n=5	n=5
Manipulation inside the observation box	Startle response										**
	0	0	0	0	0	0	0	0	0	0	0
	1	0	0	0	0	0	0	0	0	0	5
	2	5	5	5	5	5	5	5	5	5	0
	3	0	0	0	0	0	0	0	0	0	0
	Tail suspension test										**
	0	0	0	0	0	0	0	0	0	0	0
	1	0	0	0	0	0	0	0	0	0	5
	2	5	5	5	5	5	5	5	5	5	0
	3	0	0	0	0	0	0	0	0	0	0
	4	0	0	0	0	0	0	0	0	0	0
Manipulation outside the observation box	Vocalization due to handling										**
	0	0	0	0	0	0	0	0	0	0	5
	1	5	5	5	5	5	5	5	5	5	0
	2	0	0	0	0	0	0	0	0	0	0
	3	0	0	0	0	0	0	0	0	0	0
	Grid test										**
	0	0	0	0	0	0	0	0	0	0	0
	1	0	0	0	0	0	0	0	0	0	0
	2	5	5	5	5	5	5	5	5	5	0
	3	0	0	0	0	0	0	0	0	0	0
	4	0	0	0	0	0	0	0	0	0	5
	Visual orientation										
	0	0	0	0	0	0	0	0	0	0	0
	1	0	0	0	0	0	0	0	0	0	0
	2	5	5	5	5	5	5	5	5	5	5
	Righting reflex										**
	0	0	0	0	0	0	0	0	0	0	0
	1	0	0	0	0	0	0	0	0	0	5
	2	5	5	5	5	5	5	5	5	5	0

Summary of modified Irwin test observation results in female animals after 4 hours of administration (Continued)

Group		S	V	L1	M1	H1	L2	M2	H2	C	P
Number of animals		n=5	n=5	n=5	n=5	n=5	n=5	n=5	n=5	n=5	n=5
	Corneal reflex										**
	0	0	0	0	0	0	0	0	0	0	0
	1	0	0	0	0	0	0	0	0	0	5
	2	5	5	5	5	5	5	5	5	5	0
	Pinna reflex										**
	0	0	0	0	0	0	0	0	0	0	0
	1	0	0	0	0	0	0	0	0	0	5
	2	5	5	5	5	5	5	5	5	5	0
	Grasping reflex										**
	0	0	0	0	0	0	0	0	0	0	0
	1	0	0	0	0	0	0	0	0	0	5
	2	5	5	5	5	5	5	5	5	5	0
	Flexor reflex										**
	0	0	0	0	0	0	0	0	0	0	0
	1	0	0	0	0	0	0	0	0	0	5
	2	5	5	5	5	5	5	5	5	5	0

Table S12. Summary of modified Irwin test observation results in female animals after 24 hours of administration

Group		S	V	L1	M1	H1	L2	M2	H2	C	P
Number of animals		n=5	n=5	n=5	n=5	n=5	n=5	n=5	n=5	n=5	n=5
Observation items within the cage	Piloerection	5	5	5	5	5	5	5	5	5	5
	0	0	0	0	0	0	0	0	0	0	0
	1	0	0	0	0	0	0	0	0	0	0
	2										
	Eyelid closure	5	5	5	5	5	5	5	5	5	5
	0	0	0	0	0	0	0	0	0	0	0
	1	0	0	0	0	0	0	0	0	0	0
	2										
Observation after removal from the cage	Resistance to handling	5	5	5	5	5	5	5	5	5	5
	0	0	0	0	0	0	0	0	0	0	0
	1	0	0	0	0	0	0	0	0	0	0
	2	0	0	0	0	0	0	0	0	0	0
	3										
	Body tension	0	0	0	0	0	0	0	0	0	0
	0	5	5	5	5	5	5	5	5	5	5
	1	0	0	0	0	0	0	0	0	0	0
	2										
	Skin color	0	0	0	0	0	0	0	0	0	0
	0	5	5	5	5	5	5	5	5	5	5
	1	0	0	0	0	0	0	0	0	0	0
	2										
	Lacrimation	5	5	5	5	5	5	5	5	5	5
	0	0	0	0	0	0	0	0	0	0	0
	1										
	Salivation	5	5	5	5	5	5	5	5	5	5
	0	0	0	0	0	0	0	0	0	0	0
	1	0	0	0	0	0	0	0	0	0	0
	2	0	0	0	0	0	0	0	0	0	0
	3	0	0	0	0	0	0	0	0	0	0
	4	5	5	5	5	5	5	5	5	5	5

S: 5% glucose, 0 mg/kg
V: Liposome, 0 mg/kg
L1: PTX (7.5 mg/kg) -Rg3 (11.25 mg/kg) -lipo
M1: PTX (15 mg/kg) -Rg3 (22.5 mg/kg) -lipo
H1: PTX (30 mg/kg) -Rg3 (45 mg/kg) -lipo
L2: Rg3 (11.25 mg/kg) -lipo
M2: Rg3 (22.5 mg/kg) -lipo
H2: Rg3 (45 mg/kg) -lipo
C: PTX (15 mg/kg) -lipo
P: positive control group, 12 mg/kg chlorpromazine hydrochloride injection

The results are presented as frequencies. Compared to the 5% glucose group, $P > 0.05$.

Summary of modified Irwin test observation results in female animals after 24 hours of administration (Continued)

Group		S	V	L1	M1	H1	L2	M2	H2	C	P
Number of animals		n=5	n=5	n=5	n=5	n=5	n=5	n=5	n=5	n=5	n=5
Observation inside the observation box	Awakeness										
	0	0	0	0	0	0	0	0	0	0	0
	1	0	0	0	0	0	0	0	0	0	0
	2	5	5	5	5	5	5	5	5	5	5
	3	0	0	0	0	0	0	0	0	0	0
	4	0	0	0	0	0	0	0	0	0	0
	Loss of balance										
	0	5	5	5	5	5	5	5	5	5	5
	1	0	0	0	0	0	0	0	0	0	0
	2	0	0	0	0	0	0	0	0	0	0
	3	0	0	0	0	0	0	0	0	0	0
	Paralysis										
	0	5	5	5	5	5	5	5	5	5	5
	1	0	0	0	0	0	0	0	0	0	0
	Exophthalmos										
	0	5	5	5	5	5	5	5	5	5	5
	1	0	0	0	0	0	0	0	0	0	0
	2	0	0	0	0	0	0	0	0	0	0
	3	0	0	0	0	0	0	0	0	0	0
	Piloerection										
	0	5	5	5	5	5	5	5	5	5	5
	1	0	0	0	0	0	0	0	0	0	0
	2	0	0	0	0	0	0	0	0	0	0
	Arching of the back										
	0	5	5	5	5	5	5	5	5	5	5
	1	0	0	0	0	0	0	0	0	0	0
	2	0	0	0	0	0	0	0	0	0	0
	3	0	0	0	0	0	0	0	0	0	0
	Writhing										
	0	5	5	5	5	5	5	5	5	5	5
	1	0	0	0	0	0	0	0	0	0	0
	2	0	0	0	0	0	0	0	0	0	0
	3	0	0	0	0	0	0	0	0	0	0

Summary of modified Irwin test observation results in female animals after 24 hours of administration (Continued)

Group		S	V	L1	M1	H1	L2	M2	H2	C	P
Number of animals		n=5	n=5	n=5	n=5	n=5	n=5	n=5	n=5	n=5	n=5
Observation inside the observation box	Shivering										
	0	5	5	5	5	5	5	5	5	5	5
	1	0	0	0	0	0	0	0	0	0	0
	2	0	0	0	0	0	0	0	0	0	0
	3	0	0	0	0	0	0	0	0	0	0
	Wet dog shake-like trembling										
	0	5	5	5	5	5	5	5	5	5	5
	1	0	0	0	0	0	0	0	0	0	0
	2	0	0	0	0	0	0	0	0	0	0
	3	0	0	0	0	0	0	0	0	0	0
	Convulsions										
	0	5	5	5	5	5	5	5	5	5	5
	1	0	0	0	0	0	0	0	0	0	0
	Respiration										
	0	0	0	0	0	0	0	0	0	0	0
	1	0	0	0	0	0	0	0	0	0	0
	2	5	5	5	5	5	5	5	5	5	5
	3	0	0	0	0	0	0	0	0	0	0
	Chewing										
	0	5	5	5	5	5	5	5	5	5	5
	1	0	0	0	0	0	0	0	0	0	0
	2	0	0	0	0	0	0	0	0	0	0
	3	0	0	0	0	0	0	0	0	0	0
	Sniffing										
	0	0	0	0	0	0	0	0	0	0	0
	1	0	0	0	0	0	0	0	0	0	0
	2	0	0	0	0	0	0	0	0	0	0
	3	5	5	5	5	5	5	5	5	5	5
	Hind leg spreading										
	0	5	5	5	5	5	5	5	5	5	5
	1	0	0	0	0	0	0	0	0	0	0

Summary of modified Irwin test observation results in female animals after 24 hours of administration (Continued)

Group		S	V	L1	M1	H1	L2	M2	H2	C	P
Number of animals		n=5	n=5	n=5	n=5	n=5	n=5	n=5	n=5	n=5	n=5
Observation inside the observation box	Body posture										
	0	0	0	0	0	0	0	0	0	0	0
	1	0	0	0	0	0	0	0	0	0	0
	2	5	5	5	5	5	5	5	5	5	5
	3	0	0	0	0	0	0	0	0	0	0
	4	0	0	0	0	0	0	0	0	0	0
	Tail position										
	0	0	0	0	0	0	0	0	0	0	0
	1	5	5	5	5	5	5	5	5	5	5
	2	0	0	0	0	0	0	0	0	0	0
	3	0	0	0	0	0	0	0	0	0	0
	Spontaneous activity										
	0	0	0	0	0	0	0	0	0	0	0
	1	0	0	0	0	0	0	0	0	0	0
	2	5	5	5	5	5	5	5	5	5	5
	3	0	0	0	0	0	0	0	0	0	0
	4	0	0	0	0	0	0	0	0	0	0
	Abnormal gait (Ataxia)										
	0	5	5	5	5	5	5	5	5	5	5
	1	0	0	0	0	0	0	0	0	0	0
	2	0	0	0	0	0	0	0	0	0	0
	3	0	0	0	0	0	0	0	0	0	0
	Grooming										
	0	5	5	5	5	5	5	5	5	5	5
	1	0	0	0	0	0	0	0	0	0	0
	2	0	0	0	0	0	0	0	0	0	0
	3	0	0	0	0	0	0	0	0	0	0
	Rearing										
	0	5	5	5	5	5	5	5	5	5	5
	1	0	0	0	0	0	0	0	0	0	0
	2	0	0	0	0	0	0	0	0	0	0
	3	0	0	0	0	0	0	0	0	0	0

Summary of modified Irwin test observation results in female animals after 24 hours of administration (Continued)

Group		S	V	L1	M1	H1	L2	M2	H2	C	P
Number of animals		n=5	n=5	n=5	n=5	n=5	n=5	n=5	n=5	n=5	n=5
Observation inside the observation box	Scratching										
	0	5	5	5	5	5	5	5	5	5	5
	1	0	0	0	0	0	0	0	0	0	0
	2	0	0	0	0	0	0	0	0	0	0
	3	0	0	0	0	0	0	0	0	0	0
	Twitching										
	0	5	5	5	5	5	5	5	5	5	5
	1	0	0	0	0	0	0	0	0	0	0
	2	0	0	0	0	0	0	0	0	0	0
	3	0	0	0	0	0	0	0	0	0	0
	Eyelid closure										
	0	5	5	5	5	5	5	5	5	5	5
	1	0	0	0	0	0	0	0	0	0	0
	2	0	0	0	0	0	0	0	0	0	0
	Urination										
	0	4	4	4	4	4	2	2	2	3	2
	1	1	1	1	1	1	3	3	3	2	3
	Defecation										
	0	4	3	3	3	3	3	1	3	1	3
	1	1	2	2	2	2	2	4	2	4	2
	Death										
	0	5	5	5	5	5	5	5	5	5	5
	1	0	0	0	0	0	0	0	0	0	0
Manipulation inside the observation box	Approach response										
	0	0	0	0	0	0	0	0	0	0	0
	1	0	0	0	0	0	0	0	0	0	0
	2	0	0	0	0	0	0	0	0	0	0
	3	5	5	5	5	5	5	5	5	5	5
	4	0	0	0	0	0	0	0	0	0	0
	5	0	0	0	0	0	0	0	0	0	0

Summary of modified Irwin test observation results in female animals after 24 hours of administration (Continued)

Group		S	V	L1	M1	H1	L2	M2	H2	C	P
Number of animals		n=5	n=5	n=5	n=5	n=5	n=5	n=5	n=5	n=5	n=5
Manipulation inside the observation box	Startle response										
	0	0	0	0	0	0	0	0	0	0	0
	1	0	0	0	0	0	0	0	0	0	0
	2	5	5	5	5	5	5	5	5	5	5
	3	0	0	0	0	0	0	0	0	0	0
	Tail suspension test										
	0	0	0	0	0	0	0	0	0	0	0
	1	0	0	0	0	0	0	0	0	0	0
	2	5	5	5	5	5	5	5	5	5	5
	3	0	0	0	0	0	0	0	0	0	0
	4	0	0	0	0	0	0	0	0	0	0
Manipulation outside the observation box	Vocalization due to handling										
	0	0	0	0	0	0	0	0	0	0	0
	1	5	5	5	5	5	5	5	5	5	5
	2	0	0	0	0	0	0	0	0	0	0
	3	0	0	0	0	0	0	0	0	0	0
	Grid test										
	0	0	0	0	0	0	0	0	0	0	0
	1	0	0	0	0	0	0	0	0	0	0
	2	5	5	5	5	5	5	5	5	5	5
	3	0	0	0	0	0	0	0	0	0	0
	4	0	0	0	0	0	0	0	0	0	0
	Visual orientation										
	0	0	0	0	0	0	0	0	0	0	0
	1	0	0	0	0	0	0	0	0	0	0
	2	5	5	5	5	5	5	5	5	5	5
	Righting reflex										
	0	0	0	0	0	0	0	0	0	0	0
	1	0	0	0	0	0	0	0	0	0	0
	2	5	5	5	5	5	5	5	5	5	5

Summary of modified Irwin test observation results in female animals after 24 hours of administration (Continued)

Group		S	V	L1	M1	H1	L2	M2	H2	C	P
Number of animals		n=5	n=5	n=5	n=5	n=5	n=5	n=5	n=5	n=5	n=5
	Corneal reflex										
	0	0	0	0	0	0	0	0	0	0	0
	1	0	0	0	0	0	0	0	0	0	0
	2	5	5	5	5	5	5	5	5	5	5
	Pinna reflex										
	0	0	0	0	0	0	0	0	0	0	0
	1	0	0	0	0	0	0	0	0	0	0
	2	5	5	5	5	5	5	5	5	5	5
	Grasping reflex										
	0	0	0	0	0	0	0	0	0	0	0
	1	0	0	0	0	0	0	0	0	0	0
	2	5	5	5	5	5	5	5	5	5	5
	Flexor reflex										
	0	0	0	0	0	0	0	0	0	0	0
	1	0	0	0	0	0	0	0	0	0	0
	2	5	5	5	5	5	5	5	5	5	5

Table S13. Summary of modified Irwin test observation results in female animals after 72 hours of administration

Group		S	V	L1	M1	H1	L2	M2	H2	C	P
Number of animals		n=5	n=5	n=5	n=5	n=5	n=5	n=5	n=5	n=5	n=5
Observation items within the cage	Piloerection										
	0	5	5	5	4	5	5	5	5	5	5
	1	0	0	0	0	0	0	0	0	0	0
	2	0	0	0	0	0	0	0	0	0	0
	Eyelid closure										
	0	5	5	5	4	5	5	5	5	5	5
	1	0	0	0	0	0	0	0	0	0	0
	2	0	0	0	0	0	0	0	0	0	0
Observation after removal from the cage	Resistance to handling										
	0	5	5	5	4	5	5	5	5	5	5
	1	0	0	0	0	0	0	0	0	0	0
	2	0	0	0	0	0	0	0	0	0	0
	3	0	0	0	0	0	0	0	0	0	0
	Body tension										
	0	0	0	0	0	0	0	0	0	0	0
	1	5	5	5	4	5	5	5	5	5	5
	2	0	0	0	0	0	0	0	0	0	0
	Skin color										
	0	0	0	0	0	0	0	0	0	0	0
	1	5	5	5	4	5	5	5	5	5	5
	2	0	0	0	0	0	0	0	0	0	0
	Lacrimation										
	0	5	5	5	4	5	5	5	5	5	5
	1	0	0	0	0	0	0	0	0	0	0
	Salivation										
	0	5	5	5	4	5	5	5	5	5	5
	1	0	0	0	0	0	0	0	0	0	0
	2	0	0	0	0	0	0	0	0	0	0
	3	0	0	0	0	0	0	0	0	0	0
	4	0	0	0	0	0	0	0	0	0	0

S: 5% glucose, 0 mg/kg
V: Liposome, 0 mg/kg
L1: PTX (7.5 mg/kg) -Rg3 (11.25 mg/kg) -lipo
M1: PTX (15 mg/kg) -Rg3 (22.5 mg/kg) -lipo
H1: PTX (30 mg/kg) -Rg3 (45 mg/kg) -lipo
L2: Rg3 (11.25 mg/kg) -lipo
M2: Rg3 (22.5 mg/kg) -lipo
H2: Rg3 (45 mg/kg) -lipo
C: PTX (15 mg/kg) -lipo
P: positive control group, 12 mg/kg chlorpromazine hydrochloride injection

The results are presented as frequencies. Compared to the 5% glucose group, $P > 0.05$.

Summary of modified Irwin test observation results in female animals after 72 hours of administration (Continued)

Group		S	V	L1	M1	H1	L2	M2	H2	C	P
Number of animals		n=5	n=5	n=5	n=5	n=5	n=5	n=5	n=5	n=5	n=5
Observation inside the observation box	Awakeness										
	0	0	0	0	0	0	0	0	0	0	0
	1	0	0	0	0	0	0	0	0	0	0
	2	5	5	5	4	5	5	5	5	5	5
	3	0	0	0	0	0	0	0	0	0	0
	4	0	0	0	0	0	0	0	0	0	0
	Loss of balance										
	0	5	5	5	4	5	5	5	5	5	5
	1	0	0	0	0	0	0	0	0	0	0
	2	0	0	0	0	0	0	0	0	0	0
	3	0	0	0	0	0	0	0	0	0	0
	Paralysis										
	0	5	5	5	4	5	5	5	5	5	5
	1	0	0	0	0	0	0	0	0	0	0
	Exophthalmos										
	0	5	5	5	4	5	5	5	5	5	5
	1	0	0	0	0	0	0	0	0	0	0
	2	0	0	0	0	0	0	0	0	0	0
	3	0	0	0	0	0	0	0	0	0	0
	Piloerection										
	0	5	5	5	4	5	5	5	5	5	5
	1	0	0	0	0	0	0	0	0	0	0
	2	0	0	0	0	0	0	0	0	0	0
	Arching of the back										
	0	5	5	5	4	5	5	5	5	5	5
	1	0	0	0	0	0	0	0	0	0	0
	2	0	0	0	0	0	0	0	0	0	0
	3	0	0	0	0	0	0	0	0	0	0
	Writhing										
	0	5	5	5	4	5	5	5	5	5	5
	1	0	0	0	0	0	0	0	0	0	0
	2	0	0	0	0	0	0	0	0	0	0
	3	0	0	0	0	0	0	0	0	0	0

Summary of modified Irwin test observation results in female animals after 72 hours of administration (Continued)

Group		S	V	L1	M1	H1	L2	M2	H2	C	P
Number of animals		n=5	n=5	n=5	n=5	n=5	n=5	n=5	n=5	n=5	n=5
Observation inside the observation box	Shivering										
	0	5	5	5	4	5	5	5	5	5	5
	1	0	0	0	0	0	0	0	0	0	0
	2	0	0	0	0	0	0	0	0	0	0
	3	0	0	0	0	0	0	0	0	0	0
	Wet dog shake-like trembling										
	0	5	5	5	4	5	5	5	5	5	5
	1	0	0	0	0	0	0	0	0	0	0
	2	0	0	0	0	0	0	0	0	0	0
	3	0	0	0	0	0	0	0	0	0	0
	Convulsions										
	0	5	5	5	4	5	5	5	5	5	5
	1	0	0	0	0	0	0	0	0	0	0
	Respiration										
	0	0	0	0	0	0	0	0	0	0	0
	1	0	0	0	0	0	0	0	0	0	0
	2	5	5	5	4	5	5	5	5	5	5
	3	0	0	0	0	0	0	0	0	0	0
	Chewing										
	0	5	5	5	4	5	5	5	5	5	5
	1	0	0	0	0	0	0	0	0	0	0
	2	0	0	0	0	0	0	0	0	0	0
	3	0	0	0	0	0	0	0	0	0	0
	Sniffing										
	0	0	0	0	0	0	0	0	0	0	0
	1	0	0	0	0	0	0	0	0	0	0
	2	0	0	0	0	0	0	0	0	0	0
	3	5	5	5	4	5	5	5	5	5	5
	Hind leg spreading										
	0	5	5	5	4	5	5	5	5	5	5
	1	0	0	0	0	0	0	0	0	0	0

Summary of modified Irwin test observation results in female animals after 72 hours of administration (Continued)

Group		S	V	L1	M1	H1	L2	M2	H2	C	P
Number of animals		n=5	n=5	n=5	n=5	n=5	n=5	n=5	n=5	n=5	n=5
Observation inside the observation box	Body posture										
	0	0	0	0	0	0	0	0	0	0	0
	1	0	0	0	0	0	0	0	0	0	0
	2	5	5	5	4	5	5	5	5	5	5
	3	0	0	0	0	0	0	0	0	0	0
	4	0	0	0	0	0	0	0	0	0	0
	Tail position										
	0	0	0	0	0	0	0	0	0	0	0
	1	5	5	5	4	5	5	5	5	5	5
	2	0	0	0	0	0	0	0	0	0	0
	3	0	0	0	0	0	0	0	0	0	0
	Spontaneous activity										
	0	0	0	0	0	0	0	0	0	0	0
	1	0	0	0	0	0	0	0	0	0	0
	2	5	5	5	4	5	5	5	5	5	5
	3	0	0	0	0	0	0	0	0	0	0
	4	0	0	0	0	0	0	0	0	0	0
	Abnormal gait (Ataxia)										
	0	5	5	5	4	5	5	5	5	5	5
	1	0	0	0	0	0	0	0	0	0	0
	2	0	0	0	0	0	0	0	0	0	0
	3	0	0	0	0	0	0	0	0	0	0
	Grooming										
	0	5	5	5	4	5	5	5	5	5	5
	1	0	0	0	0	0	0	0	0	0	0
	2	0	0	0	0	0	0	0	0	0	0
	3	0	0	0	0	0	0	0	0	0	0
	Rearing										
	0	5	5	5	4	5	5	5	5	5	5
	1	0	0	0	0	0	0	0	0	0	0
	2	0	0	0	0	0	0	0	0	0	0
	3	0	0	0	0	0	0	0	0	0	0

Summary of modified Irwin test observation results in female animals after 72 hours of administration (Continued)

Group		S	V	L1	M1	H1	L2	M2	H2	C	P
Number of animals		n=5	n=5	n=5	n=5	n=5	n=5	n=5	n=5	n=5	n=5
Observation inside the observation box	Scratching										
	0	5	5	5	4	5	5	5	5	5	5
	1	0	0	0	0	0	0	0	0	0	0
	2	0	0	0	0	0	0	0	0	0	0
	3	0	0	0	0	0	0	0	0	0	0
	Twitching										
	0	5	5	5	4	5	5	5	5	5	5
	1	0	0	0	0	0	0	0	0	0	0
	2	0	0	0	0	0	0	0	0	0	0
	3	0	0	0	0	0	0	0	0	0	0
	Eyelid closure										
	0	5	5	5	4	5	5	5	5	5	5
	1	0	0	0	0	0	0	0	0	0	0
	2	0	0	0	0	0	0	0	0	0	0
	Urination										
	0	5	5	5	4	5	5	5	5	5	5
	1	0	0	0	0	0	0	0	0	0	0
	Defecation										
	0	5	5	2	2	5	4	5	4	4	4
	1	0	0	3	2	0	1	0	1	1	1
	Death										
	0	5	5	5	4	5	5	5	5	5	5
	1	0	0	0	0	0	0	0	0	0	0
Manipulation inside the observation box	Approach response										
	0	0	0	0	0	0	0	0	0	0	0
	1	0	0	0	0	0	0	0	0	0	0
	2	0	0	0	0	0	0	0	0	0	0
	3	5	5	5	4	5	5	5	5	5	5
	4	0	0	0	0	0	0	0	0	0	0
	5	0	0	0	0	0	0	0	0	0	0

Summary of modified Irwin test observation results in female animals after 72 hours of administration (Continued)

Group		S	V	L1	M1	H1	L2	M2	H2	C	P
Number of animals		n=5	n=5	n=5	n=5	n=5	n=5	n=5	n=5	n=5	n=5
Manipulation inside the observation box	Startle response										
	0	0	0	0	0	0	0	0	0	0	0
	1	0	0	0	0	0	0	0	0	0	0
	2	5	5	5	4	5	5	5	5	5	5
	3	0	0	0	0	0	0	0	0	0	0
	Tail suspension test										
	0	0	0	0	0	0	0	0	0	0	0
	1	0	0	0	0	0	0	0	0	0	0
	2	5	5	5	4	5	5	5	5	5	5
	3	0	0	0	0	0	0	0	0	0	0
	4	0	0	0	0	0	0	0	0	0	0
Manipulation outside the observation box	Vocalization due to handling										
	0	0	0	0	0	0	0	0	0	0	0
	1	5	5	5	4	5	5	5	5	5	5
	2	0	0	0	0	0	0	0	0	0	0
	3	0	0	0	0	0	0	0	0	0	0
	Grid test										
	0	0	0	0	0	0	0	0	0	0	0
	1	0	0	0	0	0	0	0	0	0	0
	2	5	5	5	4	5	5	5	5	5	5
	3	0	0	0	0	0	0	0	0	0	0
	4	0	0	0	0	0	0	0	0	0	0
	Visual orientation										
	0	0	0	0	0	0	0	0	0	0	0
	1	0	0	0	0	0	0	0	0	0	0
	2	5	5	5	4	5	5	5	5	5	5
	Righting reflex										
	0	0	0	0	0	0	0	0	0	0	0
	1	0	0	0	0	0	0	0	0	0	0
	2	5	5	5	4	5	5	5	5	5	5

Summary of modified Irwin test observation results in female animals after 72 hours of administration (Continued)

Group		S	V	L1	M1	H1	L2	M2	H2	C	P
Number of animals		n=5	n=5	n=5	n=5	n=5	n=5	n=5	n=5	n=5	n=5
	Corneal reflex										
	0	0	0	0	0	0	0	0	0	0	0
	1	0	0	0	0	0	0	0	0	0	0
	2	5	5	5	4	5	5	5	5	5	5
	Pinna reflex										
	0	0	0	0	0	0	0	0	0	0	0
	1	0	0	0	0	0	0	0	0	0	0
	2	5	5	5	4	5	5	5	5	5	5
	Grasping reflex										
	0	0	0	0	0	0	0	0	0	0	0
	1	0	0	0	0	0	0	0	0	0	0
	2	5	5	5	4	5	5	5	5	5	5
	Flexor reflex										
	0	0	0	0	0	0	0	0	0	0	0
	1	0	0	0	0	0	0	0	0	0	0
	2	5	5	5	4	5	5	5	5	5	5

Table S14. Summary of modified Irwin test observation results in female animals after 168 hours of administration

Group		S	V	L1	M1	H1	L2	M2	H2	C	P
Number of animals		n=5	n=5	n=5	n=5	n=5	n=5	n=5	n=5	n=5	n=5
Observation items within the cage	Piloerection										
	0	5	5	5	4	5	5	5	5	5	5
	1	0	0	0	0	0	0	0	0	0	0
	2	0	0	0	0	0	0	0	0	0	0
	Eyelid closure										
	0	5	5	5	4	5	5	5	5	5	5
	1	0	0	0	0	0	0	0	0	0	0
	2	0	0	0	0	0	0	0	0	0	0
Observation after removal from the cage	Resistance to handling										
	0	5	5	5	4	5	5	5	5	5	5
	1	0	0	0	0	0	0	0	0	0	0
	2	0	0	0	0	0	0	0	0	0	0
	3	0	0	0	0	0	0	0	0	0	0
	Body tension										
	0	0	0	0	0	0	0	0	0	0	0
	1	5	5	5	4	5	5	5	5	5	5
	2	0	0	0	0	0	0	0	0	0	0
	Skin color										
	0	0	0	0	0	0	0	0	0	0	0
	1	5	5	5	4	5	5	5	5	5	5
	2	0	0	0	0	0	0	0	0	0	0
	Lacrimation										
	0	5	5	5	4	5	5	5	5	5	5
	1	0	0	0	0	0	0	0	0	0	0
	Salivation										
	0	5	5	5	4	5	5	5	5	5	5
	1	0	0	0	0	0	0	0	0	0	0
	2	0	0	0	0	0	0	0	0	0	0
	3	0	0	0	0	0	0	0	0	0	0
	4	0	0	0	0	0	0	0	0	0	0

S: 5% glucose, 0 mg/kg
V: Liposome, 0 mg/kg
L1: PTX (7.5 mg/kg) -Rg3 (11.25 mg/kg) -lipo
M1: PTX (15 mg/kg) -Rg3 (22.5 mg/kg) -lipo
H1: PTX (30 mg/kg) -Rg3 (45 mg/kg) -lipo
L2: Rg3 (11.25 mg/kg) -lipo
M2: Rg3 (22.5 mg/kg) -lipo
H2: Rg3 (45 mg/kg) -lipo
C: PTX (15 mg/kg) -lipo
P: positive control group, 12 mg/kg chlorpromazine hydrochloride injection

The results are presented as frequencies. Compared to the 5% glucose group, P > 0.05.

Summary of modified Irwin test observation results in female animals after 168 hours of administration (Continued)

Group		S	V	L1	M1	H1	L2	M2	H2	C	P
Number of animals		n=5	n=5	n=5	n=5	n=5	n=5	n=5	n=5	n=5	n=5
Observation inside the observation box	Awakeness										
	0	0	0	0	0	0	0	0	0	0	0
	1	0	0	0	0	0	0	0	0	0	0
	2	5	5	5	4	5	5	5	5	5	5
	3	0	0	0	0	0	0	0	0	0	0
	4	0	0	0	0	0	0	0	0	0	0
	Loss of balance										
	0	5	5	5	4	5	5	5	5	5	5
	1	0	0	0	0	0	0	0	0	0	0
	2	0	0	0	0	0	0	0	0	0	0
	3	0	0	0	0	0	0	0	0	0	0
	Paralysis										
	0	5	5	5	4	5	5	5	5	5	5
	1	0	0	0	0	0	0	0	0	0	0
	Exophthalmos										
	0	5	5	5	4	5	5	5	5	5	5
	1	0	0	0	0	0	0	0	0	0	0
	2	0	0	0	0	0	0	0	0	0	0
	3	0	0	0	0	0	0	0	0	0	0
	Piloerection										
	0	5	5	5	4	5	5	5	5	5	5
	1	0	0	0	0	0	0	0	0	0	0
	2	0	0	0	0	0	0	0	0	0	0
	Arching of the back										
	0	5	5	5	4	5	5	5	5	5	5
	1	0	0	0	0	0	0	0	0	0	0
	2	0	0	0	0	0	0	0	0	0	0
	3	0	0	0	0	0	0	0	0	0	0
	Writhing										
	0	5	5	5	4	5	5	5	5	5	5
	1	0	0	0	0	0	0	0	0	0	0
	2	0	0	0	0	0	0	0	0	0	0
	3	0	0	0	0	0	0	0	0	0	0

Summary of modified Irwin test observation results in female animals after 168 hours of administration (Continued)

Group		S	V	L1	M1	H1	L2	M2	H2	C	P
Number of animals		n=5	n=5	n=5	n=5	n=5	n=5	n=5	n=5	n=5	n=5
Observation inside the observation box	Shivering										
	0	5	5	5	4	5	5	5	5	5	5
	1	0	0	0	0	0	0	0	0	0	0
	2	0	0	0	0	0	0	0	0	0	0
	3	0	0	0	0	0	0	0	0	0	0
	Wet dog shake-like trembling										
	0	5	5	5	4	5	5	5	5	5	5
	1	0	0	0	0	0	0	0	0	0	0
	2	0	0	0	0	0	0	0	0	0	0
	3	0	0	0	0	0	0	0	0	0	0
	Convulsions										
	0	5	5	5	4	5	5	5	5	5	5
	1	0	0	0	0	0	0	0	0	0	0
	Respiration										
	0	0	0	0	0	0	0	0	0	0	0
	1	0	0	0	0	0	0	0	0	0	0
	2	5	5	5	4	5	5	5	5	5	5
	3	0	0	0	0	0	0	0	0	0	0
	Chewing										
	0	5	5	5	4	5	5	5	5	5	5
	1	0	0	0	0	0	0	0	0	0	0
	2	0	0	0	0	0	0	0	0	0	0
	3	0	0	0	0	0	0	0	0	0	0
	Sniffing										
	0	0	0	0	0	0	0	0	0	0	0
	1	0	0	0	0	0	0	0	0	0	0
	2	0	0	0	0	0	0	0	0	0	0
	3	5	5	5	4	5	5	5	5	5	5
	Hind leg spreading										
	0	5	5	5	4	5	5	5	5	5	5
	1	0	0	0	0	0	0	0	0	0	0

Summary of modified Irwin test observation results in female animals after 168 hours of administration (Continued)

Group		S	V	L1	M1	H1	L2	M2	H2	C	P
Number of animals		n=5	n=5	n=5	n=5	n=5	n=5	n=5	n=5	n=5	n=5
Observation inside the observation box	Body posture	0	0	0	0	0	0	0	0	0	0
	0	0	0	0	0	0	0	0	0	0	0
	1	5	5	5	4	5	5	5	5	5	5
	2	0	0	0	0	0	0	0	0	0	0
	3	0	0	0	0	0	0	0	0	0	0
	4										
	Tail position	0	0	0	0	0	0	0	0	0	0
	0	5	5	5	4	5	5	5	5	5	5
	1	0	0	0	0	0	0	0	0	0	0
	2	0	0	0	0	0	0	0	0	0	0
	3										
	Spontaneous activity	0	0	0	0	0	0	0	0	0	0
	0	0	0	0	0	0	0	0	0	0	0
	1	5	5	5	4	5	5	5	5	5	5
	2	0	0	0	0	0	0	0	0	0	0
	3	0	0	0	0	0	0	0	0	0	0
	4										
	Abnormal gait (Ataxia)	5	5	5	4	5	5	5	5	5	5
	0	0	0	0	0	0	0	0	0	0	0
	1	0	0	0	0	0	0	0	0	0	0
	2	0	0	0	0	0	0	0	0	0	0
	3										
	Grooming	5	5	5	4	5	5	5	5	5	5
	0	0	0	0	0	0	0	0	0	0	0
	1	0	0	0	0	0	0	0	0	0	0
	2	0	0	0	0	0	0	0	0	0	0
	3										
	Rearing	5	5	5	4	5	5	5	5	5	5
	0	0	0	0	0	0	0	0	0	0	0
	1	0	0	0	0	0	0	0	0	0	0
	2	0	0	0	0	0	0	0	0	0	0
	3	0	0	0	0	0	0	0	0	0	0

Summary of modified Irwin test observation results in female animals after 168 hours of administration (Continued)

Group		S	V	L1	M1	H1	L2	M2	H2	C	P
Number of animals		n=5	n=5	n=5	n=5	n=5	n=5	n=5	n=5	n=5	n=5
Observation inside the observation box	Scratching										
	0	5	5	5	4	5	5	5	5	5	5
	1	0	0	0	0	0	0	0	0	0	0
	2	0	0	0	0	0	0	0	0	0	0
	3	0	0	0	0	0	0	0	0	0	0
	Twitching										
	0	5	5	5	4	5	5	5	5	5	5
	1	0	0	0	0	0	0	0	0	0	0
	2	0	0	0	0	0	0	0	0	0	0
	3	0	0	0	0	0	0	0	0	0	0
	Eyelid closure										
	0	5	5	5	4	5	5	5	5	5	5
	1	0	0	0	0	0	0	0	0	0	0
	2	0	0	0	0	0	0	0	0	0	0
	Urination										
	0	3	2	3	1	2	3	1	3	3	4
	1	2	3	2	3	3	2	4	2	2	1
	Defecation										
	0	2	5	5	4	5	5	4	5	5	4
	1	3	0	0	0	0	0	1	0	0	1
	Death										
	0	5	5	5	4	5	5	5	5	5	5
	1	0	0	0	0	0	0	0	0	0	0
Manipulation inside the observation box	Approach response										
	0	0	0	0	0	0	0	0	0	0	0
	1	0	0	0	0	0	0	0	0	0	0
	2	0	0	0	0	0	0	0	0	0	0
	3	5	5	5	4	5	5	5	5	5	5
	4	0	0	0	0	0	0	0	0	0	0
	5	0	0	0	0	0	0	0	0	0	0

Summary of modified Irwin test observation results in female animals after 168 hours of administration (Continued)

Group		S	V	L1	M1	H1	L2	M2	H2	C	P
Number of animals		n=5	n=5	n=5	n=5	n=5	n=5	n=5	n=5	n=5	n=5
Manipulation inside the observation box	Startle response										
	0	0	0	0	0	0	0	0	0	0	0
	1	0	0	0	0	0	0	0	0	0	0
	2	5	5	5	4	5	5	5	5	5	5
	3	0	0	0	0	0	0	0	0	0	0
	Tail suspension test										
	0	0	0	0	0	0	0	0	0	0	0
	1	0	0	0	0	0	0	0	0	0	0
	2	5	5	5	4	5	5	5	5	5	5
	3	0	0	0	0	0	0	0	0	0	0
	4	0	0	0	0	0	0	0	0	0	0
Manipulation outside the observation box	Vocalization due to handling										
	0	0	0	0	0	0	0	0	0	0	0
	1	5	5	5	4	5	5	5	5	5	5
	2	0	0	0	0	0	0	0	0	0	0
	3	0	0	0	0	0	0	0	0	0	0
	Grid test										
	0	0	0	0	0	0	0	0	0	0	0
	1	0	0	0	0	0	0	0	0	0	0
	2	5	5	5	4	5	5	5	5	5	5
	3	0	0	0	0	0	0	0	0	0	0
	4	0	0	0	0	0	0	0	0	0	0
	Visual orientation										
	0	0	0	0	0	0	0	0	0	0	0
	1	0	0	0	0	0	0	0	0	0	0
	2	5	5	5	4	5	5	5	5	5	5
	Righting reflex										
	0	0	0	0	0	0	0	0	0	0	0
	1	0	0	0	0	0	0	0	0	0	0
	2	5	5	5	4	5	5	5	5	5	5

Summary of modified Irwin test observation results in female animals after 168 hours of administration (Continued)

Group		S	V	L1	M1	H1	L2	M2	H2	C	P
Number of animals		n=5	n=5	n=5	n=5	n=5	n=5	n=5	n=5	n=5	n=5
	Corneal reflex										
	0	0	0	0	0	0	0	0	0	0	0
	1	0	0	0	0	0	0	0	0	0	0
	2	5	5	5	4	5	5	5	5	5	5
	Pinna reflex										
	0	0	0	0	0	0	0	0	0	0	0
	1	0	0	0	0	0	0	0	0	0	0
	2	5	5	5	4	5	5	5	5	5	5
	Grasping reflex										
	0	0	0	0	0	0	0	0	0	0	0
	1	0	0	0	0	0	0	0	0	0	0
	2	5	5	5	4	5	5	5	5	5	5
	Flexor reflex										
	0	0	0	0	0	0	0	0	0	0	0
	1	0	0	0	0	0	0	0	0	0	0
	2	5	5	5	4	5	5	5	5	5	5

Table S15. Description of observations and scores of the modified Irwin test

Observation item		Score	Description of score
Observation items within the cage	Piloerection	0	Not seen/normal
		1	Coat erect, not bulbous in appearance
		2	Erect and bulbous coat
	Eyelid closure	0	Not seen/normal
		1	Half-closed eyelids
		2	Complete eyelid closure
Observation after removal from the cage	Resistance to handling	0	Unprecedented
		1	Mild resistance
		2	Moderate resistance
		3	Significant resistance
	Body tension	0	Decrease
		1	Normalcy
		2	Increase
	Skin color	0	Pale
		1	Normalcy
		2	Flush
		Recorded if there are any other abnormal colors	
	Lacrimation	0	Not seen/normal
		1	Tearfulness
		Recorded if there are any other abnormal colors	
	Salivation	0	Not seen/normal
		1	Slight dampness in the mandibular region
		2	1/4 dampness in the mandibular region
		3	1/2 dampness in the mandibular region
		4	Total dampness in the mandibular region
Observation inside the observation box	Awakeness	0	No head or body movement
		1	Light movements
		2	Normal head or trunk movements
		3	Increased head or trunk movements
		4	Head or trunk movement to escape the cage
	Loss of balance	0	Not seen/normal
		1	Occasional loss of balance
		2	Increase in body imbalance
		3	Continuous loss of body balance
	Paralysis	0	Not seen/normal
		1	Appeared

Description of observations and scores of the modified Irwin test (Continued)

Observation item		Score	Description of score
Observation inside the observation box	Exophthalmos	0	Not seen/normal
		1	Mild exophthalmos
		2	Moderate exophthalmos
		3	Severe exophthalmos
	Piloerection	0	Not seen/normal
		1	Coat erect, not bulbous in appearance
		2	Erect and bulbous coat
	Arching of the back	0	Not seen/normal
		1	Mild arched back
		2	Moderate arched back
		3	Significant arched back
	Writhing	0	Not seen/normal
		1	Occasional body writhing
		2	Increased body writhing
		3	Continuous body writhing
	Shivering	0	Not seen/normal
		1	Mild shivering
		2	Moderate shivering
		3	Severe shivering
	Wet dog shake-like trembling	0	Not seen/normal
		1	Mild
		2	Moderate
		3	Significant
	Convulsions	0	Not seen/normal
		1	Appeared
	Respiration	0	Difficulty in breathing, shortness of breath, intermittent breathing
		1	Slow but regular breathing
		2	Normal/rapid and regular breathing
		3	Significant increase in respiratory rate
	Chewing	0	Not seen
		1	Occasional chewing
		2	Increased chewing
		3	Sustained chewing

Description of observations and scores of the modified Irwin test (Continued)

Observation item		Score	Description of score
Observation inside the observation box	Sniffing	0	Unprecedented
		1	Occasional sniffing
		2	Increased sniffing
		3	Continuous sniffing
	Hind leg spreading	0	Unprecedented
		1	Appearance of hind leg spreading
	Body posture	0	Flat (= lower head)
		1	Partially flat (= not low head)
		2	Normalcy
		3	Body elevation was present during most of the observation
		4	Tiptoe position/walking most of the time observed
	Tail position	0	Tail dragging when walking or in a flat position
		1	Normal/0°-45°
		2	Elevation/45°-90°
		3	90° (recorded as Straub tail reaction)
	Spontaneous activity	0	No spontaneous activity, even in the presence of stimuli
		1	Decreased spontaneous spatial activity
		2	Normal spontaneous spatial activity
		3	Increased spontaneous spatial activity
		4	Continuous spontaneous spatial activity
	Abnormal gait (Ataxia)	0	Not seen/normal
		1	Mild
		2	Moderate
		3	Significant
	Grooming	0	Unprecedented
		1	Occasional grooming
		2	Increased grooming
		3	Continuous grooming
	Rearing	0	Unprecedented
		1	Occasional upright posture
		2	Increased upright posture
		3	Sustained upright posture

Description of observations and scores of the modified Irwin test (Continued)

Observation item		Score	Description of score
Observation inside the observation box	Scratching	0	Unprecedented
		1	Occasional scratching
		2	Increased scratching
		3	Continuous scratching
	Twitching	0	Unprecedented
		1	Occasional twitching
		2	Increased twitching
		3	Continuous twitching
	Eyelid closure	0	Not seen/normal
		1	Half-closed eyelids
		2	Complete eyelid closure
	Urination	0	Unprecedented
		1	Appeared
		Record if urine appears to be an abnormal color	
	Defecation	0	Unprecedented
		1	Appeared
		Record diarrhea/abnormal color if present	
	Death	0	Not seen/normal
		1	Attendance of death
Manipulation inside the observation box	Approach response	0	Unresponsive to the proximity of a cotton swab
		1	Only head movement, no contact with the swab
		2	Move toward the swab without touching the swab
		3	Normal: approaching and intermittent contact with the swab
		4	Approaching and continuous contact with the swab
		5	Approach and tear the swab
	Startle response	0	Not seen: no response
		1	Low reactivity: weak reaction
		2	Normal reactivity: jerking
		3	High reactivity: jumping with all feet off the ground
	Tail suspension test	0	No motor response
		1	Mild motor reactions
		2	Normal: rapid motor response, but did not escape
		3	Rapid motor response and escape
		4	Rapid motor response and tearing

Description of observations and scores of the modified Irwin test (Continued)

Observation item		Score	Description of score
Manipulation outside the observation box	Vocalization due to handling	0	Unprecedented
		1	Infrequent vocalization
		2	Increased vocalization
		3	Continuous vocalization
	Grid test	0	The animal falls off when placed on the grid
		1	The animal stays on the grid but with some difficulty
		2	Normal: the animal stays on the grid and crawls away quickly
		3	The animal remains motionless on the grid for 3-5 seconds and then crawls away
		4	The animal remains motionless on the grid for more than 15 seconds
	Flexor reflex	0	Disappearance
		1	Moderate injury: the reflex appears in only one paw, or flexor reflexes are diminished in one or both paws
		2	Flexor reflexes are normal
	Righting reflex	0	Disappearance
		1	Moderate injury: the animal slowly returns to a standing position
		2	Normal: the animal immediately returns to a standing position
	Corneal reflex	0	Disappearance
		1	Moderate impairment: the reflex appears in only one eye, or decreased corneal reflexes in one or both eyes.
		2	Normal corneal reflexes
	Pinna reflex	0	Disappearance
		1	Moderate impairment: the reflex appears in only one ear, or decreased pinna reflexes in one or both ears
		2	Normal pinna reflexes
	Grasping reflex	0	Disappearance
		1	Moderate injury: the reflex appears in only one paw, or reflexes are diminished in one or both paws
		2	Normal grasping reflexes

Description of observations and scores of the modified Irwin test (Continued)

Observation item		Score	Description of score
Manipulation outside the observation box	Visual orientation	0	Disappearance
		1	Moderate impairment: visual localization is visible after the tentacles touch the grid
		2	Normal: visual localization occurs before the tentacles touch the grid

Table S16. Antibodies used in this study.

Target	Clone	Fluorophore	Cat. number	Vendor
CD45	30-F11	Brilliant Violet 510	103138	Biolegend
CD3	17A2	APC	100236	Biolegend
CD4	GK1.5	PerCP/Cyanine5.5	100434	Biolegend
CD8	53-6.7	APC/Cyanine7	100714	Biolegend
CD11b	M1/70	Brilliant Violet 510	101263	Biolegend
CD69	H1.2F3	PE/Cyanine7	1981586	Invitrogen
Gr-1	RB6-8C5	APC/Cyanine7	108424	Biolegend
IFN-γ	XMG1.2	PE/Cyanine7	505826	Biolegend
Perforin	S16009A	PE	154306	Biolegend
Foxp3	150D	Alexa Fluor647	320014	Biolegend
Arg1	Met1-Lys322	PE	IC5868P	R&D
IL-10	JES5-16E3	PE/Cyanine7	505026	Biolegend
TNF-α	MP6-XT22	APC	506308	Biolegend
CD206	C068C2	PE	141706	Biolegend
CD11c	N418	PE/Cyanine5	117316	Biolegend
F4/80	BM8	APC	123116	Biolegend
Ly-6C	HK1.4	APC/Cyanine7	128026	Biolegend
Ly-6G	1A8	PE/Cyanine7	127618	Biolegend
I-A/I-E	M5/114.15.2	FITC	107606	Biolegend
CD86	GL-1	PE	105008	Biolegend
CD83	Michel-19	PE/Cyanine7	121518	Biolegend
CD80	16-10A1	APC	104714	Biolegend
Ki67	16A8	FITC	652410	Biolegend
InVivoMAb rat IgG2b isotype control	LTF-2		BE0090	BioXcel

InVivoMAb anti-mouse Ly6G/Ly6C (Gr-1)	RB6-8C5		BE0075	BioXcel
Annexin V		FITC	640945	Biolegend
7-AAD Viability Staining Solution			420404	Biolegend
c-Maf	BLR045F		ab243901	Abcam
Mafb	BLR046F		ab243902	Abcam
Vinculin	E1E9V		13901S	Cell Signaling Technology
anti-rabbit IgG secondary antibody	polyclonal antibody		7074S	Cell Signaling Technology
CD3	145-2C11		100340	Biolegend
CD28	37.51		102116	Biolegend
CellTrace CFSE Cell Proliferation Kit			C34570	Invitrogen

Table S17. Primers used in this study.

Gene	Forward primer (5'-3')	Reverse primer (5'-3')
CD86	TCAATGGGACTGCATATCTGCC	GCCAAAATACTACCAGCTCACT
Arg1	CTCCAAGCCAAAGTCCTTAGAG	GGAGCTGTCATTAGGGACATCA
Mrc1	CTCTGTTCAGCTATTGGACGC	TGGCACTCCCAAACATAATTTGA
Tnf	CAGGCGGTGCCTATGTCTC	CGATCACCCCGAAGTTCAGTAG
Il6	CTGCAAGAGACTTCCATCCAG	AGTGGTATAGACAGGTCTGTTGG
Il10	CTTACTGACTGGCATGAGGATCA	GCAGCTCTAGGAGCATGTGG
Slc2a1	GCAGTTCGGCTATAACACTGG	GCGGTGGTTCCATGTTTGATTG
Slc2a3	ATGGGGACAACGAAGGTGAC	CAGGTGCATTGATGACTCCAG

References

1. T. D. Schmittgen, K. J. Livak, Analyzing real-time PCR data by the comparative C(T) method. *Nature protocols* **3**, 1101-1108 (2008).

2. K. Pelka, M. Hofree, J. H. Chen, S. Sarkizova, J. D. Pirl, V. Jorgji, A. Bejnood, D. Dionne, W. H. Ge, K. H. Xu, S. X. Chao, D. R. Zollinger, D. J. Lieb, J. W. Reeves, C. A. Fuhrman, M. L. Hoang, T. Delorey, L. T. Nguyen, J. Waldman, M. Klapholz, I. Wakiro, O. Cohen, J. Albers, C. S. Smillie, M. S. Cuoco, J. Wu, M. J. Su, J. Yeung, B. Vijaykumar, A. M. Magnuson, N. Asinovski, T. Moll, M. N. Goder-Reiser, A. S. Applebaum, L. K. Brais, L. K. DelloStritto, S. L. Denning, S. T. Phillips, E. K. Hill, J. K. Meehan, D. T. Frederick, T. Sharova, A. Kanodia, E. Z. Todres, J. Jané-Valbuena, M. Biton, B. Izar, C. D. Lambden, T. E. Clancy, R. Bleday, N. Melnitchouk, J. Irani, H. Kunitake, D. L. Berger, A. Srivastava, J. L. Hornick, S. Ogino, A. Rotem, S. Vigneau, B. E. Johnson, R. B. Corcoran, A. H. Sharpe, V. K. Kuchroo, K. Ng, M. Giannakis, L. T. Nieman, G. M. Boland, A. J. Aguirre, A. C. Anderson, O. Rozenblatt-Rosen, A. Regev, N. Hacohen, Spatially organized multicellular immune hubs in human colorectal cancer. *Cell* **184**, 4734-4752.e4720 (2021).
