# Supplementary figures and images for: Rg3-lipo biomimetic delivery of paclitaxel enhances targeting of tumors and myeloid-derived suppressor cells
Source: J Clin Invest. 2024 Nov 15;134(22):e178617. doi: 10.1172/JCI178617 (PMC11563678; doi:10.1172/JCI178617)

Figure 6l

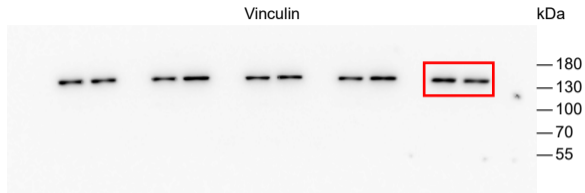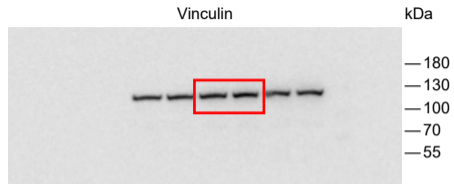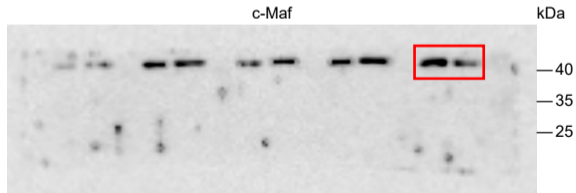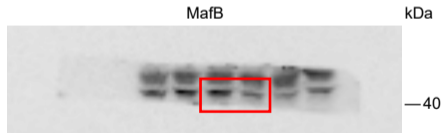

Supplement: Unedited blot and gel images [file jci-134-178617-s039.pdf]
